# Effect of a Combined Drug Approach on the Severity of Ischemia-Reperfusion Injury During Liver Transplant: A Randomized Clinical Trial

**DOI:** 10.1001/jamanetworkopen.2023.0819

**Published:** 2023-02-28

**Authors:** Nicolas Meurisse, Markoen Mertens, Steffen Fieuws, Nicholas Gilbo, Ina Jochmans, Jacques Pirenne, Diethard Monbaliu

**Affiliations:** 1 Laboratory of Abdominal Transplantation, Transplantation Research Group, Department of Microbiology, Immunology and Transplantation, KU Leuven, Leuven, Belgium; 2 Department of Abdominal Transplant Surgery and Transplant Coordination, University Hospitals Leuven, Leuven, Belgium; 3 Department of Abdominal Surgery and Transplantation, CHU de Liège, University of Liège, Liège, Belgium; 4 Interuniversity Institute for Biostatistics and Statistical Bioinformatics, KU Leuven—University of Leuven, Leuven, Belgium

## Abstract

**Importance:**

In a porcine model of liver transplant, a combined drug approach that targeted the donor graft and graft recipient reduced ischemia-reperfusion injury, a major hurdle to the success of liver transplant.

**Objective:**

To assess the effect of a clinical form of a perioperative combined drug approach delivered immediately before implantation to the procured liver and to the liver recipient on the degree of ischemia-reperfusion injury.

**Design, Setting, and Participants:**

This unicentric, investigator-driven, open-label randomized clinical trial with 2 parallel arms was conducted in Belgium from September 2013 through February 2018, with 1-year follow-up. Adults wait-listed for a first solitary full-size liver transplant were screened for eligibility. Exclusion criteria were acute liver failure, kidney failure, contraindication to treatment, participation in another trial, refusal, technical issues, and death while awaiting transplant. Included patients were enrolled and randomized at the time of liver offer. Data were analyzed from May 20, 2019, to May 27, 2020.

**Interventions:**

Participants were randomized to a combined drug approach with standard of care (static cold storage) or standard of care only (control group). In the combined drug approach group, following static cold preservation, donor livers were infused with epoprostenol (ex situ, portal vein); recipients were given oral α-tocopherol and melatonin prior to anesthesia and intravenous antithrombin III, infliximab, apotransferrin, recombinant erythropoietin-β, C1-inhibitor, and glutathione during the anhepatic and reperfusion phase.

**Main Outcomes and Measures:**

The primary outcome was the posttransplant peak serum aspartate aminotransferase (AST) level within the first 72 hours. Secondary end points were the frequencies of postreperfusion syndrome, ischemia-reperfusion injury score, early allograft dysfunction, surgical complications, ischemic cholangiopathy, acute kidney injury, acute cellular rejection, and graft and patient survival.

**Results:**

Of 93 randomized patients, 21 were excluded, resulting in 72 patients (36 per study arm) in the per protocol analysis (median recipient age, 60 years [IQR, 51.7-66.2 years]; 52 [72.2%] men). Peak AST serum levels were not different in the combined drug approach and control groups (geometric mean, 1262.9 U/L [95% CI, 946.3-1685.4 U/L] vs 1451.2 U/L [95% CI, 1087.4-1936.7 U/L]; geometric mean ratio, 0.87 [95% CI, 0.58-1.31]; *P* = .49) (to convert AST to μkat/L, multiply by 0.0167). There also were no significant differences in the secondary end points between the groups.

**Conclusions and Relevance:**

In this randomized clinical trial, the combined drug approach targeting the post–cold storage graft and the recipient did not decrease ischemic-reperfusion injury. The findings suggest that in addition to a downstream strategy that targets the preimplantation liver graft and the graft recipient, a clinically effective combined drug approach may need to include an upstream strategy that targets the donor graft during preservation. Dynamic preservation strategies may provide an appropriate delivery platform.

**Trial Registration:**

ClinicalTrials.gov Identifier: NCT02251041

## Key Points

**Question:**

Can a combined drug approach targeting the preimplantation liver graft and recipient attenuate the degree of ischemia-reperfusion injury?

**Findings:**

In this open-label randomized clinical trial of 72 liver transplant recipients allocated to either static cold storage (standard of care) with an add-on combined drug approach delivered to the preimplantation liver and recipient or to standard of care only, peak aspartate aminotransferase serum levels and other functional, laboratory, and survival outcomes were similar between groups.

**Meaning:**

The findings suggest that use of a downstream combined drug approach that targets the preimplantation liver graft and recipient is not clinically effective for decreasing ischemia-reperfusion injury.

## Teaser

This randomized clinical trial investigates the effect of a combined drug approach targeting the preimplantation liver graft and recipient on the severity of ischemia-reperfusion injury during transplant.

## Introduction

Liver transplant is a life-saving therapy for liver failure, but its success is limited by a marked shortage of organs and high mortality among individuals on the waiting list.^1^ Attempts to improve this imbalance have led to less restrictive organ donation criteria and an expansion of the donor pool with so-called extended-criteria donors, including donors of more advanced age, donors with steatotic livers, donation after circulatory death (DCD), and donor livers exposed to prolonged static cold ischemia time (CIT).^2^

Livers from extended-criteria donors are more susceptible to ischemia-reperfusion injury (IRI),^3^ the principal cause of liver graft dysfunction and primary nonfunction after liver transplant.^4,5,6^ Ischemia-reperfusion injury is a self-amplifying process involving 2 interrelated phases of ischemic and reperfusion injury.^7^ The ischemic insult is characterized by a dysfunction of the mitochondrial respiratory chain,^8^ sinusoidal endothelial cell barrier impairment and Kupffer cell activation,^9^ activation of cell death programs, and alterations in expression of genes, such as hypoxia-inducible factor.^5,8^ At reperfusion, the mitochondrial burst of reactive oxygen species (ROS) overburdens the endogenous antioxidant capacity^9,10^ and ATP depletion deepens, all contributing to cell death.^8,11^ The subsequent expression of danger-associated molecule patterns activates innate immune responses that promote the release of inflammatory cytokines and chemokines and complement and coagulation factors.^7,8,11^ Ischemia-reperfusion injury invariably results in local damage that may lead to graft dysfunction,^5^ ischemic cholangiopathy (IC),^12^ and remote organ dysfunction such as acute kidney injury (AKI).^13,14^ Since IRI is associated with increased short- and long-term morbidity and mortality,^15^ finding strategies to effectively mitigate IRI is considered a research priority in the field of liver transplantation.

Over the past decades, evidence from small- and large-animal models has indicated that pharmacological targeting of IRI pathways is a promising strategy to attenuate IRI,^7,16^ for example, through reduction of ROS and ROS-induced effects, blockade of immune activation, or modulation of cytokine responses.^3^ While, to our knowledge, only 3 randomized clinical trials^17,18,19^ have shown clinical efficacy of single-drug treatment with inhaled nitric oxide, *N*-acetylcysteine, or recombinant P-selectin glycoprotein ligand IgG, none of these agents are used in clinical practice. Since IRI has multiple pathogenetic pathways, a multitarget pharmacological strategy that interferes with several steps in the IRI cascade is likely to be more effective.^16,20,21^ Preclinical evidence to support this comes from work by some of us in a stringent porcine DCD model of liver transplant and severe IRI in which a combined drug approach (CDA) consisting of agents that target well-known pathogenic IRI mechanisms was used.^9^ Streptokinase and epoprostenol were flushed through the liver prior to static cold storage, and the recipient received intravenous glycine, α_1_-acid glycoprotein, mitogen-activated protein kinase inhibitor, α-tocopherol, and glutathione.^10^ The CDA allowed for a steady and spontaneous liver function recovery, eliminated primary nonfunction, and improved recipient survival; it prevented Kupffer cell activation and subsequent tumor necrosis factor α production, stabilized glutathione depletion, and reduced redox-active iron and bile salt toxicity.^10^ In a subsequent mechanistic study, the CDA was found to be associated with reduced expression of inflammation-regulating genes involved in pathways of cytokine activity and apoptosis.^22^

Although advocated over the past decade, to our knowledge, a CDA strategy has so far not been tested in the clinic.^16,20,21^ We conducted a randomized clinical trial (RCT) that assessed the effect of a CDA modified to the clinical setting on the severity of IRI after liver transplant.

## Methods

### Study Design

This investigator-initiated, open-label RCT (Combined Drug Approach to Prevent Ischemia-Reperfusion Injury During Transplantation of Livers [CAPITL]) with 2 parallel arms was conducted between September 2013 and February 2018, with 1-year follow-up, at the University Hospitals Leuven, Belgium. Ethical approval was obtained from the Ethics Committee Research UZ / KU Leuven and the Federal Agency for Medicines and Health Products of Belgium. Before recruitment started, the trial was registered at ClinicalTrials.gov (NCT02251041) and approved by the Centre for Evidence in Transplantation. Written informed consent was obtained from study participants when they entered the transplant waiting list. The study was conducted in accordance with principles of the declaration of Helsinki^23^ and followed the Consolidated Standards of Reporting Trials (CONSORT) reporting guideline. The study protocol is available in Supplement 1.

### Study Population

Adults aged 18 years or older who were wait-listed for a first solitary full-size liver transplant were screened for eligibility at the time of liver offer. Recipients of whole livers from donations after brain death and/or circulatory death (Maastricht category 3^24^) were eligible. Exclusion criteria were acute liver failure as an indication for transplant, dialysis prior to transplant, hypersensitivity or a condition with a specific contraindication to any component of the CDA (Table 1), participation in another clinical trial, technical issues with administration of the CDA, and patient refusal.

**Table 1.** Components, Doses, Mechanisms, and Relevant Experimental and Clinical Evidence of the Combined Drug Approach

Component	Dose	Mechanisms	Experimental and clinical evidence
α-Tocopherol	500 mg	Antioxidant, ROS scavenger, increases the release of glutathione and prostacyclin	Improvement of IRI severity, elimination of primary nonfunction, reduction of inflammatory cytokines such as TNF-α, improvement in liver function, reduction of bile salt toxicity, and increase of survival in a stringent pig DCD model of LT^10^ Improvement of hepatocellular injury, lipid peroxidation, and function in rat liver in situ reperfusion model^25^ Reduction of AST levels and ICU LOS after human partial liver resection^26^ Prevention of inflammation and parenchymal injury in isolated rat liver perfusion model^27^
Melatonin	6 mg	Synergistic antioxidant properties with α-tocopherol and glutathione, ROS scavenger	Decrease of TNF-α production and improvement of hepatocellular injury, function, and microcirculation in rat liver ex situ reperfusion model^28,29^
Epoprostenol	500 μg	Vasodilatation, antioxidant, inhibition of platelet aggregation, reduction of leukocyte activation and adhesion	Included in proof of concept; improvement in IRI severity, elimination of primary nonfunction, reduction of inflammatory cytokines such as TNF-α, improvement in liver function, reduction of bile salt toxicity, and increase in survival in a stringent pig DCD model of LT^10^ Improvement of hepatocellular injury and survival rate in pig liver in situ reperfusion model^30^ Decrease of serum aminotransferase and lactate and sinusoidal congestion in pig LT model^31^ Improvement of hepatocellular injury^32^ and biliary strictures^33^ after administration in donor during human LT
Antithrombin III	3000 IU	Improvement of microcirculation (anticoagulation and vasodilatation), anti-inflammatory, antioxidant	Improvement in creatinine values, malondialdehyde levels, myeloperoxidase activity, and histological damage in rat kidney in situ reperfusion model^34^ Reduction of neutrophil rolling and adhesion in a feline mesentery in situ reperfusion model^35^ Increase in the release of prostacyclin, improvement of blood flow, and decrease of cytokine-induced neutrophil chemoattractant and myeloperoxidase in rat liver in situ reperfusion model^36^ Reduction of graft thrombosis incidence in human simultaneous pancreas kidney transplant^37^
Infliximab	3 mg/kg	Anti-inflammatory blocking TNF-α	Prolonging of long-term survival after human intestinal transplant when used as a component of an immunomodulatory strategy^38^ Improvement of hepatocellular injury and decrease of myeloperoxidase in rat liver in situ reperfusion model^39^ Improvement of hepatocellular injury, apoptosis, and survival in mice liver in situ reperfusion model^40^
Apotransferrin	170 mg/kg	Redox-active iron chelator	Included in proof of concept; improvement of IRI severity, elimination of primary nonfunction, reduction of inflammatory cytokines such as TNF-α, improvement of liver function, reduction of bile salt toxicity, and increase in survival in a stringent pig DCD model of LT^10^ Improvement of kidney reperfusion injury and acute kidney failure, decrease of complement activation and ROS formation in mice in situ reperfusion model^41^
EPO-β	2 Doses of 30 000 IU administered separately	Anti-inflammatory, antiapoptotic, antioxidant	Improvement of hepatocellular injury and apoptosis in pig liver in situ reperfusion model^42^ Reduction of inflammatory cytokine (IL-6 and TNF-α) and hospital LOS after human partial liver resection and Pringle maneuver^43^
C1-inhibitor	1000 U	Inhibition of classic (very strong), lectine (strong), and alternative (strong) pathways of the complement activation, regulation of intrinsic and fibrinolytic pathways of the coagulation cascade, anti-inflammatory	Improvement of hepatocellular injury and function in rat liver in situ reperfusion model^44^ Improvement of hepatocellular injury and decrease of inflammatory cell infiltration and histological damage in pig liver ex situ reperfusion model^45^ Improvement of hepatocellular injury, liver regeneration, and survival in mice liver in situ reperfusion model^46^ Improvement of myocardial injury, hemodynamic parameters, and hospital LOS in human coronary artery bypass grafting^47^

Abbreviations: AST, aspartate aminotransferase; DCD, donation after circulatory death; EPO-β, recombinant erythropoietin-β; ICU, intensive care unit; IL-6, interleukin 6; IRI, ischemia reperfusion injury; LOS, length of stay; LT, liver transplant; ROS, reactive oxygen species; TNF-α, tumor necrosis factor α.

### Randomization

At the time of organ offer, eligible patients were enrolled and randomized. A random allocation sequence was generated using computer-based random numbers with permuted block sizes of 2, 4, and 6. No stratification was performed on a priori–defined potential confounders such as CIT and priority on the waiting list through the Model for End-stage Liver Disease (MELD)^48^ score. Data were recorded in an internet-secure electronic case report form (Eonix) at the University Hospitals Leuven.

### Intervention

As part of the standard of care, all livers were preserved by static cold storage following classic organ procurement. All liver grafts were flushed with 1 L of Institut Georges Lopez (IGL-1) preservation solution through the portal vein ex situ at the end of the back-table preparation at the recipient center. All transplants were done with caval replacement using venovenous bypass. After completion of caval and portal anastomosis, reperfusion of the liver was initiated. The standard of care further included an immunosuppressive regimen consisting of tacrolimus (trough levels between 5 and 8 μg/L), mycophenolate mofetil (500 mg, 2 times), and steroids (tapered to 3 months).^14^

In addition to standard of care, patients in the CDA group received a combination drug regimen that was adapted from the CDA regimen used in the porcine study.^10^ Drugs and doses were selected for their proven IRI-protective effects without drug-related adverse events (Table 1).^10,25,26,27,28,29,30,31,32,33,34,35,36,37,38,39,40,41,42,43,44,45,46,47^ Nine agents were administered in 3 distinct steps and with a specific sequence that, according to pharmacokinetic data, allowed for a peak plasma concentration during the reperfusion (Supplement 1). Recipients were given α-tocopherol (500 mg, 5 mL) and melatonin (6 mg) orally prior to transportation to the operating room. Epoprostenol (500 μg, 50 mL, 2 minutes) was added to 1 L of IGL-1 and administered as part of the ex situ flush of the donor liver at the end of the back-table work before implantation. Last, the recipient was given intravenous antithrombin III (3000 IU, 60 mL, 2 minutes), infliximab (3 mg/kg, 7.5 mL/kg, 3 hours), apotransferrin (170 mg/kg, 3.4 mL/kg, 3 hours), recombinant erythropoietin-β (2 doses of 30 000 IU administered separately, 6-hour interval, 0.6 mL, 2 minutes), C1-inhibitor (1000 IU, 10 mL, 5 minutes), and glutathione (3 g, 20 mL, 2 minutes) during the anhepatic and the reperfusion phase.

### End Point Measures

The primary end point was defined as the difference between the 2 study arms in peak serum aspartate aminotransferase (AST) levels within the first 72 hours after graft reperfusion, an accepted clinical surrogate of liver IRI that correlates with patient and graft survival and postreperfusion liver histological assessment.^49,50^ Serum AST levels were analyzed in the central laboratory of the University Hospitals Leuven by means of a colorimetric method with a 4-U/L detection limit (Hitachi-Roche Modular P, Roche Diagnostics).

Secondary end points included the frequencies of postreperfusion syndrome,^51^ severity of histological IRI,^10,52^ early allograft dysfunction (EAD),^53^ severe postoperative surgical complications (Clavien-Dindo classification ≥3b) within the first 30 days after transplant,^54^ IC,^55^ AKI,^14,56^ acute cellular rejection,^57^ 1-year graft survival, and 1-year patient survival (detailed definitions are provided in Supplement 1).

### Safety

All adverse events were prospectively collected in both groups, categorized as adverse events or serious adverse events, and adjudicated for a possible causal relationship with the treatment. Treatment-related adverse events were considered within the first 7 days following transplant.

### Statistical Analysis

A sample size of 58 patients was needed to detect a 50% reduction in peak AST, as determined with a 2-sided, 2-sample pooled *t* test of the mean ratio with log-normal data with α equal to 5% and 80% power. The assumed coefficient of variation was 116%, as obtained from a series of 308 patients who underwent liver transplant at the University Hospitals Leuven between January 2007 and October 2011. Because we anticipated a dropout rate of 20% and aimed to have complete blocks in the randomization, it was planned to include a total of 72 patients (36 per group) in the study.

Results for the primary end point are reported for the full analysis set and a per protocol set. For secondary outcomes, the results for the per protocol set are given.

For the primary end point, a linear regression model was used on log-transformed values with treatment group as a factor, yielding a ratio of geometric means with 95% CIs. In a sensitivity analysis, MELD score and CIT were added as confounders. The primary end point analysis was repeated in the full analysis set with exclusion of patients who received a DCD liver. Odds ratios and 95% CIs are given for the comparison of frequencies of EAD, AKI, postreperfusion syndrome, and severe surgical complications. Mann-Whitney U tests with the Hodges-Lehmann estimator as effect size were performed to compare acute cellular rejection Banff^58^ and IRI scores. For IC and clinically relevant graft loss, the hazard ratio from a Fine-Gray model treating death without the event of interest as a competing risk is reported as well as the cumulative incidence estimates after 1 year. Death-censored graft loss and recipient mortality were visualized using (the complement of) Kaplan-Meier estimates with (exact) log-rank test. All analyses were performed from May 20, 2019, to May 27, 2020, using SAS software, version 9.4 (SAS Institute Inc). Two-sided *P* < .05 was considered to be statistically significant.

## Results

### Recruitment

**Figure 1.**
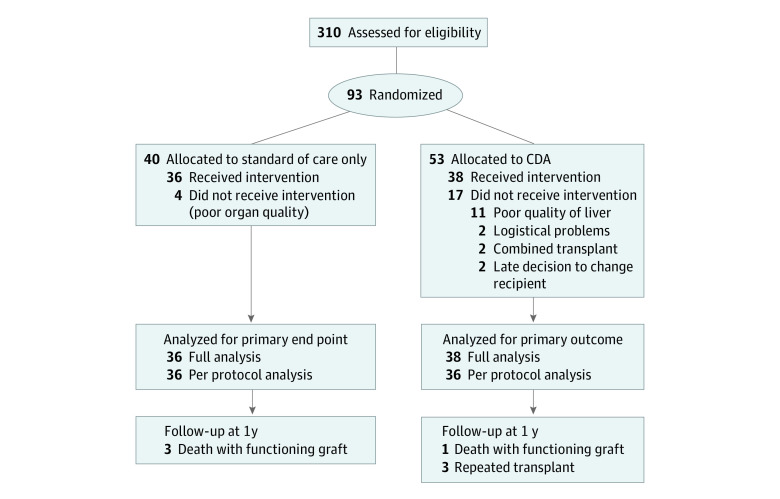
Flow Diagram of Enrollment, Randomization, and Follow-up of Patients CDA indicates combined drug approach.

Between September 2013 and February 2018, 310 consecutive patients were screened for the trial (Figure 1). Of these, 93 patients met the inclusion criteria and were randomized. Due to poor donor liver quality (as assessed by the procuring surgeon), 4 patients were excluded from the control arm and 11 from the CDA arm because livers were discarded and not transplanted. In the CDA arm, 2 patients were excluded because of a last-minute decision to perform a combined liver-kidney transplant (exclusion criterion) and 2 patients were regarded unfit for transplant. As such, the full analysis set included 74 patients (36 in the control arm and 38 in the CDA arm). Two patients did not receive CDA for logistical reasons; hence, the per protocol set contained 36 patients in each arm (median recipient age, 60 years [IQR, 51.7-66.2 years]; 52 men [72.2%] and 20 women [27.8%]). After 60 patients were randomized, it was noted that all postrandomization exclusions up until that moment occurred in the CDA group (n = 8). To avoid a substantial imbalance in group size in the full analysis set, it was decided to create a new sequence of variable block sizes by increasing the probability to be allocated to the CDA group; this yielded a higher number of patients being randomized to the CDA group (53 in the CDA group vs 40 in the control group) but comparable numbers in the full analysis set (38 in the CDA group vs 36 in the control group).

### Donor, Preservation, and Recipient Demographics

Overall, the 2 study arms were balanced with respect to donor and recipient demographics and baseline characteristics (Table 2). The cause of donor death included a higher rate of trauma in the control group than in the CDA group (11 of 36 [30.6%] vs 6 of 38 [15.8%]) and a higher rate of cerebrovascular accident in the CDA group than in the control group (20 of 38 [52.6%] vs 11 of 36 [30.6%]).

**Table 2.** Baseline Donor and Graft Preservation Characteristics and Recipient Characteristics

Characteristic	Participants^a^	
	CDA group (n = 38)	Control group (n = 36)
**Donor and graft preservation**		
Donor age, median (IQR), y	57 (49-70)	59 (48-68)
Donor type		
DBD	26 (68.4)	23 (63.9)
DCD	12 (31.5)	13 (36.1)
Warm ischemia time if DCD, median (IQR), min^b^		
All phases	17 (16-21)	16 (12-25)
Agonal phase^c^	10 (6-13)	9 (2-13)
ICU LOS, median (IQR), d	3 (1-7)	4 (3-7)
Donor cause of death		
Trauma	6 (15.8)	11 (30.6)
CVA	20 (52.6)	11 (30.6)
Anoxia	0	2 (5.6)
Other	12 (31.6)	12 (33.3)
Type of preservation solution		
HTK	6 (15.8)	5 (13.9)
UW	7 (18.4)	5 (13.9)
IGL-1	25 (65.8)	25 (69.4)
Other	0	1 (2.8)
DBD duration, median (IQR), h^d^	13.3 (10.5-17.1)	14.3 (10.0-20.8)
Donor hepatectomy duration, median (IQR), min^e^	34.5 (25.0-46.0)	32.5 (26.0-42.5)
CIT, median (IQR), h^f^	5.8 (4.8-8.1)	5.8 (5.1-8.1)
**Recipient**		
Age, median (IQR), y	59 (53-66)	60 (51-68)
MELD score, median (IQR)^g^	13.2 (8.7-19.1)	13.8 (10.8-19.4)
Indication for live transplant		
Metabolic disease	4 (10.5)	3 (8.3)
HCC	18 (47.4)	12 (33.3)
Chronic liver disease	25 (65.8)	18 (50.0)
Ethyl	23 (60.5)	18 (50.0)
HBV	2 (5.3)	0
HCV	2 (5.3)	0
Cholestatic disease	4 (10.5)	4 (11.1)
NASH	2 (5.3)	10 (27.8)
Cryptogenic	1 (2.6)	0
Implantation duration, median (IQR), min^h^	39.5 (35.0-48.0)	38.5 (34.5-45.0)
ICU LOS, median (IQR), d	3 (1.5-6.5)	4 (2.5-7.0)

Abbreviations: CDA, combined drug approach; CIT, cold ischemia time; CVA, cerebrovascular accident; DBD, donation after brain death; DCD, donation after circulatory death; HBV, hepatitis B virus; HCC, hepatocellular carcinoma; HCV, hepatitis C virus; HTK, histidine tryptophan ketoglutarate; ICU, intensive care unit; IGL, Institut Georges Lopez; LOS, length of stay; MELD, Model for End-stage Liver Disease; NASH, nonalcoholic steatohepatitis; UW, University of Wisconsin.

^a^ Data are presented as number (percentage) of participants unless otherwise indicated.

^b^ Time from withdrawal of life-sustaining therapy to start of cold aortic perfusion in the donor (applied only to DCD liver transplants).

^c^ Time from withdrawal of life-sustaining therapy to circulatory arrest (mean arterial pressure <30 mm Hg).

^d^ Time between the brain death diagnosis and the cold aortic perfusion.

^e^ Time between the start of aortic cold perfusion and completion of the donor hepatectomy, when the liver was placed in ice water on the back table.

^f^ Time between the start of cold aortic perfusion and removal of the liver from cold storage before implantation.

^g^ Possible score range of 4 to 40, with higher scores indicating a higher risk of mortality at 3 months.

^h^ Time required for graft reperfusion between the liver leaving the ice water and completion of portal vein anastomosis.

### Primary End Point

In the per protocol analysis, the peak AST level within the first 72 hours following reperfusion was not different in the CDA group compared with the control group both without adjustment for CIT and MELD score (geometric mean, 1262.9 U/L [95% CI, 946.3-1685.4 U/L] vs 1451.2 U/L [95% CI, 1087.4-1936.7 U/L]; geometric mean ratio, 0.87 [95% CI, 0.58-1.31]; *P* = .49) and with adjustment for these factors (geometric mean, 1261.0 U/L [95% CI, 964.1-1649.5 U/L] vs 1453.3 U/L [95% CI, 1111.0-1901.0 U/L]; geometric mean ratio, 0.87 [95% CI, 0.59-1.27]; *P* = .46) (to convert AST to μkat/L, multiply by 0.0167). Furthermore, subgroup analysis in recipients of livers donated after brain death adjusted for CIT and MELD score did not show any difference in peak AST levels between the CDA and control groups (geometric mean, 1327.6 U/L [95% CI, 946.7-1861.8 U/L] vs 1710.3 U/L [95% CI, 1210.7–2416.1 U/L]; geometric mean ratio, 0.77 [95% CI, 0.48-1.26]; *P* = .29) (Table 3).

**Table 3.** Primary and Secondary End Points

End point	CDA group	Control group	Treatment effect (95% CI)	*P* value
**Primary end point^a^**				
Peak AST level, geometric mean (95% CI), U/L				
PPS results (n = 72)				
Unadjusted	1262.9 (946.3-1685.4)	1451.2 (1087.4-1936.7)	0.87 (0.58-1.31)	.49
Adjusted	1261.0 (964.1-1649.5)	1453.3 (1111.0-1901.0)	0.87 (0.59-1.27)	.46
DBD subgroup				
Unadjusted	1295.6 (907.7-1849.5)	1754.4 (1219.7-2523.6)	0.74 (0.44-1.22)	.23
Adjusted	1327.6 (946.7-1861.8)	1710.3 (1210.7-2416.1)	0.77 (0.48-1.26)	.29
FAS results (n = 74)				
Unadjusted	1223.3 (922.6-1622.1)	1451.2 (1086.0-1939.2)	0.84 (0.56-1.26)	.40
Adjusted	1232.5 (950.5-1598.0)	1439.8 (1102.5-1880.2)	0.86 (0.59-1.24)	.41
**Secondary end points**				
Early allograft dysfunction, No./total No. (%)^b^	13/36 (36.1)	17/36 (47.2)	0.63 (0.24-1.62)	.34
Ischemic cholangiopathy at 1 y, % (95% CI)^c^^,^^d^	11 (3-24)	5 (1-16)	2.05 (0.40-10.45)	.38
Acute kidney injury score, No./total No. (%)^b^^,^^e^				
0	27/34 (79.4)	31/35 (88.5)		
1	4/34 (11.7)	1/35 (2.9)	0.49 (0.31-1.88)	.30
2	2/34 (5.8)	3/35 (8.5)	0.98 (0.12-7.80)	>.99
3	1/34 (2.9)	0	ND	.49
Clinically relevant graft rejection at 1 y, % (95% CI)^c^	13 (5-27)	5 (1-16)	2.5 (0.52-12.05)	.25
IRI score, median (IQR)^f^	2 (2-4)	2 (1-4)	0 (1-0)	.35
Postreperfusion syndrome, No./total No. (%)^b^^,^^g^	7/29 (24.1)	6/29 (20.7)	1.22 (0.35-4.20)	.75
Severe surgical complication, No./total No. (%)^b^	10/36 (27.8)	8/36 (22.2)	1.35 (0.46-3.93)	.79

Abbreviations: AST, aspartate aminotransferase; CDA, combined drug approach; DBD, donation after brain death; FAS, full analysis set; IRI, ischemia-reperfusion injury; ND, not determined because of an absence of events in 1 group; PPS, per protocol set analysis.

SI conversion factor: To convert AST to μkat/L, multiply by 0.0167.

^a^ Treatment effect is expressed as the geometric mean ratio. *P* values are based on unadjusted and adjusted linear regression analysis on log-transformed peak AST values. For the adjusted analysis, the ratio of the geometric means was adjusted for cold ischemia time and Model for End-stage Liver Disease score.

^b^ Treatment effect is expressed as the unadjusted odds ratio and 95% CI. In cases with less than 5 events in at least 1 group, the exact 95% CI and exact *P* value (Fisher test) are reported.

^c^ Treatment effect is expressed as an unadjusted hazard ratio from a Fine-Gray model treating mortality as a competing risk. In each group, the percentage at 1 year derived from the cumulative incidence curve is given.

^d^ Data were missing for 4 in the CDA group and 4 in the control group.

^e^ An acute kidney injury score of 1 indicates risk; 2, injury; and 3, failure. The ordinal character of the score was kept (ie, the reported odds ratios and *P* values refer to the comparison of the probability of having a score of >0, >1, or 3, respectively). Data were missing for 2 in the CDA group and 1 in the control group.

^f^ The treatment effect is the Hodges-Lehmann estimator and equals the median of all pairwise differences. *P* value from a Mann-Whitney U test. Data were missing for 11 in the CDA group and 13 in the control group.

^g^ Data were missing for 7 each in the CDA and control groups.

### Secondary End Points

**Figure 2.**
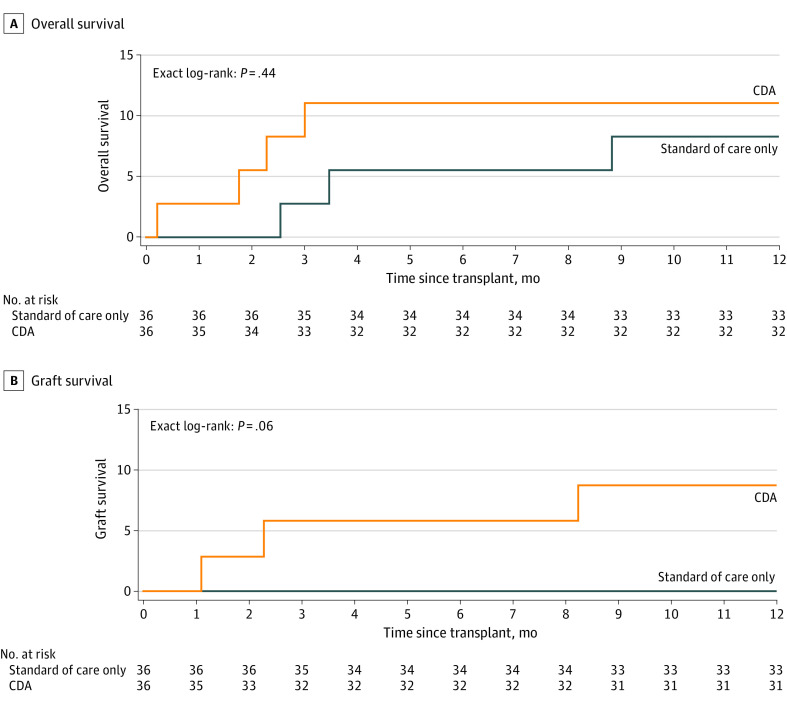
One-year Cumulative Patient Mortality and Graft Loss in the Combined Drug Approach (CDA) and Control Groups

There was no difference in the IRI score or frequencies of postreperfusion syndrome, EAD, severe surgical complications, IC, AKI, and acute cellular rejection between arms (Table 3 and eTable 1 in Supplement 2). Overall, 1-year patient survival was not different between groups (Figure 2A). Four recipients died in the CDA group and 3 in the control group during 1-year follow-up. Three deaths in the CDA group but none in the control group were attributed to graft failure. Death-censored 1-year graft survival was similar between the CDA and control groups (Figure 2B). Graft failure in the CDA group was due to 2 events of mycotic aneurysms and 1 event of IC requiring retransplantation.

### Safety and Adverse Events

A significant difference in the proportion of patients with at least 1 serious adverse event was not observed between groups (10 of 36 [27.8%] in the CDA group and 8 of 36 [22.2%] in the control group; *P* = .79) (eTable 2 in Supplement 2). None of the events were considered to have a causal relationship to the intervention.

## Discussion

In this RCT, we did not find evidence that an add-on perioperative combination drug regimen delivered to the graft immediately before implantation and to the recipient attenuated the degree of IRI. In addition, no differences were found in postreperfusion syndrome, histological IRI score, liver function, and the frequencies of severe surgical complications, IC, AKI, and acute cellular rejection, and graft and patient survival were similar between groups.

These findings were in contrast with those in the proof-of-concept study,^10^ in which a substantial improvement in clinical outcomes was observed, with attenuation of IRI and complete prevention of primary graft nonfunction in a stringent model of porcine DCD liver transplant. The use of pleiotropic treatment strategies is well described and validated in other clinical fields, such as in oncological treatments^59^ or immunosuppressive regimens after transplant.^60^ The outcome of this study was unexpected given the increasingly accepted view that simultaneous targeting of different mechanistic pathways of IRI, through its cumulative and synergistic effects, is more efficient than a single compound approach.^16,20,21,61,62,63^ Several variations, however, arose from the translation of the preclinical proof-of-concept model to the clinical setting and may explain the lack of effect observed in this RCT.

Physiological differences between pigs and humans and the highly controlled and reproducible conditions of experimental models as opposed to the variability in IRI severity in the clinical setting may explain why the protective effect observed in pigs was not reproduced in this study of humans. Another factor to explain the discrepancy between the porcine model and this RCT is that the porcine study^10^ used a primary nonfunction model, which is the extreme clinical manifestation of severe IRI. Such extreme conditions were not encountered in this RCT. In addition, the RCT was powered on a surrogate end point (peak AST) rather than a hard clinical outcome, such as primary nonfunction.

Perhaps the design of the CDA, adapted to the clinical setting, may be another plausible explanation for the difference in results. In the proof-of-concept study,^10^ the CDA was also administered in situ to the donor during the flush prior to static cold storage (streptokinase and epoprostenol) as an upstream strategy (and subsequently during the transplant in the recipient as a downstream strategy) to improve the graft quality during the procurement and preservation. This more upstream strategy could not be applied in this RCT since many donor livers were imported. Of note, at the time when the RCT was conducted, dynamic preservation was still in its infancy. Therefore, a downstream strategy limited to the post–static cold storage phase may not have been sufficient to halt IRI pathways that have already been triggered or the damage that was already established as a result of the ischemic insult during procurement and preservation.^8,11^ Of note, the downstream strategy used in this RCT, which targeted the post–static cold storage graft and the graft recipient, aimed to scavenge ROS and mitigate the ensuing inflammatory cascade but was unable to achieve attenuation of IRI. The addition of an upstream strategy that prevents the formation of mitochondrial ROS by targeting the donor graft before and/or during static cold preservation may be more efficient.^11^ Meanwhile, the importance of maintaining the integrity of mitochondrial function and replenishment of ATP during preservation has been demonstrated in settings where ex situ normothermic^49,64^ and hypothermic oxygenated dynamic preservation^65^ could attenuate liver IRI.

To translate the CDA regimen from the experimental to clinical setting, changes in drug composition were made, which may also have contributed to a variability in effect. The different agents, doses, and time regimens were judiciously chosen based on published evidence of their biological efficacy and pharmacokinetics (Supplement 1). In brief, instead of a streptokinase and epoprostenol flush of the donor pig liver before static cold storage, the human donor liver was perfused via the portal vein with epoprostenol after cold storage only with the goal to improve microcirculatory perfusion.^30,31,32,33^ Since α-tocopherol cannot be used clinically as an intravenous formulation, it was given to the recipient orally prior to transportation to the operative theater, and melatonin was added as a synergistic free-radical scavenger to increase the endogenous antioxidant capacity as it becomes overpowered by the burst of mitochondrial ROS after liver graft reperfusion.^25,26,27,28,29^ The anti-inflammatory agents glycine and mitogen-activated protein kinase inhibitor FR167653 and the antioxidant plasma protein α_1_-acid glycoprotein used in the porcine model are not readily available for clinical use. For this study, they were therefore replaced with infliximab, which is used as part of an immunomodulatory regimen^38^ in intestinal transplant to attenuate ROS production and leukocyte infiltration enhancement,^39,40^ and with recombinant erythropoietin-β to prevent apoptosis, inflammation, and oxidative stress in liver IRI.^42,43^ Antithrombin III was added to reduce microcirculatory disorders and tissue injury,^34,35,36,37^ and complement C1-inhibitor was added to reduce neutrophil infiltration and production of ROS.^44,45,46,47^ Finally, as in the preclinical model, glutathione was included to improve the vascular antioxidant capacity and hepatocyte injury as previously described,^66,67^ and apotransferrin was given to attenuate redox-active iron, which after liver graft reperfusion, is known to exceed the iron-binding capacity of transferrin and to contribute to liver graft failure.^9,10,41^

Recent developments in liver transplantation have included significant progress in the preservation of liver grafts by using dynamic preservation strategies. Preclinical and clinical studies have shown that compared with static cold storage, dynamic preservation allows for a reduction in IRI severity, frequencies of biliary complications and EAD, hospital stay, and graft and patient survival.^49,68,69,70,71,72,73^ As a platform to deliver an upstream conditioning treatment strategy, dynamic preservation may counter or prevent ischemia and maintain mitochondria function^11^ and probably represents a more promising strategy than multidrug strategies targeting post–cold storage grafts and recipients only.

### Limitations

This study has some limitations. First, it was a single-center RCT. Second, individual effects of each component of the CDA in decreasing IRI were not assessed separately. As the CDA specifically aims to achieve synergistic effects, the parallel testing of individual drugs was considered not useful and, in the clinical setting, extremely complex given the multitude of possible combinations. Third, despite the fact that CDA components were administrated according to a specific timing in relation to their pharmacokinetic data with the aim to reach a peak concentration during reperfusion, blood levels of each component were not determined. Fourth, although the study was powered to detect differences in peak AST, the small sample size did not allow a claim on the absence of an effect.

## Conclusions

In this RCT, we did not find evidence that a combined drug approach targeting the post–cold storage graft and the recipient was sufficient to decrease IRI. Targeting the graft before and/or during preservation may also be necessary. The advent of dynamic preservation may bring the opportunity to combine a downstream CDA in the recipient with upstream conditioning of the donor liver.

## Notes

Supplement 1.
Trial Protocol

Supplement 2. **eTable 1.** Surgical Complications **eTable 2.** Adverse Events and Serious Adverse Events

Supplement 3.
Data Sharing Statement
