# Supplementary material for: Effect of a Combined Drug Approach on the Severity of Ischemia-Reperfusion Injury During Liver Transplant: A Randomized Clinical Trial
Source: JAMA Netw Open. 2023 Feb 28;6(2):e230819. doi: 10.1001/jamanetworkopen.2023.0819 (PMC9975910; doi:10.1001/jamanetworkopen.2023.0819)
Supplement: Supplement 1. — Trial Protocol [file jamanetwopen-e230819-s001.pdf]

COMBINED DRUGS APPROACH TO PREVENT ISCHEMIA-REPERFUSION INJURY DURING TRANSPLANTATION OF LIVERS (CAPITL): a randomized clinical trial

Meurisse Nicolas, Mertens Markoen, Fieuws Steffen, Jochmans Ina, Sainz-Barriga Mauricio, Pirenne Jacques, Monbaliu Diethard.

Abdominal Transplant Surgery, UZ Leuven

Herestraat 49

3000 Leuven

Principal Investigator: Diethard Monbaliu

Tel: +32 16 348727

Fax: +32 16 348743

CONFIDENTIAL

Table of Contents

I. TITLE ... *7*

II. SUMMARY OF THE TRIAL ... *8*

III. RATIONALE AND NOVELTY ... *24*

3.1 RATIONALE ... **24**
3.1.1 Liver transplantation and the quest to solve the donor organ shortage ... 24
3.1.2 Ischemia reperfusion injury in LTx and graft function ... 25
3.1.3. Therapeutic strategies to attenuate ischemia reperfusion injury ... 28
3.1.4. Ischemia reperfusion injury leading to graft failure in a preclinical model of LTx at the KU Leuven ... 29
3.1.5. Outcome after LTx in our porcine model using our multifactorial pharmacological modulation protocol ... 30

3.2 NOVELTY ... **31**

3.3 FROM PRECLINICAL MODEL TO CLINICAL CAPITL STUDY ... **32**

3.4 DESIGN ... **36**

3.5 OBJECTIVES ... **39**

IV. PARTICIPANTS ... *40*

4.1 ELIGIBILITY CRITERIA ... **40**

4.2 INCLUSION CRITERIA ... **40**

4.3 EXCLUSION CRITERIA ... **40**

V. INTERVENTION ... *42*

5.1 CONTROL GROUP: Standard care of treatment ... **42**
5.1.1 Surgical procedure ... 42
5.1.2 Anaesthesia ... 42
5.1.3 Post-operative care in the intensive care unit ... 43
5.1.4 Post-operative follow up on nursing ward ... 44
5.1.5 Outpatient follow-up ... 44
5.1.6 Immunosuppression ... 45

5.2 TREATMENT GROUP: multifactorial modulation ... **47**

VI. TIME LINE & PARTICIPATING CENTER ... *49*

6.1 TIME LINE (Figure 3) ... **49**

6.2 PARTICIPATING CENTER ... **50**

VII. PRIMARY ENDPOINT (part B, RCT) ... *51*

VIII. SECONDARY ENDPOINTS (part B, RCT) ... *52*

IX. VARIABLES OF INTEREST ... *54*

9.1 DONOR CHARACTERISTICS ... **54**

9.2 RECIPIENT CHARACTERISTICS ... **54**

9.3 INTRA-OPERATIVE VARIABLES ... **54**

9.4 POST-OPERATIVE VARIABLES ... **55**

9.5 LABORATORY ASSESSMENT ... **55**

CONFIDENTIAL

9.6 HISTOLOGICAL FEATURES 55
X. DATA ANALYSIS 56
10.1 REPORTING OF THE DATA (part B, RCT) 56
10.2 STATISTICAL METHODOLOGY 56
10.2.1 Primary outcome 56
10.2.2 Sample size calculation 56
10.2.3 Secondary outcomes 57
XI. RANDOMIZATION (part B, RCT) 58
XII. ALLOCATION CONCEALMENT 58
XIII. ETHICAL CONSIDERATION 58
13.1 ETHICAL COMMITTEE 59
13.2 PATIENT INFORMATION AND INFORMED CONSENT 59
13.3 PERSONAL DATA PROTECTION 59
XIV. WITHDRAWALS AND DROPOUTS 60
XV. INTERIM ANALYSIS 61
XVI. PROCESS LEADING TO TRIAL STOPPING RULES 62
16.1 DATA SAFETY MONITORING BOARD 62
16.2 DESCRIPTION OF ADVERSE EVENT 62
16.2.1 Definition of adverse event (AE) and serious adverse event (SAE) 62
16.2.2 Grade of severity 62
16.2.3 Timeframe of observation 63
16.2.4 Predefined serious adverse events 63
16.4 DRUG CAUSALITY 64
16.5 INDIVIDUAL AND COHORT LEVELS 64
XVII. REFERENCES 66
XVIII. APPENDICES 68
APPENDIX 1: BANFF CRITERIA 68
APPENDIX 2: ADVERSE EVENTS 69
1. Definition of adverse events 69
2. Detection, reporting, and responsibilities 70
3. Surgical complication classification (within 30 days after transplantation) 71
4. Infection 72
5. Predefined types of adverse events 73
APPENDIX 3: COMBINED DRUG APPROACH 74
APPENDIX 4: SCHEDULE OF EVENTS 112
APPENDIX 5: ETHICAL PRINCIPLES FOR MEDICAL RESEARCH INVOLVING HUMAN SUBJECTS ... 113
APPENDIX 6: INFORMED CONSENT 117
APPENDIX 7: PATIENT INFORMATION BROCHURE: Part A. Safety study 125
APPENDIX 8: PATIENT INFORMATION BROCHURE: Part B. Randomized Controlled Trial 138
APPENDIX 9: CHRONOLOGICAL IDENTIFICATION LIST 158

CONFIDENTIAL

APPENDIX 10: CHRONOLOGICAL SCREENING LIST 159
APPENDIX 11: PARTICIPATING CENTERS 160
APPENDIX 12: SIGNATURE PAGE 161

CONFIDENTIAL

List of abbreviations

AE: Adverse event

ALT: Alanine aminotransferase

AR: Acute rejection

AST: Aspartate aminotransferase

cAMP: cyclic Adenosyl Mono-Phosphate

C1-INH: C1-inhibitor

CIT: cold ischemia time

DCD: Donation after circulatory death

DDI: Drug-to-drug interaction

DSMB: Data Safety Monitoring Board

ECD: Extended criteria donor

E-CRF: Electronic case report form

EPO: erytrhopoietin

ERCP: Endoscopic retrograde cholangio-pancreatography

GCP: Good clinical practice

GSH: glutathione

HCV: Hepatitis C virus

IRI: Ischemia-reperfusion injury

IV: Intravenous

LTx: Liver transplantation

MRCP: Magnetic resonance cholangio-pancreatography

NTBI: non-transferrin-bound iron

CONFIDENTIAL

PNF: primary non function

PTC: Percutaneous transhepatic cholangiography

RCT: Randomized control trial

ROS: Reactive Oxygen species

SAE: Serious adverse event

SCD: Standard criteria donor

SSAR: Suspected serious adverse reaction

SUSAR: Suspected unexpected serious adverse reaction

SSPP: Stable solution of plasma protein

TNF-α: tumor necrosis factor- alpha

CONFIDENTIAL

I. TITLE

Combined Drug **A**pproach to **P**revent **I**schemia-reperfusion injury during **T**ransplantation of **L**ivers (CAPITL study), a randomized clinical trial.

CONFIDENTIAL

II. SUMMARY OF THE TRIAL

TITLE

Combined Drug **A**pproach to **P**revent **I**schemia-reperfusion injury during **T**ransplantation of **L**ivers (CAPITL): a randomized clinical trial.

TRIAL DESIGN

A two-part, investigator driven, adaptive study to assess the safety and to study the efficacy of a Combined drug Approach to Prevent Ischemia-reperfusion injury during Transplantation of Livers.

The first part of the study (part A) was a safety study conducted in 10 patients undergoing a liver transplantation and was regarded to be safe by the Data and Safety Monitoring Board. These results will be submitted to the local ethical committee.

The second part of the study (part B) foresees in a randomized controlled trial conducted in patients undergoing a liver transplantation at the University Hospitals Leuven, Belgium.

Study part A: safety study

A phase I, **safety study** will be first performed (Figure 1) in 10 patients in 4 smaller cohorts. The rationale is to establish the safety of the combined drug approach aimed to reduce ischemia-reperfusion injury during liver transplantation in eligible recipients. The combined drug approach, also referred as multifactorial modulation, foresees the *sequential* administration of drugs in eligible liver transplant recipients. Drugs will be administered as such that they will *never be in direct contact with each other.*

There will be 10 patients included in Part A, conducted in 4 consecutive small cohorts of 2, 2, 3, and 3 patients, respectively (Figure 1). For this safety study, all consecutive patients listed for LTx will be assessed for study eligibility by a senior staff physician at time of organ offer. The data and safety assessment of each cohort will then be reviewed by the Data and Safety Monitoring Board (DSMB) before patients are included in the next cohort (see section 16.1).

Safety will be assessed by well-defined objective criteria and parameters based on incidence and/or severity of adverse events indicating a potential health hazard caused by the multifactorial modulation within 7 days post LTx (see chapter XVI: Process leading to trial stopping rules). When serious adverse events are observed with probable causality linked to one of the components, then a withdrawal of the incriminated component(s) will be investigated and implemented by the DSMB if possible.

CONFIDENTIAL

A positive feedback from the DSMB will allow to include the subsequent cohort of patients in the safety study. On the other hand, according the harshness, the recurrence and the limitation of acceptable side-effect rates of severe adverse events, the DSMB can stop the trial (cf. chapter XVI: Process leading to trial stopping rules).

The period of time (7 days) has been determined keeping in mind the half-life and pharmacokinetic data of the different components in order to have sufficient time to observe potential adverse events. As shown in table 1, the half-life of Anti-thrombin III, C1-inhibitor, erythropoietin-beta, melatonin, glutathione and alpha-tocopherol does not exceed 3 days. The half-life of infliximab is 14 days but the most important potentially expected side effect is an acute allergic reaction within the first hours following administration. No pharmacokinetic data are available for apotransferrin but no side effects were observed in clinical trials at the dose proposed in the combined drug approach. Concerning epoprostenol, an ex-vivo administration is foreseen through the portal vein during the surgical preparation of the graft, as such, no systemic absorption is anticipated.
During the safety study, data on the pharmacokinetics of the orally administrated components will be obtained as requested by the local Ethical Committee. PK profiles will be determined based on measurements in blood samples obtained after anesthesia induction, just before skin incision, immediately prior to the start of the anhepatic phase, immediately prior to reperfusion (defined as opening of the portal vein), 30 minutes, 1 h, 2 h, 6h, 12h, 24h, 48h, 72h after reperfusion, daily from day 4 to day 7, as foreseen by the protocol (see section 9.5). These measurements will be done in the Laboratorium voor Farmacotechnologie en Biofarmacie, KU Leuven, O&N2, Campus Gasthuisberg, Leuven. When the anticipated levels are not reached, a dose change can be done for the next cohort.

At the end of Part A (safety study), the peak of Aspartate aminotransferase (peak AST) of all 10 patients will be also assessed by senior staff physicians and the DSMB, as requested by the local Ethical Committee. Only in case of a major increase compared to a historical control group, peak AST in the safety study will be then compared with patients from a historical matched control group. For this purpose, patients will be matched by the following list of variables: donor age (20-30, 31-40, 41-50, 51-60, 61-70, 71-80, 81-90), donor type [donation after brain death (DBD), donation after circulatory death (DCD)], cold ischemia time (CIT) (4-6h, 6-8h, 8-10h, 10-12h, 12-14h), Lab MELD score (6-19; 20-24; 25-29; 30-34; >35), cause of death (trauma vs. non-trauma). The matching will be based on a propensity score that is the probability to be a patient in the safety study based on the aforementioned list of variables. Each patient in the safety study will be matched with one or more patients from the control group having a similar propensity score, i.e. 1:N case-control match on the propensity score will be performed.

All data and other information of each patient included in the safety study will be recorded in an electronic Case Report Form (e-CRF). The e-CRF will be designed to allow continuous recording of variables during the study. The e-CRF and its maintenance are technically organized by an external company (EONIX, Mons,

CONFIDENTIAL

Belgium www.eonix.be). The e-CRF will be completed under the responsibility of the principal investigator.

At the end of Part A (safety study), the results (including pharmacokinetic profile for melatonin and vitamin E as well as peak AST results) will be submitted to the Ethical Committee and approval for part B (randomized controlled trial) will be requested if appropriate.

CONFIDENTIAL

Study part B: randomized controlled trial

Following approval by Ethical Committee, the second phase of the trial will be continued as an investigator driven, phase III, randomized study.

For this RCT, all consecutive patients listed for LTx at the University Hospitals Leuven, Belgium will be assessed for study eligibility by a senior staff physician at time of organ offer. Patients will be randomly assigned to the treatment group (multifactorial modulation) or control group (standard of care treatment alone).

Eligible patients will be randomized at time of organ offer according to computer-based varying block size-generated randomization list by a third, independent party (EONIX).

CONFIDENTIAL

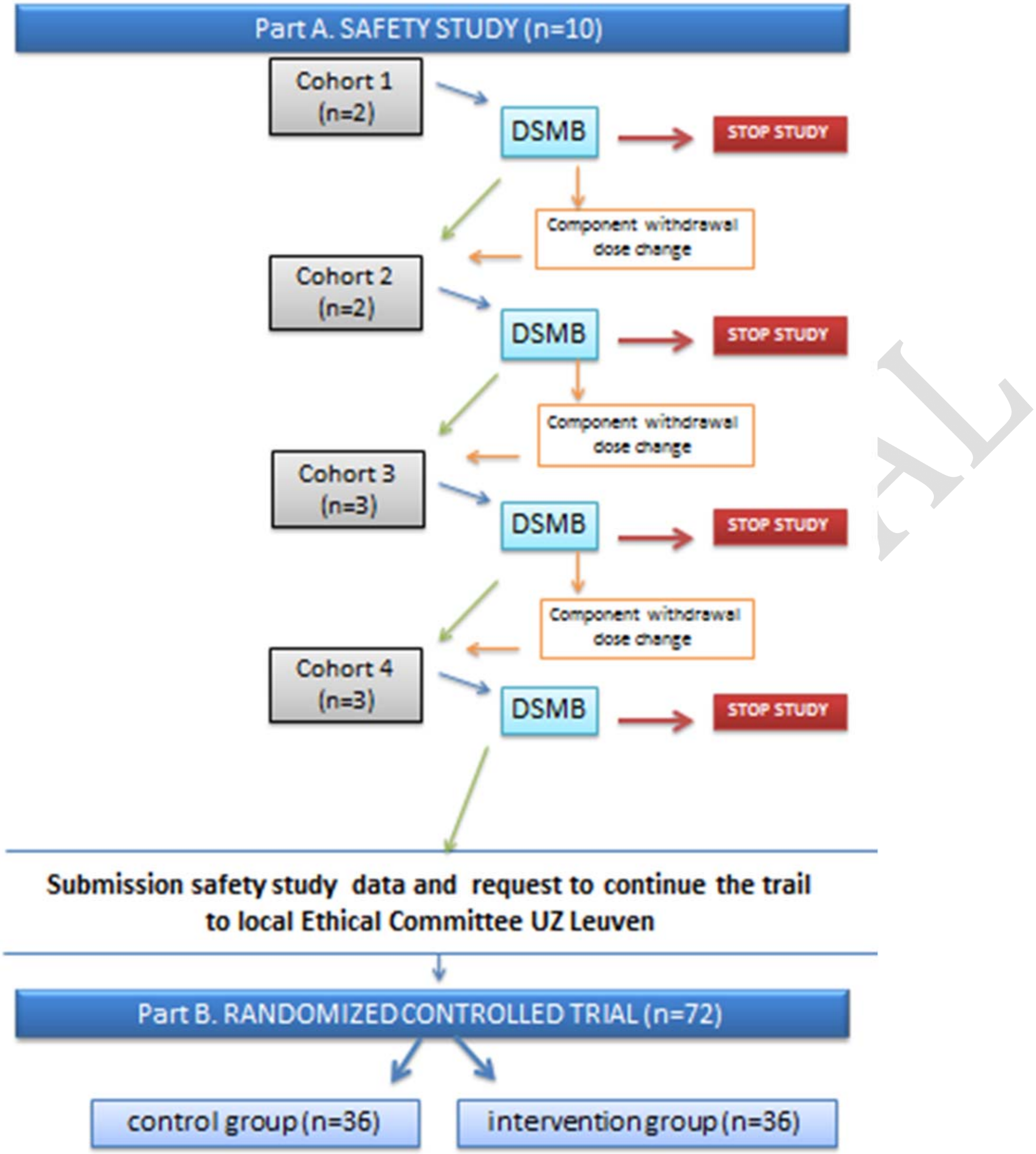

Figure 1. Description of the CAPITL study. First a phase I, safety study will be initiated. After each cohort of patients, the Data Safety Monitoring Board (DSMB) will analyze the data with a particular attention for safety. Hereafter, there are three possibilities: (i) the study is considered to be safe and the study can continue with the following cohort (green arrow), (ii) When serious adverse events have been observed with causality linked to one of the components, then a withdrawal of the incriminated component(s) will be investigated and implemented by the DSMB if possible (orange arrow), or (iii) the study is considered not to be safe and will be stopped (red arrow). After the first safety study, data will be submitted to the local Ethical committee and approval to continue the study requested. Following approval of the local Ethical committee to continue the second phase of the trial eligible patients will be randomly assigned to the treatment group (multifactorial modulation) or control group (standard of care treatment alone).

OBJECTIVE

To demonstrate
- the safety of the multifactorial modulation (part A)
- the effectiveness of the multifactorial modulation in reducing the peak AST – a surrogate marker of ischemia-reperfusion injury (IRI) - after LTx (part B).

PRIMARY ENDPOINT (part B: RCT)

The primary endpoint of the RCT is the log-transformed peak AST, where peak AST is defined as the highest value of serum AST within 72 hours following LTx.

PLANNED NUMBER OF PATIENTS (part B: RCT)

For the RCT, 36 patients are planned to be included in each group. This number is based on a sample size calculation using peak AST values obtained from a historical series of patients that underwent LTx at the University Hospitals Leuven.

SECONDARY ENDPOINTS (part B: RCT)

- *Graft loss at 3, 12 months after LTx.* Graft loss is defined as the need for retransplantation within one week post LTx due to a non-life-sustaining liver graft function (PNF) or later (other reason).

- *Recipient death* at 3, 12 months after LTx.

- *Early graft dysfunction* as defined by Olthoff (1): the presence of one or more of the following postoperative laboratory analyses: bilirubin ≥ 10mg/dL on day 7, international normalized ratio ≥ 1.6 on day 7, and alanine aminotransferase (ALT) or AST > 2000 IU/L within the first 7 days.

- *Incidence of biliary strictures within 12 months post LTx:* a biliary stricture is defined as a narrowing within the biliary tree, radiologically evident [endoscopic retrograde cholangio-pancreatography (ERCP) and/or magnetic resonance cholangiopancreatography (MRCP)] to cause clinical symptoms or biochemical abnormalities requiring intervention (ERCP, percutanous transhepatic cholangiographic (PTC) drainage, surgery, retransplantation). Biliary strictures are categorized as anastomotic or non-anastomotic based on the cholangiographic appearance of the biliary tree as judged by a blinded radiologist. Non-anastomotic strictures are defined as any strictures, dilatation, or irregularity of the intra- or extrahepatic bile ducts of the liver graft at a site(s) other than that of the anastomosis. Intra-hepatic biliary strictures are classified in 4 groups: unilateral focal, confluence, bilateral multifocal and diffuse necrosis (2). Beside the routine 1 year post-transplant assessment of the biliary tree by MRCP and/or ERCP, biliary strictures will be investigated in case of clinical or biochemical suspicion (based upon cholestasis). Other causes leading to

CONFIDENTIAL

cholestasis (e.g. hepatic artery thrombosis, bile leakage, rejection or cholangitis) will be excluded based on state –of-the-art radiological and histological examination as part of the routine standard treatment of care. These examinations include ultrasound Doppler, CT and CT angiogram, biopsy-proven rejection based on histology scored by 2 blinded experienced liver pathologists according to the Banff criteria, ERCP and MRCP.

- *IRI score*: The extent of IRI will be assessed by a histological score based on the degree of cytoplasmic vacuolization, sinusoidal congestion, necrosis of parenchymal cells, apoptosis and influx of neutrophils as described by Suzuki score (3) and Monbaliu et al. (4). Liver biopsies will be taken before implantation (bench table), 1 hour after reperfusion and 1 week after transplantation and blindly scored by 2 pathologists.

- *Graft rejection*: a liver biopsy is taken after LTx at 1 week post-LT and in case of clinical suspicion of acute rejection. Clinical suspicion of acute rejection may be based on clinical symptoms (such as jaundice, low-grade fever) or sometimes nonspecific complaints (such as generalized malaise, decreased appetite) and/or biochemical abnormalities (usually increasing or plateauing levels -in an abnormal elevated range- of liver tests that were returning to normal values). Histological changes will be scored blindly according the BANFF criteria (see appendix 1) by 2 experienced liver pathologists.

- *Severe surgical complications*: the Clavien-Dindo classification (5) will be used to rank severe surgical complications (≥ 3b)within 30 days after LTx according to an objective, simple, reliable, and reproducible manner. This classification is based on the therapy required to treat the complication (appendix 2, adverse event). The severity of the biliary strictures as well will be classified using this standardized grading system.

- *Acute Kidney Injury:* The rate of AKI is assessed by the Risk, Injury, Failure, Loss, and End-Stage kidney disease (RIFLE) criteria at 48 hours after graft reperfusion and defined by the fold change in serum creatinine from baseline (after anaesthesia induction) (14, 28). The severity of AKI was defined by the largest creatinine change observed, and classified as follows: <1.5 fold change compared with baseline was defined as normal renal function, >1.5 but <2 as acute renal risk (AKI-R), >2 but <3 as acute renal injury (AKI-I), and >3 or need for renal replacement therapy as acute renal failure (AKI-F).

- *Post-reperfusion syndrome:* The rate of post-reperfusion syndrome was defined as a 30% decrease of mean systemic blood pressure for more than 1 minute during the first 5 minutes following graft reperfusion (31).

CONFIDENTIAL

ELIGIBILITY

- Patient suffering from irreversible liver failure eligible for LTx according to Eurotransplant guidelines.
- Patients ≥ 18 years of age at time of listing on the waiting list for LTx at the University Hospitals Leuven, Belgium.

All consecutive patients listed for LTx will be assessed for study eligibility by a senior staff physician via the outpatient clinics (pre-transplant evaluation) or as an inpatient during their hospital stay. If patients are willing to participate to this multi-center randomized controlled trial and provide informed consent, a member of the LTx team will inform patients about the study, orally and in writing.

EXCLUSION CRITERIA

- Patients who refuse to participate in the study,
- Patients suffering from acute liver failure
- History of hypersensitivity to anti-thrombin III (Atenativ®), C1-inhibitor (Cetor®/Cinryze®), melatonin (Circadin®), epoprostenol (Flolan®), recombinant human EPO (Neorecormon®), infliximab (Remicade®), glutathione (Tationil®), tocopferol (vitamin e suspension 100 mg/mL®) will be excluded from the treatment group (cfr. Appendix 3).
- Conditions that prevent the use of the multifactorial modulation (cf. chapter XVIII, Appendix 3.2):
    - Administration of heparin at therapeutic dose pre-operatively: anti-thrombin III (Atenativ®), epoprostenol (Flolan®).
    - Congestive heart failure arising from severe left ventricular dysfunction: epoprostenol (Flolan®), infliximab (Remicade®).
    - History of seizures (not related to the underlying liver disease or to metabolic disturbances secondary to liver cirrhoris and to be distuinghuised from e.g. hepatic encephalopathy), poorly controlled arterial hypertension, myocardial infarction or stroke in the month preceding the liver transplantation, and history of pre-existing venous thromboembolic disease which is not related to liver cirrhosis and hypercoagulability (eg. partial, complete or previous vena porta thrombosis/ vena mesenterica thrombosis/ vena lienalis): recombinant human EPO (Neorecormon®).
    - Unstable angina pectoris: recombinant human EPO (Neorecormon®).
    - Severe untreated infections such as sepsis, abscesses and opportunistic infections: infliximab (Remicade®).
    - Use of Vitamin K antagonist anticoagulation preoperatively which can not be reversed, women taking oral contraceptives containing oestrogens: tocopferol (vitamin E suspension 100 mg/mL®).
    - Patients with previous treatment of infliximab (Remicade®),

CONFIDENTIAL

- Mental conditions rendering the subject incapable to understand the nature, scope, and consequences of the trial,
- Combined organ transplantation,
- Re-transplantation,
- Patients that are dialysis-dependent prior to LTx,
- LTx from a living or a split organ donation
- Administration of the multifactorial modulation technically non-feasible (i.e impossibility to place the second central catheter required for the *separate* and *sequential* injection of the different components of the multifactorial modulation).

CONFIDENTIAL

TREATMENT

Apart from receiving the standard care as described below (cfr Chapter control group: Standard care of treatment), patients randomized to the treatment group receive:

1. C1-inhibitor
2. α-Tocopherol
3. Glutathione
4. Apotransferrin
5. Human Recombinant Erythropoietin beta (EPO-β)
6. Infliximab
7. Antithrombin-III
8. Epoprostenol
9. Melatonin

Detailed description on administration, dosing and treatment periods can be found in Table 1 and Figure 2.

Briefly, C1-inhibitor, Glutathione, Apotransferrin, Human Recombinant Erythropoietin beta (EPO-β), Infliximab and Antithrombin-III will be infused intravenously (IV) **sequentially** and through different perfusion sets **without direct contact** during the anhepatic and the reperfusion phase. A second infusion of EPO-β is foreseen 6 hours after reperfusion. This IV sequential administration just before reperfusion aims to reach a plasmatic peak of concentration of each component directly after their infusions. As recommended by independent feedback of pharmacologist experts Prof Pieter Annaert (Laboratorium of Pharmacotechnology and Biopharmacy, KU Leuven) and Prof Paul Declerck , (Laboratorium of Pharmaceutical biology, KU Leuven) (Cfr Addendum Feedback regarding CAPITL study) the infusion of infliximab is interrupted during the administration of Glutathione. Melatonin and α-Tocopherol will be given orally before the transplantation. According to the available pharmacokinetic data, plasmatic peaks of concentration should be reached after reperfusion. This will be evaluated during the safety study where the pharmacokinetics of melatonin and α-Tocopherol will studied. Epoprostenol will be added to 1L of University of Wisconsin preservation solution and infused ex-situ directly into the liver through the vena portae during the bench table before the implantation. To avoid any contact between the components, a short flush of isotonic electrolyte solution will take place between the sequential infusions of the components. The infusion of Remicade® (anti-TNF-alpha antibody) starts before the reperfusion, is interrupted during administration of glutathione and then restarted 15 minutes after reperfusion (period of time required to stabilize the patient after the reperfusion). The infusion of apotransferrine (scavenger of non-transferrin-bound iron) starts before the reperfusion, is interrupted for the administration of EPO-β, C1-inhibitor and glutathione, and then restarted 15 minutes after reperfusion. As such, the manufacture's guidelines are followed (slow infusion rate for infliximab (Remicade ®) and larger volume for apotransferrine, respectively). Moreover, as our preclinical studies have shown: the peak of TNF-α and non-transferrin bound iron was observed 3 hours and 1 hour after the reperfusion, respectively.

Except for Apotransferrin, all the components are registered and described in the MICROMEDEX Healthcare Series (6) and in the MARTINDALE (7).

CONFIDENTIAL

Except for apotransferrin, the administration of a single dose of each drug component, at the dose described in Table 1, has been proven to be safe and efficient in reducing IRI in both animals and clinical studies. A full description of current uses, precautions and contraindications, proposed mechanism of action, expected adverse effects, drug interactions, dosage form, and packaging can be found in appendix 3.

CONFIDENTIAL

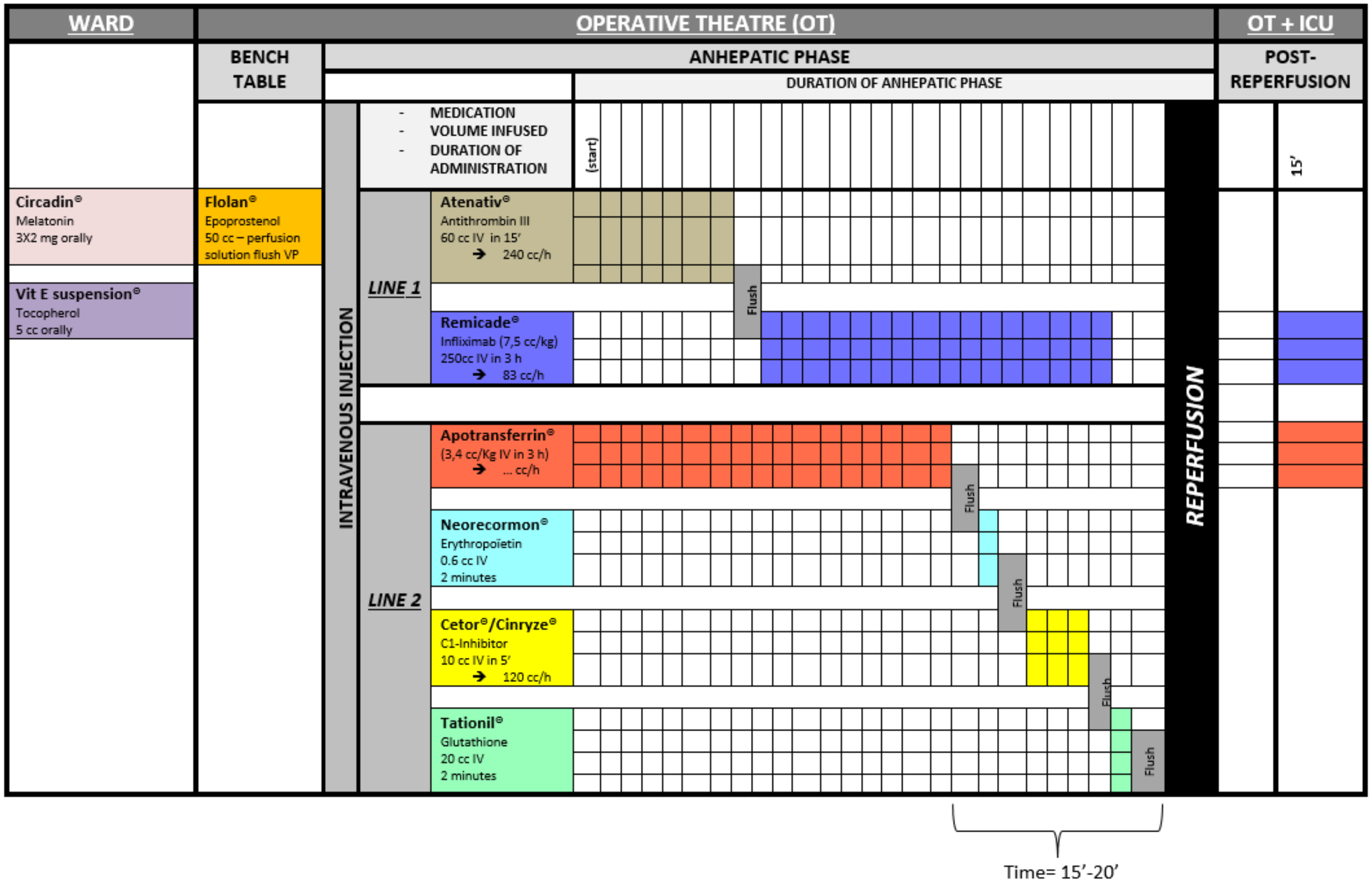

Figure 2. Schematic overview of the combined drug approach: sequence and way of administration of the components of the multifactorial modulation during the different phases of the LTx in the treatment arm. A short flush of isotonic electrolyte solution will take place between the sequential infusions of the components in order to avoid any contact between components.

Product (Company)	*Mode of action*	*Half-life*	*Dose*	*Total volume required for reconstitution*	*Way and duration of administration*	*Timing of administration*	*Reported side effects at recommended dose*	*Main described side effects in literature (Cfr appendix 3)*
**Antithrombin III Atenativ®** (Octapharma)	- Increase the release of prostacyclin and NO.<br>- Reduction of inflammatory cell migration.<br>- Decrease the formation of ROS.	72 hours	3000 IU	60 cc	IV – 15 minutes	Start of anhepatic phase	No	Allergic reactions, arterial hypertension
**C1-inhibitor Cetor®/Cinryze®** (Sanquin/ViroPhar-ma (taken over by Shire))	- Inhibition of classical (+++), lectine (+) and alternative (+) pathway of the complement activation<br>- Regulation of intrinsic and fibrinolytic pathways of the coagulation cascade.<br>- Anti-inflammatory protein	42 hours	1000 U	10 cc	IV – 5 minutes	10 minutes before reperfusion	No	Allergic reactions, Anaphylactic shock
**EPO-β Neorecormon®** (Roche)	- Anti-apoptotic.<br>- Reduction of inflammatory cytokines.<br>- Antioxidant.	12 hours	30.000 IU<br>+<br>30.000 IU	0.6 cc<br>+<br>0.6 cc	IV – 2 minutes	13-15 minutes before reperfusion<br>+<br>6 hours after reperfusion	No	Thrombus, seizure, arterial hypertension
**Melatonin Circadin®** (Nycomed)	- Antioxidant.<br>- ROS scavenger.	4 hours	6 mg	3 capsules	Orally	On the ward before the transplantation	No	No
**Epoprostenol Flolan®** (GlaxoSmithKline)	- Vasodilatation.<br>- Antioxidant.<br>- Inhibition of platelet aggregation.<br>- Reduction of leukocyte activation and adhesion.	0.1 hour	500 µg	50 cc	Flush through the vena porta during the bench table	Ex-situ during the bench table before the implantation	Ex-situ administration (no foreseen systemic absorption)	In case of systemic absorption: headache, hypotension, arrhythmia, heart failure,

**Glutathione Tationil 600®** (Roche Italy)	- Antioxidant.<br>- ROS scavenger.	0.25 hour	3 g	20 cc	IV – 2 minutes	2-4 minutes before reperfusion	No	No
**Infliximab Remicade®** (Janssen Biologics)	- Inhibition of inflammatory cascade by blocking both soluble and transmembrane forms of Tumor Necrosis Factor-α.	14 days	3 mg/Kg	7.5 cc/Kg	IV – 3 hours	Start of anhepatic phase after infusion of Antihrombin III<br><br>Interruption of the infusion during the administration of Glutathione<br><br>Restarted 15 minutes after reperfusion	Anaphylactic reaction first hours following administration	Anaphylactic shock, hematological effect
**Vitamin E suspension** 100 mg/ml (Cambridge Laboratories)	- Antioxidant.<br>- ROS scavenger.<br>- Increase the release of prostacyclin.	53 hours	500 mg	5 cc	Orally	On the ward before the transplantation	No	No
**Apotransferrin** (Sanquin)	- Non-transferrine bound iron (redox active) chelator.	/	170 mg/kg	3,4 cc/Kg	IV – 3 hours	Start of anhepatic phase<br><br>Interruption for sequential administration of erythropoietin, C1-inhibitor and Glutathione<br><br>Restarted 15 minutes after reperfusion	No	No

Table 1: List of components of the multifactorial modulation. For each component, the mode of action, the half-life, the dose, the total volume required for reconstitution, the way of administration, the timing of use and described side effects are described. Reperfusion is defined as restoration of the hepatic inflow through the portal or the hepatic artery or by both.

CONFIDENTIAL

TIME LINE

- **2012:**
    - Finalising multi-factorial modulation protocol,
- **2013:**
    - Finalising multi-factorial modulation protocol
    - Start recruitment of patients,
    - Start inclusion of patients in the first safety phase of the study,
    - Start inclusion of patients in RCT phase of the study,
    - Start data analysis of patients included in safety phase study,
    - Inclusion of patients in the study if safety study is considered as safe,
    - Start data collection,
- **2014:**
    - Recruitment of patients in RCT phase continued,
    - Data collection continued.
- **2015:**
    - Recruitment of patients in RCT phase continued,
    - Data collection continued.
- **2016:**
    - Recruitment of patients in RCT phase continued,
    - Data collection continued.
- **2017:**
    - Data collection continued,
    - Start data analysis.
- **2018:**
    - Data analysis continued.

CONFIDENTIAL

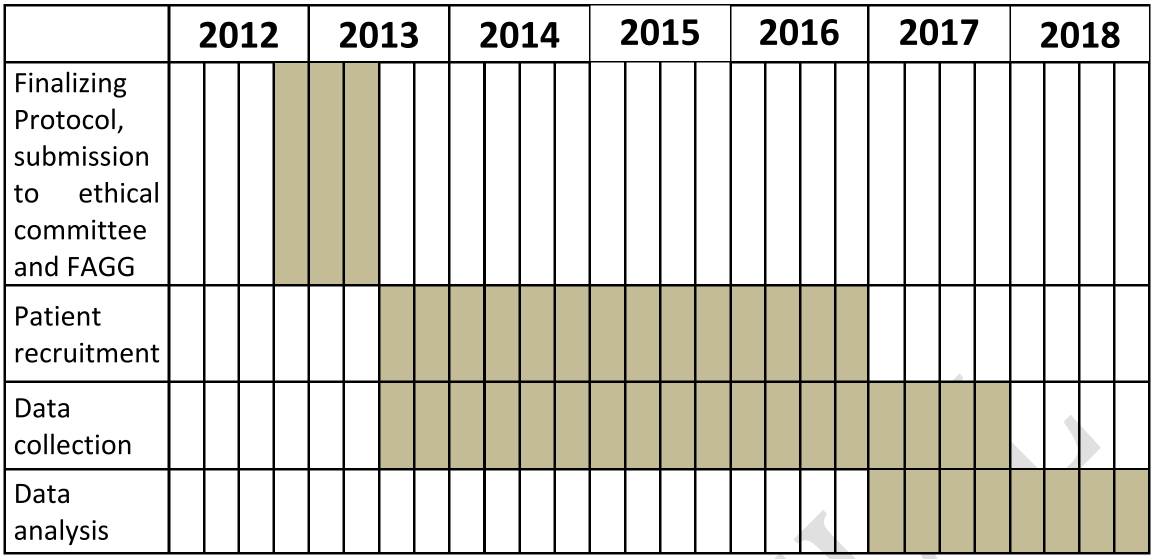

Figure 3 Time line of the RCT

CONFIDENTIAL

III. RATIONALE AND NOVELTY

3.1 RATIONALE

3.1.1 Liver transplantation and the quest to solve the donor organ shortage

LTx has become the treatment of choice for liver failure offering patients both an improved survival and quality of life. Indeed, excellent outcome after LTx with reported 1 and 5 years graft and recipient survival exceeding 90% and 75% are achieved in our institution. Furthermore, successful LTx allows recipients a return to a near-to-normal life style. Currently, the annual incidence of LTx is ~20 per one million inhabitants (~100-120 LTxs are performed every year in Flanders, ~1500 within the Eurotransplant area).
The success of LTx has resulted in a dramatic shortage of organs, resulting in death of patients on the waiting list. This justifies the search for novel strategies to prevent death of patients who could have benefitted a life-saving transplantation.

Liver grafts are usually retrieved from brain-dead donors, but due to the afore-mentioned worldwide shortage (8), there is an increasing interest by using less-than-optimal donors that offers the most immediate promise to substantially enlarge the donor pool for LTx. Those grafts -the so-called extended criteria donor grafts (ECD) - previously considered unsuitable for transplantation are increasingly used by many centers, including our center (9). Factors defining extended criteria liver grafts include donor age (>65 years), graft steatosis, prolonged cold ischemia time (>12 hours), cause of death, hemodynamic stability at the time of procurement, and donation after circulatory death (DCD).

Grafts from these donors are known to be more sensitive for IRI (10) which is the main cause of early graft dysfunction. Early graft dysfunction is clinically and biochemically characterized by liver injury and poor function (1). Most importantly, early graft dysfunction is associated with an increased risk of mortality and graft loss which jeopardizes the short and long term outcome for liver transplant recipients.

In addition, in search for objective and transparent allocation of these scarce donor organs, recipient allocation policies have been adopted and have brought sicker patients to the operation room. Indeed, prioritizing recipients for a LTx is based on the Model of End-stage Liver Disease (MELD) score. The MELD score was developed to predict survival and reflects severity of the end-stage liver disease based on the patient's values for serum bilirubin, serum creatinine, and the international normalized ratio for prothrombin time (INR). Since liver grafts are more often transplanted into sicker patients, this also affects graft and recipient survival.

This coincidence of donor and recipient factors contributing to early graft dysfunction justifies the strong need to investigate liver-protective strategies in LTx as well.

In this context, the commonly applied strategy of organ preservation using hypothermic preservation solutions no longer fulfils the demand to maintain graft viability after transplantation. Additional new strategies are needed to overcome this increased risk of early graft dysfunction, and should aim to tackle IRI.

CONFIDENTIAL

3.1.2 Ischemia reperfusion injury in LTx and graft function

In the course of LTx, IRI represents an important non-immunologic antigen-independent factor that influences graft outcome, increases graft immunogenicity and host-allo-responsiveness (11, 12, 13). Moreover, as recently assessed by our team, the severity of IRI as reflected by the peak AST>2000IU/l per se has a substantial impact on patient survival (figure 4).

IRI is characterized by a complex series of interrelated events which invariably takes place in *every* graft during reperfusion in the recipient (11, 12, 13, 14) (Figure 5 and 6).

Briefly, in the transplantation setting, organs become deprived from oxygen during procurement and preservation. Restoration of oxygenation at the moment of graft reperfusion with blood enhances the ischemic injury caused at a cellular level resulting in an inflammatory self-amplifying loop and hepatocellular damage. The extent of injury to the graft on reperfusion is variable from minimal over severe to total destruction of the graft (12, 13) and depends on the degree of activation of key players including Kupffer cells, platelets and leukocytes, besides the generated pro-inflammatory response (oxidative stress, inflammatory cytokines, cytoplasmatic proteases, up regulation of pro-inflammatory transcription factors…).

Clinically IRI can result in immediate graft function, early graft dysfunction (considered to occur in 10-30% of grafts) or primary graft non-function (considered to occur in < 5% of grafts), respectively (15). Furthermore, the biliary tree is especially vulnerable to IRI, contributing to intrahepatic biliary strictures, a common reason of late graft loss (16). Finally, IRI also contributes to graft rejection since it activates a cascade of innate-dominated pro-inflammatory immune responses culminating the adaptive immune response.

Because graft dysfunction affects both short and long term graft survival, understanding and attenuating hepatic IRI is regarded an imperative strategy to improve short and long term outcome of liver grafts, especially with the increased use of less than ideal liver grafts. Basic research using animal models has elucidated dominant molecular pathways important in the pathogenesis of liver IRI (Figure 4 and 5). This knowledge has resulted in designing different therapeutic modalities to reduce IRI.

CONFIDENTIAL

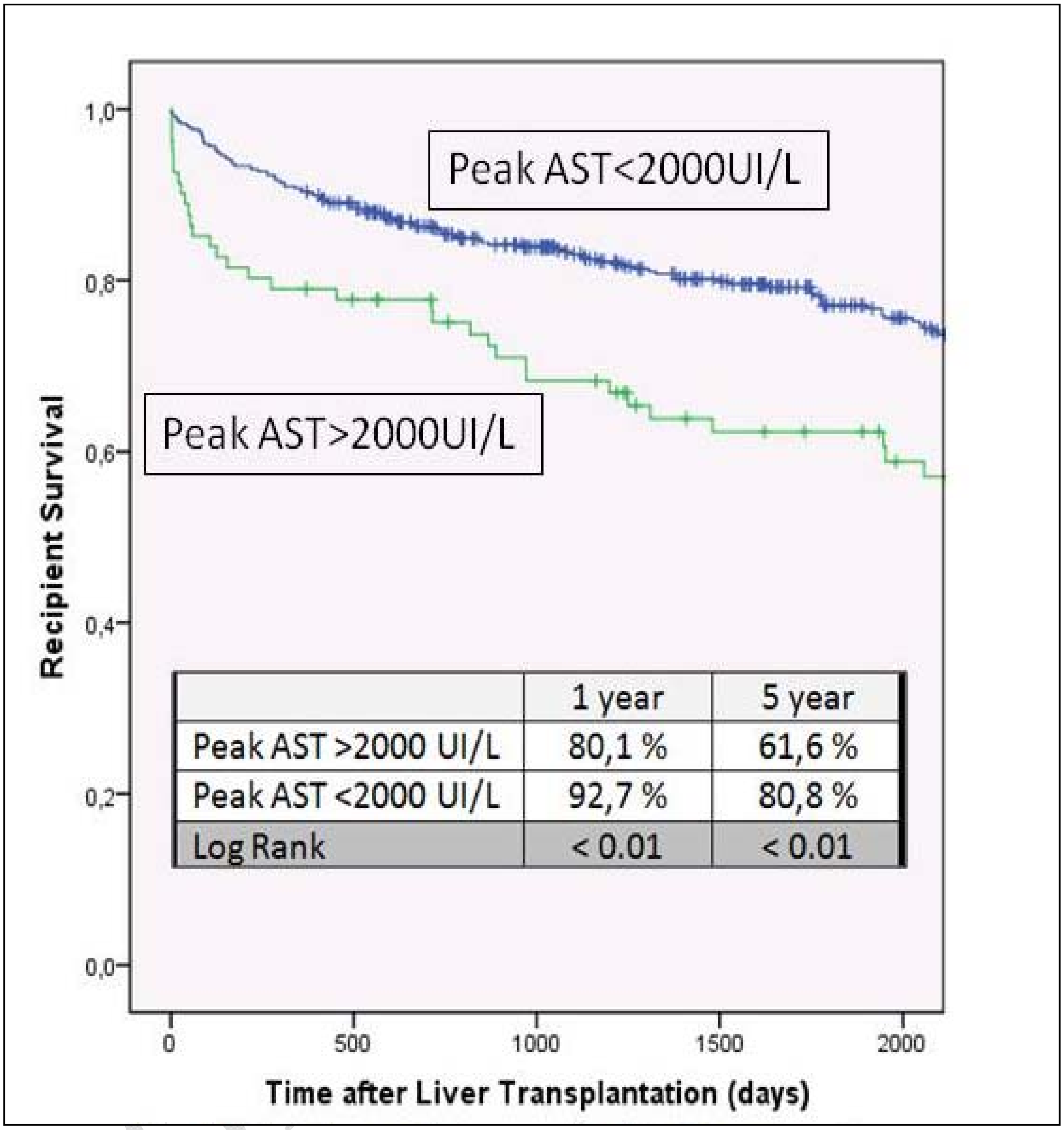

Figure 4: In a historical cohort of 552 LTx recipients at UZ Leuven (01/2000 – 12/2010) inferior recipient survival is observed in recipients with severe IRI (peak AST > 2000 IU/L) compared to recipients with a less severe IRI (peak AST < 2000 IU/L).

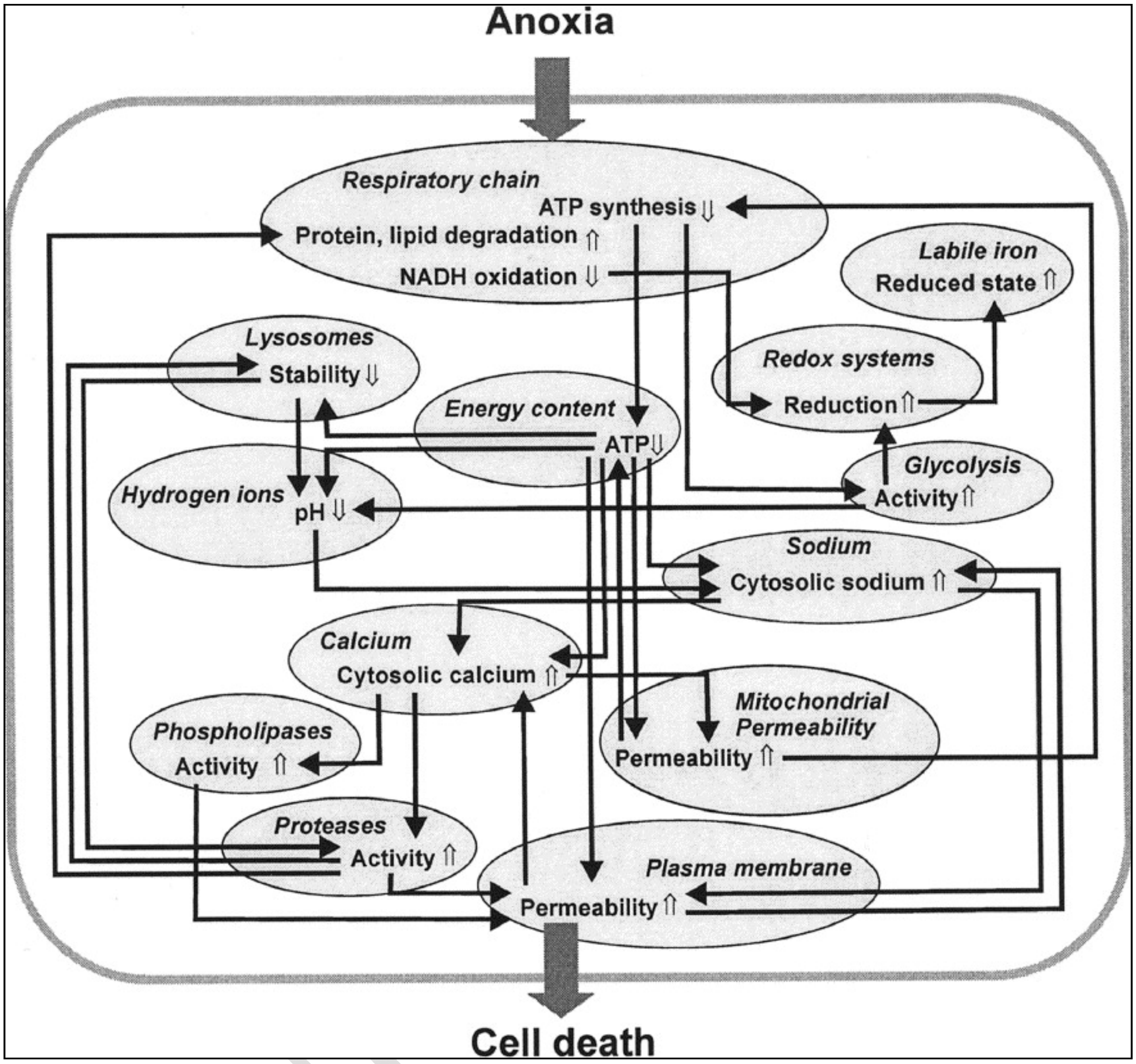

Figure 5: Schematic overview of the pathogenic network of anoxic cell resulting in self-amplifying loops of several injurious pathways (14).

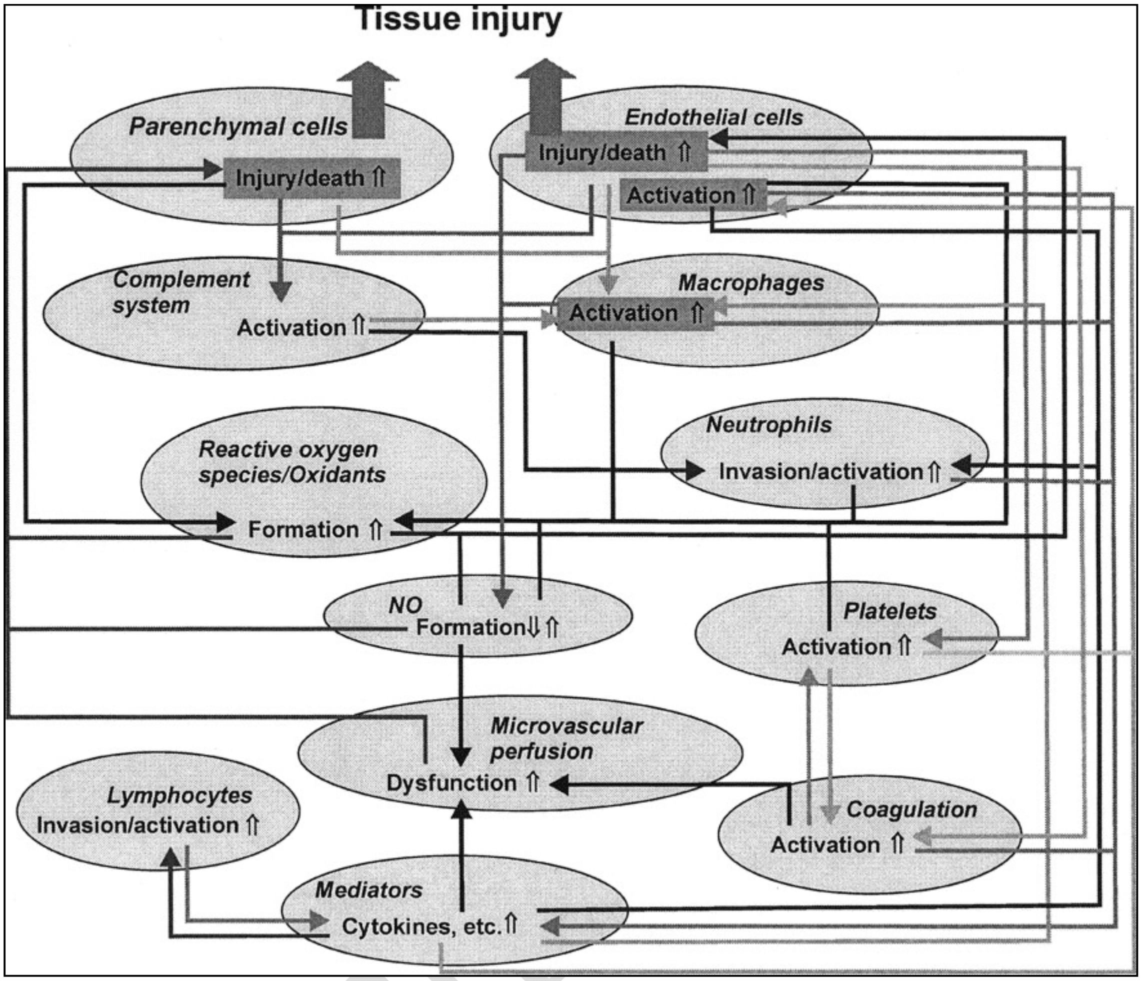

Figure 6: Pathogenic network of the inflammatory response of ischemia reperfusion injury with the initiation of self-amplifying loops of several injurious pathways (14).

3.1.3. Therapeutic strategies to attenuate ischemia reperfusion injury

To improve outcome of ECD and DCD LTx, and to increase their safer use, attenuating the IRI cascade by pharmacological modulation is an interesting concept. Several drugs have already been tested in animal models. However, only few strategies have been tested in humans and very few are currently implemented as common clinical practice (17, 18, 19, 20, 21, 22, 23).

As mentioned above, the IRI cascade is notorious for its redundant pathways (Figure 4 and 5). Hence, blocking one component of the cascade is rarely enough to decrease the severity of IRI. We therefore believe it is essential to combine pharmacological agents acting simultaneously at several steps of the IRI cascade referred to as combined drug approach.

3.1.4. Ischemia reperfusion injury leading to graft failure in a preclinical model of LTx at the KU Leuven

The KU Leuven group previously studied IRI in a large animal (pig) LTx model. More in particular, the IRI using livers from DCD was studied in this model inducing a well-characterized and controllable injury of normothermic ischemia prior to procurement, cooling, preservation and transplantation. Using such liver grafts has been suggested a promising strategy to extend the donor pool, but has been found associated with a poor graft survival (10–15% lower compared to the traditional brain death donors) because of a higher rate of primary graft non-function and intrahepatic bile duct strictures or ischemic-biliary lesions. We therefore first investigated the tolerance of livers exposed to warm ischemia, provided clinical guidelines to avoid transplantation of grafts destined to fail and elucidated some mechanisms contributing to this graft failure (24).
LTx in a porcine model is generally regarded to be clinically relevant because of the undeniable physiological and anatomical similarities between pigs and human. It is considered at the same time as an extremely stringent model. Because neither vasopressive drugs, nor transfusions of blood derivatives are used, the natural evolution of the hepatic injury and inflammation in such a model remains unmasked. We observed that – prior to a short fixed cold ischemic period - 15 min of warm ischemia was well-tolerated, whereas warm ischemia ≥ 30 min and ≥ 60 min, induced a 50 or 100% PNF risk, respectively (24).
In grafts destined to fail, warm ischemia on its own caused hepatocellular damage and activation of Kupffer cells prior to cold storage and reperfusion. In recipients with PNF, this resulted in an overproduction of inflammatory cytokines (in particular TNF-α and IL-6) and a failure of some antioxidant mechanisms (especially α-tocopherol and glutathione). In PNF recipients, high quantities of redox-active iron - known to catalyze the generation of free oxygen radicals - were released in blood (2). Moreover in PNF recipients, an increased activity and decreased inhibition of serum PhospholipaseA2 were observed (25). Furthermore, in this preclinical transplantation model, exposure to incremental periods of warm ischemia was also found related to increased bile salt toxicity after transplantation (24). Extending the cold ischemia - even following a short warm ischemia – caused graft dysfunction and inversely affected post-transplantation recipient survival (26).

Based on these pathophysiological IRI mechanisms contributing to graft failure, we designed a multifactorial modulation strategy targeting these mechanisms (Table 2)(4). We focused on (i) optimizing graft quality during procurement and preservation and (ii) administering multiple biological reagents expected to attenuate the IRI in the recipient. Most of the agents selected had to represent natural sources (glycine, glutathione, α-tocopherol, α1 acid glycoprotein, and apotransferrin) or known to be non-toxic under physiological circumstances/concentrations. In other words, it could be anticipated that these drugs can be safely infused. The use of these products had also to be supported by literature reports and available for clinical or preclinical use. Any intervention in the donor – prior to declaration of death – was omitted in this protocol to avoid all potential ethical conflicts when applying this strategy clinically at a later stage. This resulted in a multifactorial strategy combining several drugs and operating on different mechanisms and at different stages of the IRI cascade (table 2). In addition, we speculated that the cumulative/synergistic effect of this multifactorial modulation would exceed the effect of the individual components when administered in monotherapy.

CONFIDENTIAL

Active components	Mechanism
Glycine	Kupffer cell stabilizer, hepatocyte and endothelial cell protector
Gluthatione	Antioxidant
Apotransferrin	Redox-active iron chelator
α1-acid glycoprotein	Antioxidant
α-tocopherol	Antioxidant
FR167653	MAPK inhibitor

Table 2: components of the multifactorial modulation used in the preclinical model and their mechanisms of action.

3.1.5. Outcome after LTx in our porcine model using our multifactorial pharmacological modulation protocol

The working hypothesis was that in porcine livers exposed to long warm ischemia and normally destined to fail, such a multifactorial modulation strategy would reduce the **IRI** and that this would result in better liver function, and increased graft and recipient survival.

Prior to the design of the study, no power analysis or sample calculation was performed since this was a seminal study for which no prior data were available. Therefore - in retrospect- a one-tail power calculation with an alpha error level of 5% (DSS research, http://www.dssresearch.com/toolkit/spcalc/power_p2.asp) was performed using the described cohorts and the observed recipient and graft survivals which corresponded to a statistical power of 80.2% for graft and 80.5% for recipient survival, respectively.

Porcine livers exposed to 45 min warm ischemia were cold stored, transplanted and either modulated (n=6) or not (controls, n=9). In the modulation group, donor livers were flushed with warm Ringers (avoiding cold-induced vasoconstriction), streptokinase (eliminating stagnating thrombi) and epoprostenol (vasodilator, platelet aggregation inhibitor) prior to cold storage. In recipients, glycine (Kupffer cell stabilizer), α1-acid-glycoprotein (anti-inflammatory protein), MAPKinase-inhibitor (pro-inflammatory cytokine generation inhibitor), α-tocopherol and glutathione (anti-oxidants), and apotransferrin (iron chelator) were administrated intravenously. PNF, survival, lactate, transaminase, TNF-α, redox-active iron, and biliary bile salt-to-phospholipid ratio were monitored. No PNF was observed in modulated versus 55% in control pigs (p=0.025). Survival was 83% in modulated versus 22% in control pigs (p=0.02). At 180 min post-reperfusion, lactate was lower in modulated (5.4±1.9 mmol/L) versus control pigs (9.4±2.2 mmol/L; p=0.011). At 60 min post-reperfusion, there was a trend for lower AST in modulated versus control pigs at 60 min (939±578 versus 1683±873 IU/L; p=0.089). Post-reperfusion, TNF-α remained stable in modulated pigs (49±27pg/ml at 15 min and 85±26pg/ml at 180 min; p=0.399) but

CONFIDENTIAL

increased in control pigs (107±36pg/ml at 15 min and 499±216pg/ml at 180 min; p=0.023). At 180 min post-reperfusion, redox-active iron was lower in modulated pigs versus control pigs (0.21±0.18 versus 0.042±0.062 μM; p=0.038). Biliary bile salt-to-phospholipid ratio post-LTx was lower in modulated versus control pigs (1128±447 versus 4836±4619; p=0.05) (4).

In conclusion, our preclinical study demonstrated that in a stringent model of severe IRI (as in DCD LTx), a multifactorial modulation containing several biological reagents targeting previously identified mechanisms of warm IRI remarkably improved the degree of IRI, eliminated PNF, reduced TNF-α, improved liver function, reduced bile salt toxicity, and improved survival (4).

3.2 NOVELTY

Due to the promising results of a multifactorial modulation strategy to attenuate IRI, as observed in our preclinical model, we now aim to translate this strategy clinically. Indeed, because of organ shortage and a persisting imbalance between LTx candidates and available organs, expanded criteria organs are increasingly used. The use of these "extended criteria" livers has been found associated with an enhanced IRI, which is the main cause of delayed graft function and PNF. Therefore, a multifactorial modulation strategy or combined drug approach is a beneficial intervention for expanded criteria donor organs that could lead to a reduction of IRI, delayed graft function and consequent hospital stay with associated health care cost; an enlargement of the donor pool with a safer use of the extended criteria donor and therefore a limitation of the waiting time and the concomitant risk to die whilst awaiting a LTx. Any intervention that will be beneficial for expanded criteria donor organs is also regarded to be beneficial for all other normal/uncompromised organs.

In particular, the development, after LTx using "normal livers", of biliary complications such as ischemic type biliary strictures represents an additional potential target that might benefit of administration of the multifactorial modulation.

The novelty of our study to attenuate IRI lies on the sequential administration of drugs acting simultaneously on several steps of the IRI cascade and its proof of concept in a clinically relevant stringent large animal model of LTx. Such combined drug approach has already been approved in other fields than LTx and is currently used in oncological treatment for instance.

All those efforts required to set up the multifactorial modulation were presented in numerous national and international conferences, published in a top-ranking surgical magazine (Annals of Surgery) and awarded on national and international meetings (*Best communication in basis science 2007, Belgian Week of Gastroenterology. Best abstract 2007, Belgian Transplantation Society. International young investigator award, American Transplant Congress, 2007)*.

Since then, publications on the multifactorial modulation have been cited by some authors as a required and innovative strategy to attenuate IRI. Professor Pierre-Alain Clavien (Zurich, Switzerland), expert in LTx and IRI referred to our multifactorial

CONFIDENTIAL

approach as following: *"However, from our point of view, the future of pharmacological strategies attenuating or preventing ischemia/reperfusion injury lies more in the combination of drugs acting simultaneously on several steps of the ischemia/reperfusion injury cascade"* (28).

Finally, we learned that a research group who was previously studying a single agent (glycine) against hepatic IRI (Prof Dr P Schemmer, http://www.controlled-trials.com/ISRCTN69350312 /) is now –similar to our multi-factorial approach- also focusing on more than one single agent to attenuate IRI.

3.3 FROM PRECLINICAL MODEL TO CLINICAL CAPITL STUDY

In contrast to the preclinical study, not all components are available for a clinical study. FR167653, a p38MAP Kinase inhibitor that we previously obtained from Astellas is no longer available. The reasons for not continuing were not officially communicated. Moreover, the plasma protein alpha-1-glycoprotein and glycine are not readily available for clinical use (not in accordance with the required GMP standards), and would require substantial product development effort from Sanquin or CAF-DCF which is currently not anticipated by this company. Finally, glycine is commercially not available.
We looked for an appropriate alternative for all components of the multifactorial strategy that could not be applied in the clinical trial:

- Infliximab: Neutralization of the overproduction of TNF-α,
- Antithrombin III: vasodilatation of the microcirculation through i.a. release of NO and reduction of NFκB expression and pro-inflammatory cytokine production,
- EPO: anti-apoptosis, anti-inflammation, anti-oxidant,
- Melatonine: anti-oxidant with synergistic action with respect to glutathione and vitamin E.

Extensive literature research learned us that some key factors of IRI as described and depicted in figures 4 and 5 such as the complement have been left untouched in the preclinical model. In addition, Vekemans et al. have demonstrated that the multifactorial modulation used in the preclinical model was found to suppress a range of inflammation-regulating genes but identified other potential targets as well (27).

Therefore, an intensive and up-to-date literature study has been undertaken at the start of this research project aiming to "update the multifactorial modulation protocol" (Table 3). Other components (including so called "biologicals" and "small molecules") were chosen on the basis of their clinical availability, short half-life, published efficiency on liver IRI, safety profile if administrated 1 time (except EPO) at a therapeutic and clinical recommended dose. Consequently, erythropoietin, C1 inhibitor, melatonin, antithrombin-III and infliximab have been added to the protocol.

Preclinical and safety studies have already been published for those additional components (cf appendix 3). In contrast to pharmaceutical sponsored trials that test entirely new compounds whose toxicity profile is not yet clear and that may have been stopped prematurely because of an increase in morbidity and mortality, our drugs are already used clinically in other indications (cf appendix 3).

CONFIDENTIAL

It has been anticipated that administration of the individual components of the combined drug approach is safe:

1. Most of the components selected represent natural sources: biological or endogenous compounds and are available for clinical use.

2. The clinical and safe use of these products is supported by extensive literature reports (dose, side effect, safety regulation, contraindication).

3. The components are known to be non-toxic with a single intravenously dose (or even repeated dose for EPO) and at the dose selected.

4. Independent feedback of pharmacologist experts (Prof Pieter Annaert, Laboratorium of Pharmacotechnology and Biopharmacy KU Leuven; Prof Paul Declerck, Laboratorium of Pharmaceutical biology KU Leuven) on the potential occurrence of drug-to-drug interactions (DDI) between the different components of the multifactorial modulation. (Cfr Addendum: Feedback regarding CAPITL study).

    a. The risk for PK type drug-drug interactions between the components is limited.
    b. At the given dose (which is not exceeding the normal physiological level for the biologicals or small molecules), it is anticipated that the pharmacokinetics of the components will not be affected.
    c. There is no concern for potential interactions with human serum albumin or with pharmaca used during the anesthesia for liver transplantation (chapter V, 5.1.2 Anesthesia).
    d. The different elements of the combined drug approach will be administered *sequentially, through separate infusion sets*, and *without direct contact* between the components (lines will be flushed with an isotonic electrolyte solution in between the administration of the components), for a detailed overview see figure 2.
    e. It is unlikely that the pharmacodynamics of the components will be affected because (except for C1 inhibitor and antithrombin-III) the components exert distinct effects through different mechanisms.

5. As recommended by the pharmacological experts, specific attention will be given regarding safety issues of individual components:

    a. No drugs will be administered during the administration of glutathione,
    b. No heparine will be administered during the liver transplantation to avoid enhancement of the anticoagulant properties of Antithrombin-III; in case of clinically relevant problems, hemostasis can be corrected.
    c. Patients previously treated with infliximab will be excluded.

CONFIDENTIAL

Product (Company)	*Mode of action*	*Half-life*	*Dose*	*Total volume required for reconstitution*	*Way and duration of administration*	*Timing of administration*	*Reported side effects at recommended dose*	*Main described side effects in literature (Cfr appendix 3)*
**Antithrombin III Atenativ®** (Octapharma)	- Increase the release of prostacyclin and NO.<br>- Reduction of inflammatory cell migration.<br>- Decrease the formation of ROS.	72 hours	3000 IU	60 cc	IV – 15 minutes	Start of anhepatic phase	No	Allergic reactions, arterial hypertension
**C1-inhibitor Cetor®/Cinryze®** (Sanquin/ViroPhar-ma (taken over by Shire))	- Inhibition of classical (+++), lectine (+) and alternative (+) pathway of the complement activation<br>- Regulation of intrinsic and fibrinolytic pathways of the coagulation cascade.<br>- Anti-inflammatory protein	42 hours	1000 U	10 cc	IV – 5 minutes	10 minutes before reperfusion	No	Allergic reactions, Anaphylactic shock
**EPO-β Neorecormon®** (Roche)	- anti-apoptotic<br>- Reduction of inflammatory cytokines.<br>- Antioxidant.	12 hours	30.000 IU<br>+<br>30.000 IU	0.6 cc<br>+<br>0.6 cc	IV – 2 minutes	13-15 minutes before reperfusion<br>+<br>6 hours after reperfusion	No	Thrombus, seizure, arterial hypertension
**Melatonin Circadin®** (Nycomed)	- Antioxidant.<br>- ROS scavenger.	4 hours	6 mg	3 capsules	Orally	On the ward before the transplantation	No	No
**Epoprostenol Flolan®** (GlaxoSmithKline)	- Vasodilatation.<br>- Antioxidant.<br>- Inhibition of platelet aggregation.<br>- Reduction of leukocyte activation and adhesion.	0.1 hour	500 µg	50 cc	Flush through the vena porta during the bench table	Ex-situ during the bench table before the implantation	Ex-situ administration (no foreseen systemic absorption)	In case of systemic absorption: headache, hypotension, arrhythmia, heart failure,

CONFIDENTIAL

**Glutathione Tationil 600®** (Roche Italy)	- Antioxidant.<br>- ROS scavenger.	0.25 hour	3 g	20 cc	IV – 2 minutes	2-4 minutes before reperfusion	No	No
**Infliximab Remicade®** (Janssen Biologics)	- Inhibition of inflammatory cascade by blocking both soluble and transmembrane forms of Tumor Necrosis Factor-α.	14 days	3 mg/Kg	7.5 cc/Kg	IV – 3 hours	Start of anhepatic phase after infusion of Antithrombin III<br><br>Interruption of the infusion during the administration of Glutathione<br><br>Restarted 15 minutes after reperfusion	Anaphylactic reaction first hours following administration	Anaphylactic shock, hematological effect
**Vitamin E suspension** 100 mg/ml (Cambridge Laboratories)	- Antioxidant.<br>- ROS scavenger.<br>- Increase the release of prostacyclin.	53 hours	500 mg	5 mL	Orally	On the ward before the transplantation	No	No
**Apotransferrin** (Sanquin)	- Non-transferrine bound iron (redox active) chelator.	/	170 mg/kg	3.4 cc/Kg	IV – 3 hours	Start of anhepatic phase<br><br>Interruption for sequential administration of erythropoietin, C1-inhibitor and Glutathione<br><br>Restarted 15 minutes after reperfusion	No	No

Table 3: List of components of the multifactorial modulation. For each component, the mode of action, the half-life, the dose and the reported adverse event at the recommended dose are described

CONFIDENTIAL

3.4 DESIGN

A two-part, investigator driven, adaptive study to assess the safety and to study the efficacy of a Combined drug Approach to Prevent Ischemia-reperfusion injury during Transplantation of Livers.

The first part of the study (part A) was a safety study conducted in 10 patients undergoing a liver transplantation which was regarded to be safe by the Data and Safety Monitoring Board and the results will be submitted to the local ethical committee.

The second part of the study (part B) foresees in a randomized controlled trial conducted in patients undergoing a liver transplantation at the University Hospitals Leuven, Belgium.

<u>Study part A: safety study</u>

A phase I, **safety study** will be first performed (Figure 1) in 10 patients in 4 smaller cohorts. The rationale is to establish the safety of the combined drug approach aimed to reduce ischemia-reperfusion injury during liver transplantation in eligible recipients. The combined drug approach, also referred as multifactorial modulation, foresees the *sequential* administration of drugs in eligible liver transplant recipients. Drugs will be administered as such that they will *never be in direct contact with each other.*

There will be 10 patients included in Part A, conducted in 4 consecutive small cohorts of 2, 2, 3, and 3 patients, respectively (Figure 1). For this safety study, all consecutive patients listed for LTx will be assessed for study eligibility by a senior staff physician at time of organ offer. The data and safety assessment of each cohort will then be reviewed by the Data and Safety Monitoring Board (DSMB) before patients are included in the next cohort (see section 16.1).

Safety will be assessed by well-defined objective criteria and parameters based on incidence and/or severity of adverse events indicating a potential health hazard caused by the multifactorial modulation within 7 days post LTx (see chapter XVI: Process leading to trial stopping rules). When serious adverse events are observed with probable causality linked to one of the components, then a withdrawal of the incriminated component(s) will be investigated and implemented by the DSMB if possible.

A positive feedback from the DSMB will allow to include the subsequent cohort of patients in the safety study. On the other hand, according the harshness, the recurrence and the limitation of acceptable side-effect rates of severe adverse events, the DSMB can stop the trial (cf. chapter XVI: Process leading to trial stopping rules).

CONFIDENTIAL

The period of time (7 days) has been determined keeping in mind the half-life and pharmacokinetic data of the different components in order to have sufficient time to observe potential adverse events. As shown in table 1, the half-life of Anti-thrombin III, C1-inhibitor, erythropoietin-beta, melatonin, glutathione and alpha-tocopherol does not exceed 3 days. The half-life of infliximab is 14 days but the most important potentially expected side effect is an acute allergic reaction within the first hours following administration. No pharmacokinetic data are available for apotransferrin but no side effects were observed in clinical trials at the dose proposed in the combined drug approach. Concerning epoprostenol, an ex-vivo administration is foreseen through the portal vein during the surgical preparation of the graft, as such, no systemic absorption is anticipated.
During the safety study, data on the pharmacokinetics of the orally administrated components will be obtained as requested by the local Ethical Committee. PK profiles will be determined based on measurements in blood samples obtained after anesthesia induction, just before skin incision, immediately prior to the start of the anhepatic phase, immediately prior to reperfusion (defined as opening of the portal vein), 30 minutes, 1 h, 2 h, 6h, 12h, 24h, 48h, 72h after reperfusion, daily from day 4 to day 7, as foreseen by the protocol (see section 9.5). These measurements will be done in the Laboratorium voor Farmacotechnologie en Biofarmacie, KU Leuven, O&N2, Campus Gasthuisberg, Leuven. When the anticipated levels are not reached, a dose change can be done for the next cohort.

At the end of Part A (safety study), the peak of Aspartate aminotransferase (peak AST) of all 10 patients will be also assessed by senior staff physicians and the DSMB, as requested by the local Ethical Committee. Only in case of a major increase compared to a historical control group, peak AST in the safety study will be then compared with patients from a historical matched control group. For this purpose, patients will be matched by the following list of variables: donor age (20-30, 31-40, 41-50, 51-60, 61-70, 71-80, 81-90), donor type [donation after brain death (DBD), donation after circulatory death (DCD)], cold ischemia time (CIT) (4-6h, 6-8h, 8-10h, 10-12h, 12-14h), Lab MELD score (6-19; 20-24; 25-29; 30-34; >35), cause of death (trauma vs. non-trauma). The matching will be based on a propensity score that is the probability to be a patient in the safety study based on the aforementioned list of variables. Each patient in the safety study will be matched with one or more patients from the control group having a similar propensity score, i.e. 1:N case-control match on the propensity score will be performed.

All data and other information of each patient included in the safety study will be recorded in an electronic Case Report Form (e-CRF). The e-CRF will be designed to allow continuous recording of variables during the study. The e-CRF and its maintenance are technically organized by an external company (EONIX, Mons, Belgium www.eonix.be). The e-CRF will be completed on a strict daily basis under the responsibility of the principal investigator.

At the end of Part A (safety study), the results (including pharmacokinetic profile for melatonin and vitamin E as well as peak AST results) will be submitted to the

CONFIDENTIAL

Ethical Committee and approval for part B (randomized controlled trial) will be requested if appropriate.

CONFIDENTIAL

Study part B: randomized controlled trial

Following approval by Ethical Committee, the second phase of the trial will be continued as an investigator driven, phase III, randomized study.

For this **RCT**, all consecutive patients listed for LTx at the University Hospitals Leuven, Belgium, will be assessed for study eligibility by a senior staff physician at time of organ offer. Patients will be randomly assigned to the treatment group (multifactorial modulation) or control group (standard of care treatment alone).

Eligible patients will be randomized at time of organ offer according to computer-based permuted blocks-generated randomization list by a third, independent party (EONIX).

3.5 OBJECTIVES

To demonstrate
- the safety of the multifactorial modulation (part A)
- the effectiveness of the multifactorial modulation in reducing the peak AST – a surrogate marker of ischemia-reperfusion injury (IRI) - after LTx (part B).

Peak AST is widely used as a surrogate reflecting the extent of IRI. This parameter correlates well with the parenchymal graft injury (29) following IRI and is associated with subsequent initial liver function or dysfunction after transplantation (30).

CONFIDENTIAL

IV. PARTICIPANTS

4.1 ELIGIBILITY CRITERIA

- Patient suffering from any type of irreversible liver failure eligible for LTx according to Eurotransplant guidelines.
- Patients ≥ 18 years of age at time of listing on the waiting list for LTx in at the University Hospitals Leuven, Belgium.

All consecutive patients listed for LTx will be assessed for study eligibility by a senior staff physician via the outpatient clinics or as an inpatient during their hospital stay.

4.2 INCLUSION CRITERIA

- Older than 18 years,
- Patients undergoing LTx at the University Hospitals Leuven, Belgium,
- Patient must have signed the patient informed consent,
- All donor types: donation after brain death (DBD), donation after cardiac death (DCD), standard criteria donors (SCD) and extended criteria donors (ECD) defined according Paris consensus (31).

4.3 EXCLUSION CRITERIA

- Patients who refuse to participate in the study,
- History of hypersensitivity to anti-thrombin III (Atenativ®), C1-inhibitor (Cetor®/Cinryze®), melatonin (Circadin®), epoprostenol (Flolan®), recombinant human EPO (Neorecormon®), infliximab (Remicade®), glutathione (Tationil®), tocopferol (vitamin e suspension 100 mg/mL®) will be excluded from the treatment group (cfr. Appendix 3.2).
- Conditions that prevent the use of the multifactorial modulation (cf. chapter XVIII, Appendix 3.2):
    - Administration of heparin at therapeutic dose pre-operatively: anti-thrombin III (Atenativ®), epoprostenol (Flolan®).
    - Congestive heart failure arising from severe left ventricular dysfunction: epoprostenol (Flolan®), infliximab (Remicade®).
    - History of seizures (not related to the underlying liver disease or to metabolic disturbances secondary to liver cirrhoris and to be distuinghuised from e.g. hepatic encephalopathy), poorly controlled arterial hypertension, myocardial infarction or stroke in the month preceding the liver transplantation, and history of pre-existing venous thromboembolic disease which is not related to liver cirrhosis and hypercoagulability (eg. partial, complete or previous vena porta thrombosis/ vena mesenterica thrombosis/ vena lienalis): recombinant human EPO (Neorecormon®).
    - Unstable angina pectoris: recombinant human EPO (Neorecormon®).

CONFIDENTIAL

  - Severe untreated infections such as sepsis, abscesses and opportunistic infections: infliximab (Remicade®).
  - Use of Vitamin K antagonist anticoagulation preoperatively which can not be reversed, women taking oral contraceptives containing oestrogens: tocopferol (vitamin E suspension 100 mg/mL®).
  - Patients with previous treatment of infliximab (Remicade®),

- Mental conditions rendering the subject incapable to understand the nature, scope, and consequences of the trial,
- Patients suffering from acute liver failure
- Combined organ transplantation,
- Re-transplantation,
- Patients that are dialysis-dependent prior LTx,
- LTx from a living or a split organ donation
- Administration of the multifactorial modulation technically non-feasible (i.e impossibility to place the second central catheter required for the *separate* and *sequential* injection of the different components of the multifactorial modulation).

CONFIDENTIAL

V. INTERVENTION

5.1 CONTROL GROUP: Standard care of treatment

5.1.1 Surgical procedure

The liver is first assessed and prepared for the implantation on the bench table (quality of the perfusion during procurement, degree of steatosis, vascular anomaly,...). During this phase, 1l of a CE labelled, preservation solution [including all CE marked preservation solutions such as University of Wisconsin (UW), Histidine Trytophan Ketoglutarate (HTK), IGL-1®, Celsior®,...] preservation solution is directly infused through the vena portae with the aim to rinse the liver from remaining blood, toxic metabolites and cellular debris. The orthotopic LTx consists of a hepatectomy phase, an anhepatic phase and a reperfusion phase. Following the hepatectomy, depending on the surgeon's and local center's preference, a veno-venous bypass is installed or not. During the anhepatic phase, the liver can be transplanted either by reconstructing both the supra- and infrahepatic cava vein (in case of veno-venous bypass) or either by a piggy-back technique (with or without creating a portocaval shunt). During the implantation of the graft, the graft may be rinsed out with 2 liters of Hartmann solution (isotonic, sodium 130 mmol/L, chlorine 109 mmol/L, lactate 28 mmol/L, potassium 4 mmol/L, calcium 1,35 mmol/L, pH 6,2) at room temperature. Alternatively, no rinsing of the liver is persued, according to the local center's standard treatment of care. Reperfusion is defined as the moment of restoration of blood flow into the liver graft after completion of the supra-/infra-caval anastomosis and the portal vein and/or hepatic artery. Thereafter, a biliary reconstruction is performed either by an end-to-end duct-to-duct anastomosis or a choledocho-enterostomy.

CONFIDENTIAL

5.1.2 Anaesthesia

Induction and maintenance of anaesthesia are performed according to the local standard treatment of care and typically includes curare (e.g. Cisatracurium or Rocuronium), analgesics (e.g. Sufentanil), a volatile anesthetic (e.g. Sevofluran) or an intravenous anesthetic (e.g. Propofol). A peripheral and central venous line, an arterial catheter, a Swan-Ganz, nasogastric tube, a urinary catheter and a temperature probe are installed. Administration of blood derivatives (red blood cells, fresh frozen plasma or blood platelets), Stable Solution of Plasma Protein (SSPP), albumin, colloids (Mannitol), and vasopressors are administered by the anaesthesiologist taking into account the hemodynamic and medical conditions. Infection prophylaxis (e.g. Amoxicillin, Cefotaxime and Metronidazole in case of choledocho-enterostomy) are given according the history of the patient prior to the intervention. In case of a known penicillin allergy, Ampicilline and Cefotaxime are replaced by Vancomycine and Levofloxacin.

Regular blood samples are taken to assess arterial blood gas analysis, ionogram, glycemia during the transplantation according the clinical need of the recipient:

- after anesthesia induction, just before skin incision,
- immediately prior to the start of the anhepatic phase,

- immediately prior to reperfusion (defined as opening of the portal vein),
- 30 minutes, 1 hr, 2 hrs after reperfusion.

Prior to, during and after LTx, the coagulation status of the patient may be severely disturbed and/or rapidly deteriorate, necessitating appropriate treatment by the anesthesiologists. Depending on the clinical need and to some extent guided by coagulation tests, peri-operative correction of coagulation is routinely done. Coagulation can be optimized by administration of e.g. platelets, fresh frozen plasma, selective administration of PPSB, or through administration of pro-coagulants (e.g. recombinant factor VIIa…) or anti-fibrinolytics (e.g. transexamic acid…). Of note, taking into account the well-known limitation of coagulation tests during LTx, optimizing the coagulation status will never be done based on the absolute values of the clotting tests alone. Nevertheless, the coagulation status will be routinely monitored by measuring prothrombin time (PT or INR), activated pro-thromboplastin time (aPTT), platelets, fibrinogen and through thromboelastography before LTx and 1 hour after reperfusion. Whenever regarded appropriate, the anesthesiologist may increase the frequency of monitoring the coagulation status.

Dobutamine, Levophed or other inotropics are given according to the hemodynamic status of the recipient as assessed by an anaesthesiologist (staff member).

5.1.3 Post-operative care in the intensive care unit

Early post-operative care consists out of monitoring and stabilizing of hemodynamic and pulmonary function, monitoring and prevention of surgical complications and prevention of infection.

Analgesia and sedation are adjusted according the liver function. Daily routine urine and blood samples are analysed to primarily monitor hematology, ionogram, liver and kidney function and levels of immunosuppression.

Post-LTx standard treatment of care includes prophylaxis against fungal infection (e.g. Nystatin sirup during 3 months, 2 ml QD), CMV infection (e.g. Valganciclovir, 450 mg OD or adjusted to the kidney function during 3 months in case of a CMV positive donor liver transplanted into a CMV negative recipient), pneumocystis carinii infection (e.g. Co-trimoxazole or Dapsone orally during 3 months). Post-operative antibiotic prophylaxis is continued for 48 hours (e.g. Cefotaxime, Amoxicilline, and additional Metronidazole in case of choledoco-enterostomy; in case of a known penicillin allergy, Amoxicilline and Cefotaxime are replaced by e.g. Vancomycine and Levofloxacin, respectively).

Patients will receive IV anti-hepatitis-B immunoglobulin in case of transplantation for hepatitis B followed by a lifelong treatment with antiviral oral Lamuvidine or alternative anti-viral medication if needed (e.g. related to side effects of Lamuvidine).

Finally, immunosuppression (cf chapter 5.6.1 Immunosuppression) is initiated and closely monitored.

During the postoperative stay in the intensive care unit in continuity with the stay on the nursing ward, blood samples are routinely collected at regular time points:

CONFIDENTIAL

- 6h, 12h, 24h, 48h, 72h after reperfusion
- Daily from day post LTx 4 to day 7, and day 14

Shortly after LTx, the coagulation status can still be severely disturbed, which is then treated accordingly by the intensivists. Postoperative optimization or correction of coagulation is routinely done, depending on the clinical need by administration of platelets, FFP, selective administration of PPSB, clotting factors or others depending of the clinical need. In addition, coagulation is monitored by blood samples to anticipate on coagulation problem. Therefore, prothrombin time, activated pro-thromboplastin time, platelets and fibrinogen are as routinely assessed during ICU stay.

Finally, daily production of ascites post-transplantation will be monitored via the wound drains as long as wound drains are present.

5.1.4 Post-operative follow up on nursing ward

Post-operative care on nursing ward consists of non-invasive monitoring clinical parameters (blood pressure, pulse, temperature, weight, intake and output), complications (surgical, immunological, infection and metabolic), the care for drains and catheters, correct administration of the medication and education of the patients.
In continuity with the stay in ICU, daily blood samples are routinely collected every morning in order to assess hematology, ionogram, liver and kidney function and levels of immunosuppression (whole blood trough levels of Tacrolimus before administration of the morning dose of Tacrolimus).
Daily urine samples are collected in order to assess diuresis and kidney function.
Ultrasound Doppler is routinely performed weekly in order to evaluate the parenchyma, vascularisation, possible bile duct abnormalities or intra-abdominal fluid collections. A liver biopsy is taken 1 week after LTx (except in case of contra-indication, e.g. suboptimal coagulation) or in case of clinical suspicion of AR.

5.1.5 Outpatient follow-up

After discharge, patients are re-assessed weekly/every two weeks during the first 3 months following the LTx. A clinical examination is performed (including clinical abdominal examination, blood pressure measurement, body weight). Blood samples are routinely collected in order to assess hematology, coagulation status (PT and INR) ionogram, liver and kidney function and levels of immunosuppression (whole blood trough levels of Tacrolimus). Thereafter, during the first year post LTx a monthly outpatient clinic assessment is scheduled. Blood samples are routinely collected at 3and 12 months.
At one year follow up, a routine MRCP is planned to investigate (i) the appearance of the parenchyma, (ii) the hepatic vasculature and (iii) the biliary tree.
Finally, daily production of ascites post-transplantation will be monitored via the wound drains as long as wound drains are present.

CONFIDENTIAL

5.1.6 Immunosuppression

5.1.6.1 Tacrolimus

The first dose of Tacrolimus will be administered orally or via the nasogastric tube post-LTx. The recommended daily initial dose is 0.05-0.1 mg/kg/day in 2 doses with 12 hours interval and will be adapted depending on the trough levels. Depending on the timing of the first dose, the time of the second dose is adjusted to fit the hospital routine. However, the second dose of Tacrolimus is not given less than 12 hours after the first dose.
Blood for the measurement of whole blood trough levels of Tacrolimus is drawn in the morning, before administration of the morning dose.
Dosing is then titrated during the first post-transplant week to give whole blood trough levels between 5-10 ng/mL, then, it will be adapted according to adverse events or clinical need.
To avoid any bias in the potential graft survival, switch from Prograft ® and Advagraf® to other Tacrolimus generics is not allowed; since there is no evidence of non-inferiority of these alternative formulations.

5.1.6.2 Mycophenolate Mofetil

The first dose of Mycophenolate Mofetil is administrated orally or IV post-LTx. The recommended daily dose is 0.5-1 g/day in 2 doses with 12 hours interval. Depending on the timing of the first dose, the time of the second dose is adjusted to fit the hospital routine. However, the second dose of Mycophenolate Mofetil is not given less than 12 hours after the first dose.
Trough levels with AUC for Mycophenolate Mofetil are not measured routinely. The adjustment of the doses is done according to side effects such as gastro-intestinal complications or leucopoenia.

5.1.6.3 Corticosteroids

The first dose of corticosteroids is administrated IV after transplantation. The regimen of corticosteroid therapy is as follows:

- Day 1 - 3: Methylprednisolone IV, 2 x 20 mg,
- Day 3 - day 21: Methylprednisolone per os (po), 16 mg in 2 doses,
- Day 21 - day 42: Methylprednisolone po, 12 mg in 2 doses,
- Day 42 - day 63: Methylprednisolone po, 8 mg in 2 doses,
- Day 63 - day 84: Methylprednisolone po, 4 mg in one dose,
- Day 84 - day 105: Tapering of the Methylprednisolone dose.

Steroids are tapered and withdrawn after 12 weeks under strict monitoring of liver function. After the end of week 12 at the latest, patient will no longer receive any steroid therapy unless it is clinically necessary (e.g. auto-immune hepatitis). No steroids are given in HCV positive recipients.

CONFIDENTIAL

Regimen doses are adjusted according to adverse events (e.g. rejection). In case of an acute rejection, high-dose pulsed corticosteroid therapy (500 mg IV Methylprednisolone is given during 3 consecutive days) is the first treatment of choice except for HCV positive recipients where doses of Tacrolimus are increased to trough levels of 10-15 ng/mL.

CONFIDENTIAL

5.2 TREATMENT GROUP: multifactorial modulation

This approach is based on the beneficial effects of the multifactorial modulation on graft and recipient survival previously observed in our harsh preclinical DCD-LTx model (4). Moreover, this multifactorial modulation was found to suppress inflammation-regulating genes in IRI (24).

Apart from receiving the standard care as described above, patients randomized to the treatment group receive:

1. C1-inhibitor
2. α-Tocopherol
3. Glutathione
4. Apotransferrin
5. Human Recombinant Erythropoietin beta (EPO-β)
6. Infliximab
7. Antithrombin-III
8. Epoprostenol
9. Melatonin

Detailed description on administration, dosing and treatment periods can be found in Table 1 and Figure 2.
As recommended by independent feedback of pharmacologist experts Prof Pieter Annaert (Laboratorium of Pharmacotechnology and Biopharmacy, KU Leuven) and Prof Paul Declerck , (Laboratorium of Pharmaceutical biology, KU Leuven) (Cfr Addendum Feedback regarding CAPITL study) C1-inhibitor, Glutathione, Apotransferrin, Human Recombinant Erythropoietin beta (EPO-β), Infliximab and Antithrombin-III will be infused intravenously (IV) **sequentially** and ***through different perfusion sets*** **without direct contact** during the anhepatic and the reperfusion phase. A second infusion of EPO-β is foreseen 6 hours after reperfusion. This IV sequential administration just before reperfusion aims to reach a plasmatic peak of concentration of each component directly after their infusions. In addition the infusion of infliximab is interrupted during the administration of Glutathione. Melatonin and α-Tocopherol will be given orally before the transplantation. According to the available pharmacokinetic data, plasmatic peaks of concentration should be reached after reperfusion. Epoprostenol will be added to 1L of preservation solution and infused ex-situ directly into the liver through the vena porta during the bench table before the implantation. To avoid any contact between the components, a short flush of isotonic electrolyte solution will take place between the sequential infusions of the components. The infusion of Remicade (anti-TNF-alpha antibody) starts before the reperfusion, is interrupted during administration of glutathione and then restarted 15 minutes after reperfusion (period of time required to stabilize the patient after the reperfusion). The infusion of apotransferrine (scavenger of non-transferrin-bound iron) starts before the reperfusion, is interrupted for the administration of EPO-β, C1-inhibitor and glutathione, and then restarted 15 minutes after reperfusion. As such, the manufacture's guidelines are followed (slow infusion rate for infliximab and larger volume for apotransferrine, respectively). Moreover, as our preclinical studies have shown: the peak of TNF-alpha and non-transferrin bound iron was observed 3 hours and 1 hour after the reperfusion, respectively.

CONFIDENTIAL

Except for Apotransferrin, all the components are registered and described in the MICROMEDEX Healthcare Series (6) and in the MARTINDALE (7).

Except for Apotransferrin, the administration of a single dose of each drug component, at the dose described in Table 1 (cfr summary), has been proven to be safe and efficient in reducing ischemia-reperfusion injury in both animals and clinical studies. A full description of current uses, precautions and contraindications, proposed mechanism of action, expected adverse effects, drug interactions, dosage form, and packaging can be found in Appendix 3.

CONFIDENTIAL

VI. TIME LINE & PARTICIPATING CENTER

6.1 TIME LINE (Figure 3)

- **2012:**
  - Finalising multi-factorial modulation protocol,
- **2013:**
  - Finalising multi-factorial modulation protocol
  - Start recruitment of patients,
  - Start inclusion of patients in the first safety phase of the study,
  - Start inclusion of patients in RCT phase of the study,
  - Start data analysis of patients included in safety phase study,
  - Inclusion of patients in the study if safety study is considered as safe,
  - Start data collection,
- **2014:**
  - Recruitment of patients in RCT phase continued,
  - Data collection continued.
- **2015:**
  - Recruitment of patients in RCT phase continued,
  - Data collection continued.
- **2016:**
  - Recruitment of patients in RCT phase continued,
  - Data collection continued.
- **2017:**
  - Data collection continued,
  - Start data analysis.
- **2018:**
  - Data analysis continued.

CONFIDENTIAL

6.2 PARTICIPATING CENTER

The recruitment of 10 patients for the safety study (part A) of the multifactorial modulation will be conducted in the University Hospitals of Leuven. This safety phase will be followed by a multi-center RCT (part B) at the University Hospitals Leuven, Belgium.

CONFIDENTIAL

VII. PRIMARY ENDPOINT (part B, RCT)

The primary objective of part B (RCT) this trial is to demonstrate effectiveness of the multifactorial modulation on the extent of IRI as reflected by the peak AST during the first week after LTx and later during follow-up.

Peak AST is widely used as a surrogate marker reflecting the extent of IRI. This parameter correlates well with parenchymal graft injury (29) ensuing IRI and is associated with the subsequent initial liver function or dysfunction after transplantation (30). Albeit simple, the peak of aspartate amino transferase is internationally accepted as the best surrogate of IRI and is included in all definitions or scoring systems for delayed graft function after LTx (30).

Therefore, peak AST will be assessed as the primary endpoint of this study. The peak is defined as the highest value of AST during the first 72 hours following the LTx.

AST analysis will be performed in the central lab of the University Hospitals Leuven by means of a colorimetric method, detection limit 4 U/L (Hitachi/Roche Modular P, Roche Diagnostics, Vilvoorde, Belgium).

CONFIDENTIAL

VIII. SECONDARY ENDPOINTS (part B, RCT)

- *Graft loss at 3, 12 months after LTx.* Graft loss is defined as the need for retransplantation within one week post LTx due to a non-life-sustaining liver graft function (PNF) or later (other reason).

- *Recipient death* at 3, 12 months after LTx.

- *Early graft dysfunction* as defined by Olthoff (1): the presence of one or more of the following postoperative laboratory analyses: bilirubin ≥ 10mg/dL on day 7, international normalized ratio ≥ 1.6 on day 7, and alanine aminotransferase (ALT) or AST > 2000 IU/L within the first 7 days.

- *Incidence of biliary strictures within 12 months post LTx:* a biliary stricture is defined as a narrowing within the biliary tree, radiologically evident [endoscopic retrograde cholangio-pancreatography (ERCP) and/or magnetic resonance cholangiopancreatography (MRCP)] to cause clinical symptoms or biochemical abnormalities requiring intervention (ERCP, percutanous transhepatic cholangiographic (PTC) drainage, surgery, retransplantation). Biliary strictures are categorized as anastomotic or non-anastomotic based on the cholangiographic appearance of the biliary tree as judged by a blinded radiologist. Non-anastomotic strictures are defined as any strictures, dilatation, or irregularity of the intra- or extrahepatic bile ducts of the liver graft at a site(s) other than that of the anastomosis. Intra-hepatic biliary strictures are classified in 4 groups: unilateral focal, confluence, bilateral multifocal and diffuse necrosis (2). Beside the routine 1 year post-transplant assessment of the biliary tree by MRCP and/or ERCP, biliary strictures will be investigated in case of clinical or biochemical suspicion (based upon cholestasis). Other causes leading to cholestasis (e.g. hepatic artery thrombosis, bile leakage, rejection or cholangitis) will be excluded based on state –of-the-art radiological and histological examination as part of the routine standard treatment of care. These examinations include ultrasound Doppler, CT and CT angiogram, biopsy-proven rejection based on histology scored by 2 blinded experienced liver pathologists according to the Banff criteria, ERCP and MRCP.

- *IRI score*: The extent of IRI will be assessed by a histological score based on the degree of cytoplasmic vacuolization, sinusoidal congestion, necrosis of parenchymal cells, apoptosis and influx of neutrophils as described by Suzuki score (3) and Monbaliu et al. (4). Liver biopsies will be taken before implantation (bench table), 1 hour after reperfusion and 1 week after transplantation and blindly scored by 2 pathologists.

- *Graft rejection*: a liver biopsy is taken after LTx at 1 week post-LT and in case of clinical suspicion of acute rejection. Clinical suspicion of acute rejection may be based on clinical symptoms (such as jaundice, low-grade fever) or sometimes nonspecific complaints (such as generalized malaise, decreased appetite) and/or biochemical abnormalities (usually increasing or plateauing levels -in an

CONFIDENTIAL

abnormal elevated range- of liver tests that were returning to normal values). Histological changes will be scored blindly according the BANFF criteria (see appendix 1) by 2 experienced liver pathologists.

- *Severe surgical complications*: the Clavien-Dindo classification (5) will be used to rank severe surgical complications ($\geq$ 3b)within 30 days after LTx according to an objective, simple, reliable, and reproducible manner. This classification is based on the therapy required to treat the complication (appendix 2, adverse event). The severity of the biliary strictures as well will be classified using this standardized grading system.

- *Acute Kidney Injury:* The rate of AKI is assessed by the Risk, Injury, Failure, Loss, and End-Stage kidney disease (RIFLE) criteria at 48 hours after graft reperfusion and defined by the fold change in serum creatinine from baseline (after anaesthesia induction) (14, 28). The severity of AKI was defined by the largest creatinine change observed, and classified as follows: <1.5 fold change compared with baseline was defined as normal renal function, >1.5 but <2 as acute renal risk (AKI-R), >2 but <3 as acute renal injury (AKI-I), and >3 or need for renal replacement therapy as acute renal failure (AKI-F).

- *Post-reperfusion syndrome:* The rate of post-reperfusion syndrome was defined as a 30% decrease of mean systemic blood pressure for more than 1 minute during the first 5 minutes following graft reperfusion (31).

CONFIDENTIAL

IX. VARIABLES OF INTEREST

9.1 DONOR CHARACTERISTICS

Age, ethnicity (black versus non-black), gender, cause of death (trauma, cerebro-vascular accident, anoxia), weight, height, Body Mass Index (BMI), donor type (DCD, DBD, ECD and SCD), diuresis last hour, duration of hypotensive period, presence of cardiac arrest, duration of donor warm ischemic time, time of start cold perfusion, time of hepatectomy, administration (yes/no) and dose (IU) of Heparin, perfusate solution type and volume, perfusion via aorta or portal vein or both, length of ICU stay, administration of vasopressors, laboratory values (peak and most recent value of AST, ALT, gamma-GT, creatinine, total bilirubin, sodium), CMV viral status, imported versus local liver procurement, calculation of the donor risk index as described by Feng et al. (32) and the Balance of Risk (BAR) score described by Dutkowski et al. (33), pretreatment with steroids (dose, nature of steroid, way of administration) (34).

9.2 RECIPIENT CHARACTERISTICS

Age, ethnicity (black versus nonblack), gender, weight, height, BMI, laboratory Model for End-stage Liver Disease (MELD) score the day of LTx, match MELD, time on waiting list, United Network for Organ Sharing (UNOS) score, diagnosis according the Eurotransplant classification, additional morbidity as hypertension (defined as a documented blood pressure above 140/90 mmHg and/or requiring at least one anti-hypertensive drug within 3 months before LTx), diabetes mellitus (type I or II defined as a hyperglycaemia requiring oral anti-diabetic or insulin within 3 months before transplantation), hemodialysis at the time of the LTx, duration of hemodialysis prior to LTx and past history of ascites (Grade 1 defined as mild with only visible on ultrasound and CT, grade 2 defined as detectable with flank bulging and shifting dullness, grade 3 with directly visible and confirmed with fluid thrill).

9.3 INTRA-OPERATIVE VARIABLES

Duration of surgery, piggy-back (with or without creating a portocaval shunt) versus veno-venous by-pass technique, cold ischemia time (CIT) (defined as the time between cold flush of the liver in the donor and its leaving from the melting ice water just before implantation), intra-operative warm ischemia time (defined as the time between the liver leaving the ice and the portal vein reperfusion), arterial anastomosis time (defined as the time between the liver leaving the ice and arterial reperfusion), transfusion need (packed red blood cells, fresh frozen plasma, and platelets), cardiac output 15 minutes after reperfusion, invasive cardiac hemodynamics reflecting right and left ventricular function, hemodynamic status after 15 minutes of the reperfusion (stable > 100 mmHg of systolic pressure, mild 80-100 mmHg, moderate 60-80 mmHg, severe < 60 mmHg), portal and arterial hepatic flow, urine output from the start of surgery until start of reperfusion, urine output from reperfusion to 120 minutes after reperfusion, urine output from beginning until the end of surgery and length of veno-venous bypass.

CONFIDENTIAL

9.4 POST-OPERATIVE VARIABLES

Length of ICU stay, length of hospital stay, PNF (defined as a non-life sustaining function of the liver graft, leading to death or re-LTx within 7 days), delayed graft-function as defined by Olthoff (1), 3, 12 months patient death and graft loss, date of death, biopsy proven rejection, adverse events (cfr. appendix 2).

9.5 LABORATORY ASSESSMENT

Blood samples are taken before, during and daily after LTx. Analysis of AST will be performed in the central lab of the University Hospitals Leuven.
10 cc of plasma will be collected at the following time points:

- After anesthesia induction, just before skin incision,
- Immediately prior to the start of the anhepatic phase,
- Immediately prior to reperfusion (defined as opening of the portal vein),
- 30 minutes, 1 hr, 2 hr after reperfusion,
- 6h, 12h, 24h, 48h, 72h after reperfusion,
- Daily from day 4 to day 7,
- Day 14 when the recipient is still an in patient, a 2 day window is allowed whenever the recipient has been discharged prior to day 14 and blood samples cannot be taken on day 14 (e.g. weekend)
- 3 months +- 2 weeks after transplantation,
- 12 months +- 4 weeks after the transplantation.

9.6 HISTOLOGICAL FEATURES

Liver biopsies are taken at the following time points:
- Before implantation at the bench as a baseline biopsy,
- Approximately 1 hour after the reperfusion,
- 1 week (max. 3 weeks) after the transplantation,
- On indication (e.g. suspicion of acute rejection).
- Biopsies of the caudal part of the common bile duct (ring of 1 mm) will be taken and stored in formaline
- Before implantation at the bench
- After reperfusion and before closure

The tissue will be fixated in formaldehyde 6% and embedded in paraffin. After hematoxylin and eosin staining, blind analysis will be performed by 2 experienced pathologists in the pathological department of the University Hospitals Leuven in order to assess the IRI score and graft rejection.

CONFIDENTIAL

X. DATA ANALYSIS

10.1 REPORTING OF THE DATA (part B, RCT)

An intention-to-treat analysis will be done and all participating patients will be included. Data will be reported according to the CONSORT criteria.
All data and other information of each patient included in the trial will be recorded in an electronic Case Report Form (e-CRF). The e-CRF will be designed to allow continuous recording of variables during the study. The e-CRF and its maintenance are technically organized by an external company (EONIX). The e-CRF will be completed on a regular basis under the responsibility of the local principal investigator.

10.2 STATISTICAL METHODOLOGY

10.2.1 Primary outcome

The log-transformed peak AST values will be compared between both groups using a linear model with group and centre as factors. If variances between both groups differ significantly (based on a likelihood-ratio test comparing the models with equal and unequal variances, respectively), the result from the model with unequal variances will be reported.
The following analyses will be added to verify the robustness of the obtained conclusion: firstly, a comparison of the groups will be done after correction for MELD score and cold ischemic time using a linear model on the log-transformed peak AST values. Secondly, all analyses will be repeated as a subgroup analysis, excluding the (expected) small set of patients with a DCD liver.

10.2.2 Sample size calculation

Sample size calculation is based on peak AST values obtained from a series of patients (N=308) that underwent LTx at the University Hospitals Leuven between January 2007 and October 2011. For 264 patients, peak AST value was available. These values followed a lognormal distribution; the log-transformed AST was normally distributed with mean and standard deviation equal to 6.53 and 0.93 respectively. The corresponding geometric mean equalled 685. Based on this distribution, 34.3%, 20.1%, and 12.6% of the subjects are expected to have an AST higher than 1000, 1500, and 2000, respectively. It is assumed that the treatment will lead to a 50% reduction of the (geometric) mean. This implies that 12.6%, 5.7%, and 3.0% of the subjects in the treatment group are expected to have an AST higher than 1000, 1500, and 2000 IU/L respectively.

Based on a two-sided two-sample pooled t-test of a mean ratio with lognormal data, 58 subjects are needed in total to have 80% power (with alpha set at 5%). Anticipating a

CONFIDENTIAL

drop-out rate of 20% and aiming to have complete blocks in the randomisation, 72 patients (36 per group) will be included in the study.

10.2.3 Secondary outcomes

Kaplan-Meier estimates will be used to construct curves for graft and recipient survival, which will be compared between groups with a stratified log-rank test (if there are deaths without graft dysfunction, cumulative incidence estimates will be considered for graft survival).

A Cox regression model will be used to compare graft and recipient survival between both groups after correction for MELD score and ischemic time (and center).

To compare early graft dysfunction and presence of biliary strictures between both groups, an exact test for the common odds ratio will be used. A correction for MELD score and ischemic time is considered with a logistic regression model, unless the number of events is too low. Deaths not known to be related to biliary strictures will not be included in the biliary strictures analysis (hence assuming that these are unrelated to the probability of biliary strictures). The severity of biliary strictures will be described in both groups.

For all outcomes (primary and secondary), it will be verified if the difference between groups varies between centers.

All analyses will be performed using SAS software, version 9.2 of the SAS System for Windows. Copyright © 2002 SAS Institute Inc. SAS and all other SAS Institute Inc. product or service names are registered trademarks or trademarks of SAS Institute Inc., Cary, NC, USA.

CONFIDENTIAL

XI. RANDOMIZATION (part B, RCT)

All consecutive patients listed for LTx will be assessed for study eligibility by a staff surgeon via the outpatient clinic (pre-transplant evaluation) or as an inpatient during their hospital stay. If patients are willing to participate to this first in man study and provide informed consent, a member of the LTx team will inform patients about the study, orally and in writing.

The randomization to the control group or to the treatment group is done pre-operatively, immediately after the liver has been allocated to a patient.

A third party – not involved in this trial – provides a distant central randomization of patients to the control or to the intervention group. Following inclusion in one group, the study coordinator will be informed by the third party randomizer with the details of the randomization to ensure correct verification of group allocation.

In each participating centres, patients will be randomised into two groups using variable block size (e.g. 2, 4, 6, ...). No stratification is performed on the a priori defined potential confounders MELD score, ischemic time and DCD. The expected set of DCD is too small to consider as a separate stratum and an analysis on historical data showed no evidence for a relation between peak AST with ischemic time and only a weak relation with MELD score.

XII. ALLOCATION CONCEALMENT

Distant randomization and allocation concealment of the patients to treatment is performed with the use of an electronic randomization table and a web-interface. The electronic randomization and its maintenance are technically organized by an external company (EONYX).

XIII. ETHICAL CONSIDERATION

The protocol is written conform the declaration of Helsinki (Appendix 5) and the investigators and their personnel will act according to the principles therein.
The investigators agree to conduct this study in accordance with the International Conference on Harmonization (ICH) principle of Good Clinical Practice (GCP).
The investigator will conduct all aspects of this study in accordance with all national and local laws of the applicable regulatory agencies. The study protocol will be approved by the institutional review board of the University Hospitals Leuven before the start of patient inclusion.

CONFIDENTIAL

13.1 ETHICAL COMMITTEE

The protocol, patient brochure and informed consent will be approved by the Ethical Committees before the start of patient inclusion. Approval will be obtained in writing, stating the identity of the clinical trial, the date of review, the documents reviewed and a list of the names and titles of the committee members.

Any substantial amendment to the protocol will be submitted to the Ethical Committee.

The investigators will be responsible of informing their Ethical Committee of all problems involving risks to patients according to national regulations.

13.2 PATIENT INFORMATION AND INFORMED CONSENT

Each patient participating in the trial will give his/her informed consent prior to entry into the trial. Informed consent will be given in writing after full written and verbal information has been provided by the investigator or a nominated representative (Appendix 6). In addition to the patient, the investigators or a nominated representative will sign the informed consent form. If necessary, it is acceptable for a patient's witness to sign the informed consent form on behalf of the patient.

The patient will receive the information brochure (Appendices 7 and 8 for part A, and part B, respectively) and can withdraw from the trial at any time without prejudice.

13.3 PERSONAL DATA PROTECTION

The investigators uphold the principle of the patient's right to protection against invasion of privacy. All data recorded in the e-CRF or passed on for further evaluation will be coded by patient number, initials and date of birth. Identification is restricted to authorized persons. The data will be anonymized correspondingly in all data analyses.

CONFIDENTIAL

XIV. WITHDRAWALS AND DROPOUTS

To avoid withdrawal and dropout bias, an intention-to-treat analysis will be performed. Participants will be included in the analyses as part of the groups to which they were randomized, regardless of whether they completed the study or not. No imputation techniques will be considered for missing outcome values, but differences in characteristics will be explored between patients with and without a missing outcome value.

The reasons for withdrawal are:

- Withdrawal of consent: Every patient is free to withdraw from the study for any reason and at any time without giving reason for doing so and without penalty or prejudice.

- The investigator is also free to terminate a patient's involvement in the study at any time if warranted by the patient's clinical condition (e.g. anaphylactic symptoms related to one of the substances in the multifactorial bio-modulation during infusion).

- Patient lost to follow-up.

The investigator will maintain a chronological patient identification list of all patients who enter into the trial containing the patient details (name, study number, initials, date of birth, age, sex), date and time of entry into the study (see Appendix 8).
The number of withdrawals, dropouts and the reasons will be stated in this enrolment/withdrawal form. If there is no withdrawal, it will also be stated.

Patients who are discontinued will be treated and followed without any disadvantage with regard to medical care or physician-patient relationship.

The investigators will maintain a chronological patient screening list of all patients who will be transplanted during the study period but who will not enter the study. This list must contain patient details, date of transplantation, and reason for non-participation (see appendix 9).

CONFIDENTIAL

XV. INTERIM ANALYSIS

During part B (RCT), no interim analyses will be performed to stop the study early due to efficacy or futility reasons.

CONFIDENTIAL

XVI. PROCESS LEADING TO TRIAL STOPPING RULES

16.1 DATA SAFETY MONITORING BOARD

An independent safety board will be composed by staff members from the departments of intensive care (Prof dr FERDINANDE and Prof dr em. LAUWERS), biostatistics (Kris Bogaerts) and chaired by Prof dr em. FEVERY (former chief of the Hepatology department). They will handle concerns about safety and termination of the study in its first or second phases in case of increased incidence and/or severity of adverse events indicating a potential health hazard caused by the multifactorial modulation.
The DSMB will –independently from the investigators- decide whether to continue or to stop the trail based on ***predefined*** serious adverse events (see 16.2.4) and the potential drug causality.

16.2 DESCRIPTION OF ADVERSE EVENT

16.2.1 Definition of adverse event (AE) and serious adverse event (SAE)

According the GCP (Clinical Trial Directive 2001/20/EC), an ***adverse event (AE)*** is defined as any untoward medical occurrence in a patient or subject of the treated group during an experiment, and which does not necessarily have a causal relationship with this treatment. This definition includes physical signs, symptoms and laboratory test values. At study enrolment, laboratory values that fall outside the relevant reference range will not be reported as adverse events. Clinically significant modifications of values during the study period will be reported.

A ***serious adverse event (SAE)*** is any untoward medical occurrence or effect that suggests a significant hazard, contraindication, side effect or a precaution for human patients. The term includes any event that results in:

- Death,
- Life threatening condition,
- Persistent significant disability or incapacity,
- Prolongation of initial hospitalisation or re-hospitalisation,
- Requiring intervention to prevent one of above.

16.2.2 Grade of severity

- *Mild (grade 1)*: patient is aware of symptoms but tolerates them easily. Symptoms does not interfere with daily activity.
- *Moderate (grade 2)*: patient experiences discomfort that interferes with normal activity. No treatment is required except acetaminophen.
- *Severe (grade 3)*: patient is unable to carry out normal activity. Treatment is required.
- *Life-threatening (grade 4)*: emergency room visit or disabling or hospitalization.

CONFIDENTIAL

16.2.3 Timeframe of observation

1. During the LTx.

2. Immediately after LTx until day 7.

16.2.4 Predefined serious adverse events

The predefined serious adverse events will be considered in the decision to continue/to stop the trial by the DSMB Some of these predefined SAE's listed are inherently related to LTx, regardless of the multifactorial modulation protocol (e.g. post reperfusion syndrome, cardiac arrhythmia…), therefore the definition of SAE for this study also includes "refractory to standard treatment" whenever appropriate.

- Medical complications: kidney dysfunction, ascites, pleural effusion, delirium, urinary infection, de novo diabetes, respiratory insufficiency, other.
- Viral infection: CMV, EBV, HBV, HCV, HSV type 1, HSV type 2, Varicella Zoster, Influenza, polyomavirus, other.
- Bacterial infection: pneumonia, gastro-intestinal, clostridium difficile, GI other, sepsis, other.
- Fungal infection: aspergillus fumigatus, candida albicans, cryptococcus neoformans, other.
- Parasite infection : toxoplasmose gondii, trpanosoma cruzii, other.
- Thrombo-embolic complication: pulmonary embolism, deep venous thrombosis, other.
- Cardio-vascular event: infarct, de novo heart failure, arrythmia.
- Cerebro-vascular event: ischemia, haemorrhage.
- Specific graft complication: PNF, EAD, infection of preservation solution, aortic patch or perfusate.
- Surgical arterial problem: hepatic artery thrombosis, hepatic artery stenosis, mycotic aneurysm.
- Caval and hepatic vein complication: portal vein thrombosis, portal vein stenosis.
- General surgical complication: bleeding, wound dehiscience, wound infection, evisceration, seroma, lymphocele, other.
- Bile ducts complication: leal, anastomotic strictures, ischemic cholangiopathy, choledoco-lithiasis, cholangitis.
- Immunological complication: acute antibody-mediated rejection, acute cellular rejection

16.3 Protocol-defined adverse events

The following adverse events are commonly observed during or after liver transplantation and are therefore not considered as adverse events for the purpose of the trial:

CONFIDENTIAL

- Gastrointestinal problems (nausea, constipation and/or diarrhoea) related to the use of immunosuppression (such as Mycophenolate acid derivatives)
- Hypertension as a pre-existing disease or induced by immunosuppression
- Headaches related to immunosuppression
- Anaemia, leukopenia or thrombocytopenia related to immunosuppression,
- Transient hyper/hypocalcemia, hyper/hyponatremia, hyper/hypokalaemia, hyper/hypophosphataemia, hypomagnesemia - Peripheral oedema and hypoalbuminemia in the peri-operative period related to filling status, peri-operative management and recovering liver function (until first 3 months after liver transplantation)
- Post- liver transplantation delirium related to pre-existing encephalopathy and post-operative and ICU delirium

16.4 DRUG CAUSALITY

The causality of an AE in relation to the study therapy as a whole will be categorized as follows:

- *Highly probable*: Apparent relationship in time between AE and drug administration or drug concentration in body and fluids or tissues. Relationship between AE and drug is already known or expected. Reaction has occurred with this medication previously and there is an appropriate temporal relationship between therapy and AE.
- *Probable*: Known pharmacological effect with no possible other cause and appropriate temporal association.
- *Possible*: AE likely to be associated with the drug and no other medication was taken, or known pharmacological effect of medication that could also be associated with another concomitant therapy, illness or external cause.
- *Unlikely*: Unlikely to be causally related: e.g. reaction occurred after cessation of drug therapy or is more likely to be due to another concomitant therapy, illness or external cause.
- *Definitely not*: AE known to be caused by another concomitant therapy, illness or external cause.
- *Not assessable*: Likelihood of AE not known, or relationship of AE to study therapy, another concomitant therapy, illness or external cause is not clear. This category should be used very scarcely.

16.5 INDIVIDUAL AND COHORT LEVELS

The safety analysis will be conducted by the independent DSMB.

The description of severe adverse events for safety analysis will be prospectively collected according to a strict procedure (Figure 9).

CONFIDENTIAL

**Patient ID**			
**Date of transplantation**			
**Date of severe adverse event**			
**Description**			
**During the LTx**	☐	Death	
	☐	Anaphylactic shock	(Life-threatening type of allergic reaction)
	☐	Post reperfusion syndrome	(30% decrease of MAP (compared to the end of the anhepatic phase) during 1 min within the 5 min after reperfusion)
	☐	Severe cardiac arrythmia	(Leading to hemodynamic disturbance)
	☐	PaO2/FiO2 ratio	(Cfr SOFA score)
	☐	Abnormal level of platelets	(Cfr SOFA score)
	☐	Hypoglycemia	(hypoglycemia- 45-75 mg/dL)
	☐	Hypo-/Hyper-kaliema	(Level of potassium < 3,5 and > 5,0 mEq/L respectively)
	☐	Abnormal hyperlactataemia	(Level of lactate > 5 mmol/L)
**From day 1 to day 7**			
	☐	Death	
	☐	Primary non function	(Need for retransplantation within one week post LTx due to a non-life-sustaining liver graft function)
	☐	Peak AST > 2000 UI/L	
	☐	Kidney dysfunction	(Cfr SOFA score)
	☐	Sepsis	(Whole-body inflammatory state caused by severe infection)
	☐	Bleeding	(Requiring radiolgical and/or surgical intervention)
	☐	Myocardial infarction	(Interruption of blood supply to a part of the heart, causing heart cells to die)
	☐	Severe cardiac arrythmia	(Leading to hemodynamic disturbance)
	☐	Thrombosis	(Clot formation in vein/artery)
**Grade**	☐		(Grade I - Mild - No interference)<br>(Grade II - Moderate - Interference but no treatment required)<br>(Grade III - Severe - Intereference and treatment required)<br>(Grade IV - Life-threatening)
**Causality in relation with the study**			
	☐	Highly probable	(Apparent relationship in time between severe adverse event and drug administration)
	☐	Probable	(Known pharmacological effect with no possible other cause and appropriate temporal association)
	☐	Possible	(known pharmacological effect of medication that could also be associated with another concomitant therapy)
	☐	Unlikely	(Unlikely to be causally related. E.g. reaction occurred after cessation of drug therapy)
	☐	Definitively not	(Severe adverse event caused by another concomitant therapy)
	☐	Not assessable	(Likelihood of severe adverse event not known)

Figure 9: Description of serious adverse events for safety analysis.

For each patient, grade of severity, time of onset, type of adverse event, SOFA score and drug causality will be daily detailed.
For each cohort of patients, number of subjects experiencing similar adverse events will be described.

According the harshness, the recurrence and the limit of acceptable side-effect rates of severe adverse events, the DSMB can decide to stop the trial.

XVII. REFERENCES

1. Validation of a current definition of early allograft dysfunction in liver transplant recipients and analysis of risk factors. Olthoff et al., Liver Transplant. 2010.
2. Classification and prognosis of intrahepatic biliary stricture after liver transplantation. Won Lee et al., Liver Transplantation 2007; 13: 1736-1742.
3. Neutrophil infiltration as an important factor in liver ischemia and reperfusion injury. Suzuki et al., Transplantation 1993, 55; 1265-1272.
4. Multifactorial biological biomodulation of warm ischemia reperfusion injury in liver transplantation from non-heart-beating donors eliminates primary non-function and reduces bile salt toxicity. Monbaliu et al., Ann Surg. 2009; 250: 808-17.
5. The Clavien-Dindo classification of surgical complications, five years experience. Clavien et al., Ann Surg 2009, 250: 187-196.
6. Thomson. Micromedex. Drug Information for the Health Care Professional. 25th ed. plus Updates. Content Reviewed by the United States Pharmacopeial Convention, Inc. Greenwood Village, CO. 2005.
7. https://www.medicinescomplete.com/mc/martindale/current/
8. Utilization of extended donor criteria in liver transplantation: a comprehensive review of the literature. Nickkholg et al., Nephrol Dial Transplant 2007, 22: 829-836
9. Influence of marginal donors on liver preservation injury. Briceno et al., Transplantation 2002, 74: 522-526
10. Preoperative factors associated with outcome and their impact on resource use in 1148 consecutive primary liver transplants. Markmann et al., Transplantation 2001, 72: 1113-1122
11. Ischemia and reperfusion- from mechanism to translation. Eltzschig et al., Nature medicine 2011; 11: 1391-1401.
12. Molecular mediators of liver ischemia and reperfusion injury: a brief review. Vardanian et al., Mol Med 2008, 14: 337-345
13. Mechanism of hepatic ischemia/reperfusion injury and protection against reperfusion injury. Kang et al., Transplantation proceedings 2002, 34: 2659-2661.
14. de Groot Rauen et al. Ischemia reperfusion injury: processes in pathogenic networks: a review Transpl Proc 2007; 39: 481
15. Preservation and reperfusion injuries in liver allografts. An overview and synthesis of current studies. Clavien et al., Transplantation 1992, 53: 957-978.
16. Biliary tract complications after liver transplantation: a review. Wojcicki et al., Dig Surg 2008; 25: 245-257.
17. Effects of donor pretreatment with dopamine on graft function after kidney transplantation, a randomized control trial. Schnuelle et al., JAMA 2009, 302: 1067-1075.
18. Clinical trial of the Pan-caspase inhibitor, IDN-6556, in human liver preservation injury. Baskin-Bey at al., Transplantation 2007; 7: 218-225.
19. HEPGOL: Randomized, placebo controlled multicenter, double-blind clinical trial to investigate hepatoprotective effects of glycine in the postoperative phase of liver transplantation (ISRCTN69350312). Luntz et al., BMC Surg 2005, 5: 18.
20. Inhaled NO accelerates restoration of liver function in adults following orthotopic liver transplantation. Lang et al., The Journal of clinical investigation 2007; 2583-2591.
21. Thymoglobulin induction protects liver allografts from ischemia/reperfusion injury. Bogetti et al., Clin Transplant 2005, 19; 507-511.
22. Tacrolimus as a liver flush solution to ameliorate the effects of ischemia/reperfusion injury following liver transplantation. St Peter et al., Liver Transpl 2003, 9; 144-149.

CONFIDENTIAL

23. Impact of stable PGI(2) analog iloprost on early graft viability after liver transplantation: a pilot study. Bärthel E et al., Clin Transplant 2012, 26 ; E38-E47.

24. Monbaliu et al. Livers from non-heart-beating donors tolerate short periods of warm ischemia. Transplantation 2005.

25. Monbaliu et al. Increased serum Phospholipase A2 activity after non-heart-beating donor LTx and association with ischemia-reperfusion injury. J Surg Res. 2009.

26. Monbaliu D, Liu Q, Vekemans K et al. Potentiation of adverse effects of cold by warm ischemia in circulatory death donors for porcine LTx. Transplant Proc 2012, 44: 2874-2879.

27. Improving the function of liver grafts exposed to warm ischemia by the leuven drug protocol: exploring the molecular basis by microarray. Vekemans et al., Liver Transplant 2012, 18: 206-218.

28. De Rougemeont, Dutkowski, Clavien: Biological modulation of liver ischemia-reperfusion injury. Current Opinion in Organ transplantation 2010, 15, 184-189

29. Diagnosis and monitoring of hepatic injury. Performance characteristics of laboratory tests. Dufour et al., Clinical Chemistry 2000; 46: 2027.

30. Analysis of initial poor graft function after orthotopic liver transplantation: experience of an australian single liver transplantation center. Nanashima A et al., Transplant Proc 2002; 34:1231-1235.

31. Report of the Paris consensus meeting on expanded criteria donors in liver transplantation. Durand et al., Liver Transplant 2008; 14: 1694-1707.

32. Characteristics associated with liver graft failure: The concept of a donor risk index. Feng et al., Am J Transpl 2006; 6: 783-790.

33. Are there better guidelines for allocation in liver transplantation? A novel score targeting justice and utility in the model for end-stage liver disease era. Dutkowski et al., Ann Surg 2011; 254: 745-753.

34. Methylprednisolone therapy in deceased donors reduces inflammation in the donor liver and improves outcome after liver transplantation. Kotsch et al., Ann Surg 2008; 1042-1050.

CONFIDENTIAL

XVIII. APPENDICES

APPENDIX 1: BANFF CRITERIA

Blinded analysis by 2 independent pathologists will be performed and histological changes scored according to BANFF criteria (Banff schema for grading liver allograft rejection: an international consensus document. Banff working group, Hepatology 1997; 25: 658-663).

Category	Criteria	Score
Portal Inflammation	- Mostly lymphocytic inflammation involving, but not noticeably expanding, a minority of the triads.<br>- Expansion of most or all of the triads, by a mixed infiltrate containing lymphocytes with occasional blasts, neutrophils and eosinophils.<br>- Marked expansion of most or all of the triads by a mixed infiltrate containing numerous blasts and eosinophils with inflammatory spillover into the periportal parenchyma.	1<br>2<br>3
Bile duct inflammation damage	- A minority of the ducts are cuffed and infiltrated by inflammatory cells and show only mild reactive changes such as increased nuclear: cytoplasmic ratio of the epithelial cells.<br>- Most or all of the ducts infiltrated by inflammatory cells. More than an occasional duct shows degenerative changes such as nuclear pleomorphism, disordered polarity and cytoplasmic vacuolization of the epithelium.<br>- As above for 2, with most or all of the ducts showing degenerative changes or focal luminal disruption.	1<br>2<br>3
Venous endothelial inflammation	- Subendothelial lymphocytic infiltration involving some, but not a majority of the portal and/or hepatic venules.<br>- Subendothelial infiltration involving most or all of the portal and/or hepatic venules.<br>- As above for 2, with moderate or severe perivenular inflammation that extends into the perivenular parenchyma and is associated with perivenular hepatocyte necrosis.	1<br>2<br>3

CONFIDENTIAL

APPENDIX 2: ADVERSE EVENTS

1. Definition of adverse events

According the GCP (Clinical Trial Directive 2001/20/EC), an ***adverse event (AE)*** is any untoward medical occurrence in a patient or subject of the treated group during an experiment, and which does not necessarily have a causal relationship with this treatment. This definition includes physical signs, symptoms and laboratory test values. At study enrolment, laboratory values that fall outside the relevant reference range will not be reported as adverse events. Clinically significant modifications of values during the study period will be reported.

A ***serious adverse event (SAE)*** is any untoward medical occurrence or effect that suggests a significant hazard, contraindication, side effect or a precaution for human patients. The term includes any event that results in:

- Death,
- Life threatening condition,
- Persistent significant disability or incapacity,
- Prolongation of initial hospitalisation or re-hospitalisation,
- Requiring intervention to prevent one of above.

Hospitalisation is not assessed as a SAE if hospitalisation:

- Ensues due to routine procedures,
- Was planned before study entry or occurs without being scheduled before study entry for a pre-existing non worsening condition,
- Is not fulfilling the criterion of untoward medical occurrence (e.g. social and/or convenience admissions, rehabilitation program).

The severity of an AE is to be determined as follows:

- *Mild (grade 1)*: patient is aware of symptoms but tolerates them easily. Symptom does not interfere with daily activity.
- *Moderate (grade 2)*: patient experiences discomfort that interferes with normal activity. No treatment is required except acetaminophen.
- *Severe (grade 3)*: patient is unable to carry out normal activity. Treatment is required.
- *Life-threatening (grade 4)*: emergency room visit or disabling or hospitalization.

The causality of an AE in relation to the study therapy as a whole will be categorized as follows:

- *Highly probable*: Apparent relationship in time between AE and drug administration or drug concentration in body and fluids or tissues. Relationship between AE and drug is already known or expected. Reaction has occurred with this medication previously and there is an appropriate temporal relationship between therapy and AE.
- *Probable*: Known pharmacological effect with no possible other cause and appropriate temporal association.
- *Possible*: AE likely to be associated with the drug and no other medication was taken, or known pharmacological effect of medication that could also be associated with another concomitant therapy, illness or external cause.

CONFIDENTIAL

- *Unlikely*: Unlikely to be causally related. E.g. reaction occurred after cessation of drug therapy or is more likely to be due to another concomitant therapy, illness or external cause.
- *Definitely not*: AE known to be caused by another concomitant therapy, illness or external cause.
- *Not assessable*: Likelihood of AE not known, or relationship of AE to study therapy, another concomitant therapy, illness or external cause is not clear. This category should be used very scarcely.

A ***suspected unexpected serious adverse reaction (SUSAR)*** is an untoward medical effect. The nature or severity of which is not consistent with the information on the experiment, and with the applicable product information, which results in death, is life-threatening, requires hospitalisation or prolongation of existing hospitalisation, results in persistent or significant disability or incapacity, or when it is a congenital anomaly or birth defect.

2. Detection, reporting, and responsibilities

On an ongoing basis, the investigators will determine whether any clinical or laboratory AE has occurred. Only the moderate or severe (needing a treatment) AEs will be notified center and recorded in the e-CRF describing date of onset, date of cessation, maximum intensity, severity, causality, therapy and outcome. The assessment of the potential association with the drugs will be handled by the DSMB in case of safety concern.

Occurrence of any SAE or any event that deserves reporting according to the investigator will be reported on an ongoing basis.
SAE and SUSAR will have to be reported within 24 hours. New SAE or SUSAR occurring within 28 days after the patient has completed the clinical trial or 28 days after the patient is withdrawn from the study must be reported.

The investigators will inform the competent authorities and the ethical committee (UZ Leuven) in the due times requested by them. They will also publish an annual safety report to be sent to all competent authorities and leading ethical committee, containing all reported suspected serious adverse reactions (SSARs).

CONFIDENTIAL

3. Surgical complication classification (within 30 days after transplantation)

The Clavien-Dindo classification will be used to rank surgical complications according to an objective, simple, reliable and reproducible way. This classification is based on the type of therapy required to treat the complication.

Grades	Definition
**Grade I**	Any deviation from the normal postoperative course without the need for pharmacological treatment or surgical, endoscopic and radiological interventions.<br>Acceptable therapeutic regimens are: drugs as antiemetics, antipyretics, analgetics, diuretics and electrolytes, and physiotherapy.<br>This grade also includes wound infections opened at the bedside.
**Grade II**	Requiring pharmacological treatment with drugs other than such allowed for grade I complication.<br>Blood transfusions and total parenteral nutrition are also included.
**Grade III**	Requiring surgical, endoscopic or radiological intervention.
**Grade III-a**	Intervention not under general anesthesia.
**Grade III-b**	Intervention under general anesthesia.
**Grade IV**	Life-threatening complication (including CNS complications) requiring IC/ICU-management.
**Grade IV-a**	Single organ dysfunction (including dialysis).
**Grade IV-b**	Multi-organ dysfunction.
**Grade V**	Death of patient.
**Suffix "d"**	If the patient suffers from a complication at the time of discharge, the suffix "d" (for disability) is added to the respective grade of complication. This label indicates the need for a follow-up to fully evaluate the complication.

CNS: brain hemorrhage, ischemic stroke, subarachnoid bleeding, but excluding transcient ischemic attack; IC: intermediate care; ICU: intensive care unit (Clavien-Dindo et al. 2009; 250: 187-196)

CONFIDENTIAL

4. Infection

Each patient will be routinely monitored for bacterial, fungal or viral infection on an ongoing basis (HBV, CMV, Epstein-Barr virus, and HCV recurrence). Details about any infection detected and therapeutic measures applied must be recorded.

4.1 CMV

For clarity purposes about CMV infection, the following definitions are applied.

4.1.1 CMV infection

CMV infection is defined as detection of:

- Anti-CMV IgM antibodies in a previously seronegative patient,
- or CMV early-antigen (CMV pp-65) in any body fluid (blood, urine, saliva),
- or positive DEAFF-test in any body fluid (blood, urine, saliva),
- or intraleukocytic CMV-DNA by PCR amplification,
- or CMV DNA or antigens in tissue biopsies.

4.1.2 CMV disease

CMV disease is defined as:

- Evidence of CMV infection as described above, and
- fever > 37.9 °C for 3 days or more,
- and/or leucopenia < 4000 WBC/ml,
- and/or thrombocytopenia,
- or invasive CMV as defined by detection of any CMV material (nucleic acids or antigens) in any tissue biopsy (lung including bronchiolo-alveaolar-lavage, gastro-intestinal tract).

4.1.3 CMV syndrome

CMV syndrome is defined as: patient with fever, fatigue, muscular skeletal pain, headache and CMV infection criteria who does not fulfill the CMV disease criteria.
Screening for CMV infection or CMV disease during follow up will be done on an ongoing basis. Occurrence of either CMV infection or CMV disease will be recorded with pertaining details.

4.2 Hepatitis C

As hepatitis C virus (HCV) infects the liver allograft immediately after transplantation and is an uncommon cause of allograft dysfunction during the first several weeks after transplantation, HCV recurrence will be monitored on an ongoing way in patients at risk.

CONFIDENTIAL

5. Predefined types of adverse events

**Patient ID**			
**Date of transplantation**			
**Date of adverse event**			
**Description**			
**Non severe**	☐	Urinary tract infection	(Leucocyturia, culture > 100,000 /mL, no fever)
	☐	Upper respiratory tract infection	(pharyngitis or bronchitis without fever)
	☐	Anemia	(Hb level < 8 mg/dL)
	☐	Leukopenia	(WBC 4500-11000/mm3)
	☐	Electrolyte disturbance	(Cfr SOFA score)
	☐	Seroma	(Accumulation of fluid other than blood or pus in a space with previous surgery)
	☐	Wound abcess	(Not deeper than the fascia)
	☐	Cardiac arrythmia	(Without any hemodynamic consequences)
	☐	Incisional hernia	
**Severe**			
	☐	Pyelonephritis	
	☐	Pneumonia	(Need for retransplantation within one week post LTx due to a non-life-sustaining liver graft function)
	☐	Malignancy	
	☐	Myocardial infarction	(Cfr SOFA score)
	☐	Sepsis	(Whole-body inflammatory state caused by severe infection)
	☐	Bleeding	(Requiring radiological and/or surgical intervention)
	☐	Myocardial infarction	(Interruption of blood supply to a part of the heart, causing heart cells to die)
	☐	Severe cardiac arrythmia	(Leading to hemodynamic disturbance)
	☐	Thrombosis	(Clot formation in vein/artery)
	☐	Biliary leak	
	☐	Intraperitoneal abcess	
	☐	Peritonitis	
	☐	Biliary anastomotic stricture	
	☐	Biliary non-anastomotic stricture	
**Clavien-Dindo scale** (surgical complication within 30 days)	☐		(Grade I – deviation from the normal postoperative course, no need for pharmacological treatment or interventions<br>(Grade II – requiring pharmacological treatment, blood transfusions and total parenteral nutrition are also included)<br>(Grade III – requiring surgical, endoscopic or radiological intervention)<br>(Grade IV – life-threatening complication requiring IC/ICU-management)<br>(Grade V – death of patient)
**Causality in relation with the study**			
	☐	Highly probable	(Apparent relationship in time between severe adverse event and drug administration)
	☐	Probable	(Known pharmacological effect with no possible other cause and appropriate temporal association)
	☐	Possible	(known pharmacological effect of medication that could also be associated with another concomitant therapy)
	☐	Unlikely	(Unlikely to be causally related. E.g. reaction occurred after cessation of drug therapy)
	☐	Definitively not	(Severe adverse event caused by another concomitant therapy)
	☐	Not assessable	(Likelihood of severe adverse event not known)

APPENDIX 3: COMBINED DRUG APPROACH

3.1 GENERAL DESCRIPTION

3.1.1. ANTITHROMBIN III, ATENATIV®

Introduction

Antithrombin III (ATIII) is a plasma-derived serine protease inhibitor (serpin). Independently and besides its potent anticoagulant activity, ATIII has a direct action on activated endothelial cells by increasing the release of prostacyclin and NO. Moreover, ATIII reduces vessel wall transmigration and subsequent tissue damage. Finally, ATIII decreases the formation of ROS and proteases by neutrophils (1). A potential underlying mechanism was recently characterized by the experimental proven reduction of nuclear factor kappa beta expression resulting in less systemic release of cytokines (2).
The AT-III-mediated reduction of microcirculatory disorders and tissue injury has been proven as highly effective in various models of renal (3), intestinal (4) and hepatic ischemia-reperfusion (5-6). In addition, ATIII reduced rejection in renal xenotransplantation (7). In clinic, Fertmann et al. has characterized a significant improvement of renal allograft reperfusion by single-shot application during human kidney transplantation in a randomized controlled trial (8). They also observed a decreased peak of Tumor Necrosis Factor-alpha (TNF-alpha) after kidney transplantation (2). Further, the same group has described a reduction of reperfusion injury, pancreatitis and a prevention of graft thrombosis with a single-shot of ATIII in human pancreas-kidney transplantation (9). Moreover, ATIII has been shown to reduce rejection in allogenic cardiac transplant (10). Currently, a multi-centric protocol for clinical phase III trial testing pre-reperfusion ATIII application in combination with acetylcysteine is ongoing for simultaneous kidney and pancreas transplantation.
Pharmacokinetic studies with ATIII have shown a mean biological half-life of about 3 days.

Safety and adverse reaction

Concerning the safety, Fertmann et al. (9) did not observe perioperative ATIII-mediated changes on standard coagulation tests (partial thromboplastin time, thromboplastin time, platelet counts), on the perioperative bleeding rates and on the number of packed red blood cell concentrates given to the patient with a single IV dose of 3000 IU. This finding is in agreement with the literature where long-term ATIII application also did not influence these standard blood coagulation tests (11).
ATIII replacement during administration of heparin in therapeutic dosage increases the risk of bleeding. The effect of ATIII is enhanced by heparin. The half-life of ATIII may be considerably decreased with concomitant heparin treatment due to accelerated ATIII turnover (1.5 days).
Hypersensitivity or allergic reactions (which may include angioedema, burning and stinging at the infusion site, chills, flushing, generalized urticaria, headache, hives, hypotension, lethargy, nausea, restlessness, tachycardia, tightness of the chest, tingling, vomiting, wheezing) have been observed infrequently in conjunction with the use of

CONFIDENTIAL

Antithrombin III concentrates and may in some cases progress to severe anaphylaxis (including shock). On rare occasions, fever has been observed.

Contra-indication linked to the use of Atenativ®

Patient requiring therapeutic dose of heparin perioperatively will be excluded from the treatment group.

Product characteristics

The ATIII Atenativ® is manufactured by Octapharma. This product is prepared from pooled units of human plasma from normal donors and contains no preservative. It is a lyophilized powder in a vial and a solvent in a vial which is used for reconstitution of the powder.

Packaging and storage

Atenativ® is presented as powder for solution for infusion containing nominally 500, 1000 or 1500 IU human plasma-derived antithrombin III per vial. The product contains approximately 50 IU/ml human plasma-derived antithrombin III when reconstituted with 10 (500IU), 20 (1000 IU) or 30 (1500 IU) ml of water for injection respectively.
The vial of powder should be stored at a temperature of 2-8°C. It should stay in the outer carton in order to be protected from light.
Within its shelf-life, the product may be stored at room temperature (25°C) for up to 1 month, without being refrigerated again during this period, but must be disposed if not used after this.
After reconstitution, the product should be used as soon as possible. If not used immediately, in-use storage times should not be longer than 24 hours at 2 to 8°C.

Dose and administration

According these experimental and clinical data that have proven profound effect without toxicological events, 1 single IV dose (3000 IU) of ATIII (Atenavit®) will be administrated after the start of anastomosis during the anhepatic phase (50 minutes before reperfusion) over 15 minutes.

References:

1. Antithrombin: a new look at the actions of a serine protease inhibitor. Roemisch et al., Blood coagulation and fibrinolysis 2002; 13: 657-670.
2. Effects of antithrombin on cytokine secretion after human allogenic kidney transplantation. Fertmann et al., Transplantation 2004; 78: 222-223.
3. Antithrombin III reduces renal ischemia-reperfusion injury in rats. Ozden et al., Res Exp Med 2001; 200: 195.
4. Antithrombin III prevents and rapidly reverses leukocyte recruitment in ischemia/reperfusion. Ostrovsky et al., circulation 1997; 96: 2302.
5. Antithrombin reduces IRI of rat liver by increasing the hepatic level of prostacyclin. Harada et al., Blood 1999; 93:157.
6. Controlled trial of antithrombine III supplementation in fulminant hepatic failure. Langley et al., J hepatol 1993; 17: 326.
7. Protective effects of recombinant human antithrombin III in pig-to-primate renal transplantation. Cowan et al., Am J Transpl 2002; 2: 520.

CONFIDENTIAL

8. High dose antithrombine therapy reduces IRI and improves graft function during human allogenic kidney transplantation: final results of a randomized controlled clinical trial. Fertmann et al.,Transplantation 2002; 74, 352.
9. Single-shot antithrombin in human pancreas-kidney transplantation: reduction of reperfusion pancreatitis and prevention of graft thrombosis. Fertmann et al., Transplant International 2005; 18: 40.
10. High dose of AT induces indefinite survival of fully allogenic cardiac grafts and generates regulatory cells. Arakami et al., Transplantation 2003; 75: 217.
11. Effect of long-term and high-dose antithrombin supplementation on coagulation and fibrinolysis in patients with severe sepsis. Hoffmann et al., Crit Care Med 2004; 32: 1851.

CONFIDENTIAL

3.1.2. C1-ESTERASE INHIBITOR, CETOR®/Cinryze®

Introduction

C1-inhibitor (C1-INH) is a blood-derived serine protease inhibitor (serpin). C1-INH is the key player in the regulation of complement activity. It acts mainly by inhibiting the classical pathway but also with a lower effect on the lectine and alternative pathways. Moreover, it also balances the intrinsic pathway and the fibrinolytic system of the coagulation. Finally, it reduces the expression of IL-6, thereby preventing damage generated by neutrophil recruitment and subsequent neutrophil activation (1). Therefore, C1-INH has emerged as a promising agent to inhibit damage resulting from activation of inflammation and coagulation.
Studies have provided evidence for the role of complement in the pathogenesis of IRI (2). The use of C1-INH for the inhibition of the complement in various models of myocardial (3), lung (4), renal, intestinal and hepatic ischemia-reperfusion (5) has been proven as highly effective in the reduction of inflammation and consequent tissue injury. Moreover, besides the routine clinical practice use of C1-INH in angioedema, clinical trials have focused on the decrease of vascular leakage syndrome after lung transplantation (6) and the reduction of myocardial IRI. Bauernschmitt et al. (7), Thielmann et al. (8) and Fattouch et al. (9) have all described a significant positive impact of C1-INH on the cardiac function during coronary surgical reperfusion. Stieh et al. has observed the same findings with a post-operative administration of C1-INH in open heart surgery. Finally, C1-INH may also be used to prevent antibody induced acute graft rejection after the transplantation. This however requires longer-term administration after the procedure (10, 11).

Safety and adverse reaction

Toxicity of C1-INH is very low. No side effects were observed in those clinical trials (6, 7, 8, 9). No complications are expected in patients either with a liver dysfunction or during the LTx.
Hypersensitivity or allergic reactions (which may include angioedema, burning and stinging at the infusion site, chills, flushing, generalized urticaria, headache, hives, hypotension, lethargy, nausea, restlessness, tachycardia, tightness of the chest, tingling, vomiting, wheezing) could be observed with C1-INH and may in some cases progress to severe anaphylaxis (including shock).

Contra-indication linked to the use of Cetor®/Cinryze®

There is no specific contraindication linked to the use Cetor®/Cinryze® in the multifactorial modulation (12, 13).

Product characteristics

The C1-INH Cetor®/Cinryze® is manufactured by Sanquin Blood Supply Foundation/ViroPharma (taken over by Shire). It is purified from the human plasma. It is a sterile, stable, lyophilized preparation. The elimination half-life has been determined at 42 hours. The mean residence time (the time required for 62.3% of the administered dose of C1- inhibitor to be eliminated; comparable with the elimination half-life but calculated independently of the model used) is 65 hours. This applies equally to individuals with or without C1- inhibitor deficiency. The clearance in man is 0.053 L per hour.

CONFIDENTIAL

Packaging and storage

The commercial packing of Cetor®/Cinryze® consists of:

- A vial of Cetor®/Cinryze® 500 U (powder for solution for IV injection),
- A 5 ml vial of water for injections,
- An administration set consisting of 10 ml disposable syringe, transfer needle, filter needle, and butterfly-wing needle.

After dissolution in the water for injections supplied, the product contains 100 U C1-INH per ml; 5 ml = 500 U C1-INH.
Cetor®/Cinryze® should be administered as soon as possible and no later than 3 hours after piercing the vial. The entire solution should be used in a single administration.
The vial of product and water should be stored at a temperature of 2-8 °C.

Dose and administration

In this study, the IV dose of Cetor®/Cinryze® (Cinryze® will be used in September 2015 when the expiration date of vials of Cetor® is reached) has been determined according the experimental and clinical data that have proven profound effect without toxicological events. Thus, 1000 U will be administrated gradually, as recommended during 5 minutes at the end of the anhepatic phase (10 minutes before reperfusion). This time course is adequate according the half-life of Cetor®/Cinryze® after IV injection and we still expect a high plasma concentration after reperfusion.
During administration, the solution should not be too cold. The powder also dissolves more readily if both vials are brought to room temperature in advance (15-25°C).

References:

1. Recent advances in the use of C1-inhibitor as a therapeutic agent. Bergamaschini et al., Mol Immunol 2003; 40: 155-158.
2. Ischemia and reperfusion: from mechanism to translation. Eltzschig and Eckle, Nature medicine; 11: 1391.
3. Intracoronary application of C1 esterase inhibitors improves cardiac function and reduces myocardial necrosis in an experimental model of ischemia and reperfusion. Horstick et al., Circulation 1997; 95: 701-708.
4. C1-esterase inhibitor reduces reperfusion injury after lung transplantation. Scherer et al., Ann Thorac Surg 2002, 73: 233-238.
5. Endothelial targeting with C1-inhibitor reduces complement activation in vitro and during ex vivo reperfusion of pig liver. Bergamaschimi et al., Clin Exp Immunol 2001; 126: 412-420.
6. C1-esterase inhibitor in graft failure after lung transplantation. Struber et al., Intensive care med 1999; 25: 1315-1318.
7. Rescue therapy with C1-esterase inhibitor concentrate after emergency coronary surgery for failed PTCA. Bauernschmitt et al., Intensive care medicine 1998; 24: 635-638.
8. Administration of C1-esterase inhibitor during emergency coronary artery bypass surgery in acute ST-elevation myocardial infarction. Thielmann et al., Eur J Cardio Thorac Surg 2006; 285-293.
9. Beneficial effects of C1-esterase inhibitor in ST-elevation myocardial infarction in patients who underwent surgical reperfusion: a randomized double-blind study. Fattouch et al., Eur J Cariothorac Surg 2007; 32: 326.

CONFIDENTIAL

10. Recombinant human C1-inhibitor inhibits cytotoxicity induced by allo- and xenoantibodies. Poirier et al., Transplant Proc. 2008 Mar;40(2):581-3.

11. Recombinant human C1-inhibitor prevents acute antibody-mediated rejection in alloimmunized baboons. Tillou et al. Kidney Int. 2010 Jul;78(2):152-9.

12. Treatment of hereditary angioedema with nanofiltered C1-esterase inhibitor concentrate (Cetor): Multi-center phase II and III studies to assess pharmacokinetics, clinical efficacy and safety. J.J. Hofstra, I. Kleine Budde, E. van Twuyver, G. Choi, M. Levi, F.W.G. Leebeek, J.G.R. de Monchy, P.F. Ypma, R.J. Keizer, A.D.R. Huitema, P.F.W. Strengers. Clin Immunol 142 (2011) 280-90.

13. Model-Based Evaluation of Similarity in Pharmacokinetics of Two Formulations of the Blood-Derived Plasma Product C1 Esterase Inhibitor. R.J. Keizer, I. Kleine Budde, P.F. Strengers, M. Levi, J.H. Beijnen, A.D. Huitema. J Clin. Pharmacol.2011.

CONFIDENTIAL

3.1.3. ERYTHROPOIETIN BETA, NEORECORMON®

Introduction

EPO is a cytokine known to stimulate the proliferation and the survival of erythroid progenitor cells. However, the expression of EPO receptors has been described in several organs outside the bone marrow. Recently, increasing attention has been given to the pleiotropic effect of EPO. It has been demonstrated that recombinant human EPO (rhEPO) has relevant tissue-protective effects against various injuries in animal models, including liver IRI in pigs (1). The use of EPO in animal models has been reviewed by Sharples et al (2) and Johson et al (3). Virtually every organ that has been evaluated has been reported to benefit from EPO administration following IRI. The exact mechanism by which EPO mediates its organ-protective effects is not completely understood, but anti-apoptotic, anti-inflammatory and anti-oxidative properties have been demonstrated (4).

A growing number of clinical studies are testing the tissue protection effect afforded by rhEPO in various acute clinical settings, including neuroprotection and cardioprotection. Ehrenreich et al (5) has used EPO beta at a total dose of 100.000 IU for the first 3 days after an acute cerebral stroke, noting that this was safe and well tolerated. Lipsic et al (6) has demonstrated that a single bolus injection of EPO at a dose of 60.000 IU in patient with an acute myocardial infarction was not associated to any significant increase in hemoglobin (Hb). Mocini et al (7) has described that a single injection of EPO at a dose of 40.000 IU before cardiac surgery has no significant impact on the cardiac function and on the erythropoiesis. Belonje et al (8) has demonstrated in a safety pilot assessment that 60.000 IU of EPO in patients with an acute myocardial infarction was not associated with a raise in blood pressure, an increase in Hb and thrombocytes or any adverse events like vascular thrombosis or seizure. In elective liver surgery, Mosato Kato et al (9) has indicated that a two time injection of 30.000 IU EPO rather than one single bolus of 60.000 IU has a stronger inhibitory effect on IRI following the pringle maneuver during liver surgery. Moreover, these doses and their timing of administration did not affect blood cell count, hematocrit and platelets.

Considering the results of the different species experiments and clinical studies in different organs, it is suggested to adapt the dose of EPO and its timing of administration in order to reach an optimal protective effect. This was confirmed by Ramakrishnan et al. (10). They have described the importance of doses, dosage regimens and routes of administration in physiologically mechanistic PK/PD model. Pharmacokinetic investigations in healthy volunteers show that the half-life of IV administrated EPO beta is between 3 and 12 hours.

Notably, the receptor mediating the non-erythropoietic effects of EPO differ from the one responsible for hematopoiesis. The tissue-protective receptor exhibits a lower affinity for EPO. It is a heteromer consisting of EPO receptor monomers in association with the common receptor. This heteromeric receptor is expressed immediately following injury, whereas EPO production is delayed providing a window of opportunity for therapeutic intervention. Thus early administration of EPO can dramatically reduce the deleterious components of the local inflammatory cascade. However, a larger dose is required to trigger the protective effect as the affinity for the heteromer is lower than the hematopoietic receptor. Thus, to effectively treat injured tissue using exogenous EPO, high parenteral doses are required (11).

CONFIDENTIAL

Safety and adverse reaction

Safety data arising from clinical studies suggest that a high single IV dose of rhEPO is safe regarding acute as well as chronic adverse effects including vascular thrombosis, seizure and raise in blood pressure, Hb levels, platelets and hematocrit. Anaphylactoïd reactions were observed in isolated cases.
However, as patients with a history of seizure and a level of Hb higher than 13 gr/dl were excluded in a few studies, this treatment (and the rest of the multifactorial modulation) will be avoided, for safety concerns in patients with these findings.
The clinical results obtained so far do not indicate any interaction of NeoRecormon with other medicinal products.

Contra-indication linked to the use of Neorecormon®

Patient with a history of seizure and a pre-operative level of Hb higher than 13 g/dl will be excluded from the treatment group.

Product characteristics

The EPO-beta Neorecormon® is manufactured by Roche as pre-filled syringe. It is a colourless solution for injection.

Packaging and storage

One pre-filled syringe with 0.6 ml solution for injection contains 30.000 international units (IU).
The pre-filled syringe should be stored in a refrigerator (2°C – 8°C). It should stay in the outer carton in order to be protected from light.

Dose and administration

Considering the distribution profile after IV injection, the half-life of EPO-beta (3-12h), the potential elimination of the administrated medication by blood loss during the procedure and the need to keep a high concentration for the post-reperfusion phase, a first dose of 30.000 IU will be administrated 13-15minutes before the reperfusion of the liver over 2 minutes followed by a second dose of 30.000 IU of EPO-beta 6 hours after the reperfusion.

References:

1. Shimoda et al, hepato-gastroenterology 2009; 56: 470-475. EPO strongly protects the liver from IRI in a pig model.
2. Sharples et al, Nat Clin Pract Nephrol 2005; 1: 87-97. Mechanisms of disease: cell death in acute renal failure and emerging evidence for a protective role of EPO.
3. Johnson et al, Nephrology 2006; 11: 306-312. Novel renoprotective action of EPO: new use for an old hormone.
4. Ghezzi et al, Cell deat and differenciation 2004; 11: 37-44. EPO as an antiapoptotic, tissue-protective cytokine.
5. Ehrenreich et al, Molecular medicine 2002; 8: 495-505. EPO therapy for acute stroke is both safe and beneficial.
6. Lipsic et al, J Am Coll Cardiol 2006; 48: 2161-2167. Protective effect of EPO in cardiac ischemia: from bench to bedside.

CONFIDENTIAL

7. Mocini et al, Perfusion 2008; 23: 187-192. Endogenous EPO and a single bolus of 40.000 IU do not protect the heart from IRI during extracorporeal circulation for cardiac surgery.

8. Belonje et al, Am Heart J 2008; 155: 817-822. Effects of EPO after an acute myocardial infarction: Rationale and study design of a prospective, randomized, clinical trial.

9. Mosato Kato et al, World Journal of Gastroenterology 2010; 16: 4838-4845. EPO ameliorates early ischemia-reperfusion injury following the pringle maneuver.

10. Ramakrishnan et al, J Clin Pharmacol 2004; 44: 991-1002. PK and PD modeling of recombinant human EPO after a single and multiple doses in healthy volunteers.

11. Brines et al, Blood Purif 2010; 29: 86-92. The therapeutic potential of EPO for tissue protection: a tale of two receptors.

CONFIDENTIAL

3.1.4. MELATONIN, CIRCADIN®

Introduction

Melatonin is a pineal hormone involved in the circadian rhythm of vertebrates. Besides this role, Melatonin and its metabolites as well are known to exhibit strong antioxidant properties. Among any antioxidant agents, Melatonin is described as a most powerful endogenous free radical scavenger being 5 - 14 times more effective at scavenging the highly toxic hydroxyl radicals than Glutathione and Mannitol, respectively; it is also reported by twice more efficient than Vitamin E in detoxifying the peroxyl radical (1). Moreover, Melatonin shows synergistic effects with other antioxidants. When Melatonin is combined with Vitamin E or Glutathione, the protective effects against iron-induced lipid peroxidation is dramatically enhanced (1). Furthermore, Melatonin stimulates a number of antioxidative enzymes such as superoxide dismutase, catalase and glutathione peroxidase. Finally, Melatonin promotes the efficiency of the mitochondrial electron transport chain leading to a reduction of electron leakage, free radical formation, and improvement of energy production (1).
Via these combined beneficial actions, Melatonin has proven highly effective in reducing IRI in various models and clinical settings (2). In a warm ischemic model, Vairetti et al. has shown a significant reduction of transaminases in the liver of rats and pigs after preoperative administration of melatonin in a dose dependent manner (1). Chen et al. has described an enhancement of neutrophil apoptosis after administration of Melatonin during partial hepatectomy (3). More recently, Gitto et al. has shown the efficacy of IV Melatonin to decrease the oxidative stress status and pro-inflammatory cytokines in human neonates undergoing surgery for congenital malformation, observing that it was safe and well tolerated (4).
The antioxidative effects of Melatonin require concentrations that are much higher than endogenously produced plasma concentrations. On the other hand, a very low concentration of exogenous Melatonin (> 0.3 mg) produces already supra-physiological levels in humans. After an exogenous administration of Melatonin, very high concentrations are found in the hepato-biliary system due to an extensive first-pass and high affinity binding sites in hepatocyte nuclei. The serum Melatonin peak was reached after approximately 15 minutes from oral administration with an elimination half-life of 30-45 minutes. Additionally, half-life from crushed Melatonin and administrated through the naso-gastric tube is extended to 90 minutes (5). Indeed, the area of intestinal mucosa exposed to Melatonin is larger (6). This half-life is extended to approximately 100 minutes in cirrhotic patients (7) after oral administration. With the use of a prolonged and sustained release formula, the serum Melatonin peak is reached after 2-4 hours.

Safety and adverse reaction

Cost and toxicity of Melatonin is remarkably low. In the United States, the Food and Drug Administration (FDA) has designated Melatonin as an orphan product sold as a dietetic complement (8). No significant adverse effects were observed following administration of high doses (3-6.6 g/day) in humans for 15 to 35 days (9). High doses of IV Melatonin have been used with success in babies with sepsis syndromes without toxicological effect (10). IV infusion in doses between 10 and 60 mg during abdominal aortic-aneurysm repair has already been shown to be without immediate toxic reaction (11).
Melatonin's metabolism is mainly mediated by CYP1A enzymes. Therefore, interactions between Melatonin and other active substances as a consequence of their effect on

CONFIDENTIAL

CYP1A enzymes are possible. By inhibiting the isoenzymes CYP1A2, Quinolone, fFuvoxamine, Cimetidine and Oestrogene can increase the plasma level of Melatonin. On the other hand, Carbamazepin and Rifampicin may decrease the Melatonin exposure by stimulating its metabolism.

Contra-indication linked to the use of Circadin®

There is no specific contraindication linked to the use Circadin® in the multifactorial modulation.

Product characteristics

Circadin® is produced by Takeda-Nycomed. Tablets are white, round and biconvex.

Packaging and storage

The tablets are packed in PVC/PVDC opaque blister strips with aluminium foil backing. The pack consists of one blister strip containing 20 or 21 tablets, or two blister strips containing 15 tablets each (30 tablets). The blisters are then packed in carton boxes. Not all pack sizes may be marketed. Do not store above 25°C and keep the original package in order to protect from light.

Dose and administration

In this study, the oral dose of Melatonin has been determined according the experimental and clinical data that have proven profound effect without toxicological events (6). Thus 6 mg of melatonin will be administrated orally at the ward just before transfer to the operative theatre. This time course is adequate according the pharmacokinetic data of melatonin after oral administration (6).

References:

1. Exogenous melatonin enhances bile flow and ATP levels after cold storage and reperfusion in rat liver: implication for liver transplantation. Vairetti et al., J Pineal Res 2005, 38: 223-230.
2. A unicenter, randomized, double-blind, parallel-group, placebo-controlled study of melatonin as an adjunct in patients with acute myocardial infarction undergoing primary angioplasty (MARIA). Dominguez-Rodriguez et al., Contemporary Clinical Trial 2007, 28: 532-539.
3. Altered neutrophil apoptosis activity is reverse by melatonin in liver ischemia reperfusion. Chen et al., J Pineal Research 2003, 34:260-264.
4. Melatonin reduces oxidative stress in surgical neonates. Gitto et al., J Pediatr Surg2004, 39: 184-189.
5. Pharmacokinetics of orally administered melatonin in critically ill patients. Mistraletti et al., J Pineal Res 2010; 48: 142-147.
6. Melatonin pharmacokinetics following two different oral surge-sustained release doses in older adults. Gooneratne N et al., J Pineal Res 2012; 52: 437-445.
7. Melatonin serum levels and metabolic clearance rate in patients with liver cirrhosis. Igushi et al., J Clin Endocrin METAB 1982, 54: 1025-1027.
8. Optimization of a vehicle mixture for the transdermal delivery of melatonin using artificial neural networks and response surface method. Kandimalla et al., J Control Release 1999, 61: 71-82.
9. Melatonin and Parkinsonism. Papavasilou et al., JAMA 1972, 221: 88-89.
10. Effects of melatonin treatment in septic newborns. Gitto et al., Ped Res 2001, 50: 756-760.

CONFIDENTIAL

11. Utility of melatonin to treat surgical stress after major vascular surgery – a safety study. Kucukakin et al., J Pineal Res 2008, 44:426-431.

CONFIDENTIAL

3.1.5. GLUTATHIONE, TATIONIL ®

Introduction

Glutathione (GSH) is one of the major endogenous antioxidants produced by the cells in high concentration providing a prime position to neutralize free radicals and to maintain the cellular redox potential. GSH participates directly in the neutralization of free radicals and reactive oxygen species (ROS). Additionally, GSH improves the hepatic microcirculation by counteracting the ROS-mediated mechanisms of vasoconstriction and leukocyte adherence/vascular occlusion and by preventing detachment of sinusoidal endothelial cells. Moreover, studies have demonstrated that GSH released through the GSH transporter of hepatocytes may act as an endogenous defense system against Kupffer cell–mediated endothelium damage (1). Finally, GSH is crucial in the modulation of the immune system. Indeed glutathione is required for cells in modulating antigen presentation to lymphocytes, thereby influencing cytokine production like leukotrienes, prostaglandins and cytokines. By doing so, Glutathione is involved in the regulation of cell proliferation, apoptosis, DNA synthesis and protein synthesis, thus maintaining control of the immune response. It is well known from experimental models, as well as from data obtained from human allograft recipients, that antioxidants are consumed during reperfusion injury of transplanted grafts (2, 3).
Studies have indicated that GSH was able to react spontaneously with nearly all oxidants formed during inflammation (4). Shauer et al. has described an effective impact of GSH on rat liver models of ischemia reperfusion and transplantation with a significant reduction in ALT after reperfusion (5, 6). They clearly demonstrated the essential role of a preventive administration of GSH in protecting the parenchymal liver cells. Indeed, post-ischemic GSH treatment can only protect cells that are not already seriously damaged before the onset of reperfusion. This GSH-mediated cytoprotection seems to be attributable to an extracellular antioxidant mechanism as no evidence of increase of the GSH concentration in the hepatic cells after intravenous administration has been described (11). In humans, recent investigations have proven the therapeutic potential of GSH, in particular since it has a low toxicity in humans (7) and it is cost-effective. Chawla et al. has described a lower concentration of GSH in the plasma of cirrhotic patients (8). Thus, any intervention increasing hepatic or plasma GSH levels should convey protection against reperfusion injury (9). As shown recently in vitro, treatment of cold preserved livers with 2 or 4 mM GSH upon reperfusion prevented cell damage to hepatocytes in a model of cell-free rat liver perfusion (10).

Safety and adverse reaction

Overall, Glutathione and precursor (acetylcysteine) appears to have a low toxicity in humans. Currently, there are no known side effects of GSH (7). None adverse reactions have been reported, neither in clinical studies nor in case reports.
Interactions may occur between the different antioxidants. GSH maintains exogenous antioxidants such as Vitamins C and E in their reduced (active antioxidant) forms.

Contra-indication linked to the use of Tationil®

There is no specific contraindication linked to the use Tationil® in the multifactorial modulation.

CONFIDENTIAL

Product characteristics

Tationil 600® is manufactured by Teofarma Italy and will be distributed by a wholesale in Germany (= regular supplier of the pharmacy in UZ Leuven) . It is a white, crystalline powder freely soluble in water.

Packaging and storage

Each box contains 10x600mg of Glutathione together with 10x4ml distilled aqua for mixing.
It should be stored in airtight containers and protected from light.
In vitro studies have shown that reduced Glutathione (GSH) is oxidized to GSSG during storage at 4°C limiting the half-life to eight days. This can be prevented by lyophilization of GSH.

Dose and administration

Aebi et al. (7) has studied the pharmacokinetics of GSH in man after IV infusion. They have demonstrated that a high dose of IV GSH distributes safely in the extracellular compartment and is cleared from the circulation with a half-life of ±15 min.
It must be noted that the best dosage of Glutathione has not been clearly established for any use; most dose schedules have been empirically chosen based on animal data. Applied IV concentrations vary from 2.0 to 4.8 gram in total for an adult. For example, GSH has been reported in randomized studies to reduce the incidence of neurotoxicity induced by chemotherapy at a dose of 1.5 g/m^2 (10, 11, 12). IV Glutathione is also used to slow down the progression of Parkinson's disease. Most patients are given 1400 mg along with saline for ten minutes, three times a week. It has been shown to have a critical function as elimination of toxic compounds and by restoring the hepatic glutathione stores in case of acetaminophen intoxication. In that case, an approved 20 hour IV protocol for acetylcysteine –precursor of glutathione- treatment has been used in the UK since 1970s. This treatment protocol provides a total of 300 mg/Kg over 20 hours with an initial loading dose of 150 mg/kg IV over 15 to 60 minutes. This treatment period is often extended when patients have large ingestions of acetaminophen or elevated serum transaminase (15).
In this study, the IV dose of GSH has been determined according the experimental and clinical data that have proven profound effect without toxicological events. According the short half-life, 3 gr will be administrated 2-4 minutes just before the reperfusion.

References:

1. Neutrophil and Kupffer cell-induced oxidant stress and ischemia-reperfusion injury in rat liver. Jaeschke H et al., *Am J Physiol*. 1991;260:G355–362.
2. Primary graft nonfunction and Kupffer cell activation after liver transplantation from non-heart-beating donors in pigs. Monbaliu et al., Liver Transplant 2007; 13: 239-247.
3. Reperfusion injury, antioxidants and hemodynamics during orthotopic liver transplantation. Goode HF et al. Hepatology 1994; 19: 354.
4. Glutathione metabolism in activated human neutrophils: stimulation of glutathione synthesis and consumption of glutathione by reactive oxygen species. Bilzer M et al., *Eur J Clin Invest*. 1991;21:316–322.
5. Glutathione protects the rat liver against reperfusion injury after prolonged warm ischemia. Schauer et al., Ann Surg 2004; 239: 220-231.
6. Intravenous administration of glutathione protects parenchymal and non-parenchymal liver cells against reperfusion injury following rat liver transplantation. Schauer et al., World J Gastroenterol 2004; 10: 864-870.

CONFIDENTIAL

7. High-dose intravenous glutathione in man. Pharmacokinetics and effects on cysteine in plasma and urine. Aebi et al., Eur J Invest 1991; 21: 103-110.
8. Plasma cysteine, cystine, and glutathione in cirrhosis. Chawla et al., Gastroenterology 1984; 87: 770.
9. Plasma membrane and mitochondrial transport of hepatic reduced glutathione. Fernández-Checa JCet al. *Semin Liver Dis*. 1996; 16:147–158.
10. Glutathione protects the rat liver against reperfusion injury after hypothermic preservation. Bilzer M et al., *Gastroenterology*. 1999; 117:200–210.
11. Beneficial effects of extracellular glutathione against endotoxin-induced liver injury during ischemia and reperfusion. Liu P et al., *Circ Shock*. 1994;43:64–70.
12. Neuroprotective effect of reduced glutathione on cisplatin-based chemotherapy in advanced gastric cancer: a randomized double-blind placebo-controlled trial. Cascinu et al., J Clin Oncol 1995; 13: 26-32.
13. Neuroprotective effect of reduced glutathione on oxaliplatin-based chemotherapy in advanced colorectal cancer: a randomized, double-blind, placebo-controlled trial. Cascinu et al., J Clin Oncol 2002; 20: 3478-83.
14. Administration of reduced glutathione in FOLFOX4 adjuvant treatment for colorectal cancer: effect on oxaliplatin pharmacokinetics and neurotoxicity. Anti-Cancer Drugs 2009; 20: 396-402.
15. Treatment of paracetamol (acetaminophen) poisoning with N-acetylcysteine. Prescott LF, Park J, Ballantyne A, Adriaenssens P, Proudfoot AT. Lancet. 1977;2(8035):432.

CONFIDENTIAL

3.1.6. INFLIXIMAB, REMICADE ®

Introduction

TNF-α is a pleiotropic cytokine that induces cellular responses such as proliferation and production of inflammatory mediators. In the liver, TNF-α is involved in the pathophysiology of viral hepatitis, alcoholic liver disease, non-alcoholic fatty liver disease and IRI.

TNF-α is produced mainly by macrophages but also by a broad variety of other cell types including lymphoid cells and endothelial cells. TNF-α is primarily produced as a transmembrane protein but may be released in soluble trimeric form via proteolytic cleavage by the metalloprotease TNF-converting enzyme (TACE). TNF-α exerts its biological functions via interactions with cognate membrane receptors, TNF-R1 and TNF-R2. These signalling pathways interact in a complex network at several levels and activation of one pathway often depends on the inactivation of another pathway, suggesting that cells are capable of directing the TNF-α-induced signal toward the appropriate response. In normal liver TNF-R1 expression is slow, but high TNF-R1 expression occurs in hepatocytes, cholangiocytes, sinusoidal endothelium and inflammatory cells in disease states. Whereas TNF-R1 is efficiently activated by soluble TNF-α, TNF-R2 activation requires the binding of membrane-bound TNF-α (1).

TNF-α is a crucial mediator in hepatic reperfusion injury. During ischemia-reperfusion, Kupffer cells generate ROS subsequent to expression of MAP-kinase JNK, which in turn activates and enhances the secretion of TNF-α. TNF-α released may activate TNF-α receptors on hepatocytes to induce JNK and Inhibitor of κB (IKK) activation as well as ROS production. Whereas ROS promote hepatocytes cell death; IKK activation further enhances leukocyte infiltration in the liver (2).

TNF-α antagonists constitute a recent class of immunomodulators. Infliximab® is a chimeric human-murine monoclonal antibody that binds with high affinity to both soluble and transmembrane forms of TNF-α. Infliximab is well established in the treatment of rheumatoid arthritis (I.V. 3 mg/kg at 0, 2, and 6 weeks, followed by 3 mg/kg every 8 weeks thereafter; doses have ranged from 3-10 mg/kg repeated at 4- to 8-week intervals), psoriatric arthritis (I.V.: 5 mg/kg at 0,2, and 6 weeks, followed by 5 mg/kg every 8 weeks thereafter), ankylosing spondylitis (I.V.: 5 mg/kg at 0, 2, and 6 weeks, followed by 5 mg/kg every 6 weeks thereafter), inflammatory bowel diseases (I.V.: 5 mg/kg at 0, 2, and 6 weeks, followed by 5 mg/kg every 8 weeks thereafter; dose may be increased to 10 mg/kg in patients who respond but then lose their response), and several studies have demonstrated a high efficacy with rapid overall patient improvement, maintenance treatment, and decrease in rates of hospitalizations and surgical interventions (3).

Due to these promising results, experimental Infliximab treatment has been transferred to the field of organ transplantation. Experimental data have shown that inhibition of TNF-α signalling by TNF antiserum or genetic inactivation of TNF-R1 ameliorates hepatic reperfusion injury and prolongs recipient survival (4, 5). Moreover, TNF-α has been assessed in experimental studies after lung (6) and renal transplantations (7). TNF-α inhibition resulted in a reduction of inflammatory responses and prolonged graft survival. Clinically, anti-TNF-α monoclonal antibodies which bind directly to soluble TNF-α have been studied extensively in the treatment of patients with sepsis (8, 9). These studies have revealed that anti-cytokine administration usually as a single dose is safe but not particularly efficacious in the management of sepsis. TNF activity, however,

CONFIDENTIAL

peaks within hours of insult and usually precedes the initiation of anti-TNF-α therapy by hours or days in these clinical studies. The most efficacious use of these agents therefore appears to be in clinical scenarios permitting pre-treatment. Furthermore, clinical experience with TNF-α inhibitors after solid organ transplantation has revealed good results in terms of safety, reduction in toxicity and allograft survival (10). In some case reports, Infliximab has been shown to be a therapeutic option for refractory acute rejection after intestinal transplantation (11). Currently in Leuven, Infliximab at the dose of 3 mg/Kg is infused in patient during intestine transplantation at the same timing that foreseen in this protocol.

Safety and adverse reaction

Single doses up to 20 mg/kg have been administered without toxic effects.
Infliximab is contra-indicated in patients with a history of hypersensitivity, tuberculosis or other severe infections such as sepsis, abcesses and opportunistic infections. Moreover, it is not recommended to administrate Infliximab < 5 mg/kg in patients with severe heart failure.

Infliximab has been associated with acute infusion-related reactions, including anaphylactic shock and delayed hypersensitivity reactions. These reactions may occur during (within seconds) or within a few hours following infusion.

Antibodies to Infliximab may develop and have been associated with an increased frequency of infusion reactions. A low proportion of the infusion reactions were serious allergic reactions. An association between development of antibodies to Infliximab and reduced duration of response has also been observed. Concomitant administration of immunomodulators has been associated with lower incidence of antibodies to Infliximab and a reduction in the frequency of infusion reactions. The effect of concomitant immunomodulator therapy was more profound in episodically treated patients than in patients given maintenance therapy. In clinical studies using single and multiple Infliximab doses ranging from 1 to 20 mg/kg, antibodies to Infliximab were detected in 14% of patients with any immunosuppressant therapy, and in 24% of patients without immunosuppressant therapy.

Reactivation of hepatitis B has occurred in patients receiving Infliximab. However, in our institution, each patient who is a chronic carrier or suffering of an active infection are treated by immunoglobulines against HBV during and after the LTx.

Very rare cases of jaundice and non-infectious hepatitis, some with features of autoimmune hepatitis, have been observed in the post-marketing experience of Remicade®. Isolated cases of liver failure resulting in LTx or death have occurred.

There have been reports of pancytopenia, leucopenia, neutropenia and thrombocytopenia in patients receiving TNF-blockers, including Remicade®.
No interaction studies have been performed.

Contra-indication linked to the use of Remicade®

Patient already exposed to Remicade®, with a history of hypersensitivity, tuberculosis or other severe infections such as sepsis, abcesses and opportunistic infections and patients with severe heart failure will be excluded from the treatment group.

CONFIDENTIAL

Product characteristics

Remicade®, the antagonist of TNF-α is manufactured by Janssen Biologics. It is a sterile freeze-dried powder for solution for infusion.

Packaging and storage

The shelf-life is 3 years.

The Remicade® should be stored in a refrigerator (2°C - 8°C).

Each vial (rubber stopper and aluminium crimp) contains 100 mg of Infliximab. After reconstitution each ml contains 0,4 mg of Infliximab.

Remicade® is available in packs of 1, 2, 3, 4 or 5 vials.

The chemical and physical in use stability of the reconstituted solution has been demonstrated for 24 hours at 25°C. From a microbiological point of view, the product should be used as soon as possible but within 3 hours of reconstitution and dilution. If not used immediately, in use storage times and conditions prior to use are the responsibility of the user and should not be longer than 24 hours at 2 to 8°C.

Dose and administration

Since Infliximab is a human-derived monoclonal antibody, 3 hours of infusion are required in order to avoid allergic reactions.

Wen et al. (12) have determined evolution of the plasma level of TNF-α during orthotopic LTx. An increase of TNF-α plasma levels was observed in all patients after the reperfusion. TNF-α concentrations increased rapidly at the clampage of the vena portae, peaked at 90 minutes after the reperfusionand then decreased rapidly after 3 hours post-reperfusion until 24 hours after the operation.

In this study, the IV dose of Infliximab has been determined according to the experimental and clinical data that have proven profound effect without toxicological events (8, 9, 10). Pharmacokinetic studies have described a long half-life ranging from 11 to 14 days after the IV administration. Thus, 3 mg/kg of Infliximab will be administrated during 3 hours from the beginning of the anhepatic phase after administration of Antithrombin III.

References

1. Tumor necrosis factor signaling. Wajant et al., Cell Death Differ 2003 ; 10 :45-65.
2. Mechanisms of liver injury. TNF-α-induced liver injury: role of IKK, JNK, and ROS pathways. Schwabe and al., Am J Physiol Gastrointest Liver Physiol 2006; 290: 583-589.
3. Long-term outcome of treatment with infliximab in 614 patients with Crohn's disease: results from a single-center cohort. Schnitzler et al., Gut 2009; 58: 492-500.
4. Role of tumor necrosis factor-α in the pathophyiologic alterations after hepatic ischemia/reperfusion injury in the rats. Colletti et al., J Clin Invest 1990; 85: 1936-1943.
5. Tumor necrosis factor-α, but not Fas, mediates hepatocellular apoptosis in the murine ischemic liver. Rudiger et al., Gastroenterology 2002; 122: 202-210.

CONFIDENTIAL

6. Tumor necrosis factor-alfa in a porcine bronchial model of obliterative bronchiolitis. Alho et al., Transplantation 2003; 76: 516-523.
7. Inhibition of the effects of the TNF in renal allograft recipients using recombinant human dimeric tumor necrosis factor receptors. Eason et al., Transplantation 1995; 59: 300-305.
8. Hepatotoxicity of immunomodulating agents and the transplant situation. Kaplowitz et al., Drug-induced liver disease 2007; 662-681.
9. Review article: the role of tumor necrosis factor in renal ischemia-reperfusion injury. Donnahoo et al., The Journal of Urology 1999, 162: 196-203.
10. New monoclonal antibodies in renal transplantation. Vincenti et al., Minerva Urol Nefrol 2003; 55: 57-66.
11. Intestinal transplantation: evolution in immunosuppression protocols. Pirenne et al., Curr Opin Organ Transplant 2009; 14: 250-255.
12. Plasma levels of tumor necrotic factor-α and IL-6, -8 during orthotopic liver transplantation and their relations to post-operative pulmonary complication. Wen et al., Hepatobiliary Pancreat Dis Int 2004; 3: 38-41.

CONFIDENTIAL

3.1.7. α-TOCOPHEROL, VITAMIN E SUSPENSION 100 mg/mL®

Introduction

Vitamin E is the collective name for a group of fat-soluble compounds that are active throughout the body. Vitamin E includes 4 Tocopherols and 4 Tocotrienols and naturally occurs in 8 chemical forms. α-Tocopherol has the highest Vitamin E activity and is the only formulation that is recognized to meet human requirements. It has been claimed that α-Tocopherol is the most important lipid-soluble free radical scavenger and antioxidant (1). It protects cell membranes from oxidation by reacting with lipid radicals, such as ROS produced in the lipid peroxidation chain reaction. In addition, Vitamin E is involved in immune function. It has been shown that α-Tocopherol was also implied in cell signalling, regulation of gene expression and other metabolic processes (1). α-Tocopherol inhibits the activity of protein kinase C, increases the release of prostacyclin from the endothelium, which in turn, dilates blood vessels and inhibits platelet aggregation. Finally, α-Tocopherol decreases the adhesion of inflammatory cells to the endothelial cell lining (1).

Oxidised α-tocopheroxyl radicals produced in this process may be recycled back to the active reduced form through reduction by other antioxidants, such as Vitamin C, Selenium, Vitamin B3 and GSH. Indeed, Vitamin C is required to keep Vitamin E in its metabolically active form; GSH is required to keep Vitamin C in its active form; and Selenium and Vitamin B3 are required to keep Glutathione in its active form. Moreover, α-Tocopherol may positively influence the total GSH content of the cells during the reperfusion period by blocking its decrease in the cells.

It is well known from experimental models, as well as from data obtained from human allograft recipients, that antioxidant vitamins are consumed during reperfusion injury of transplanted grafts (2, 3, 4). Moreover, antioxidant status and antioxidant vitamin levels in patients undergoing organ transplantation have shown that the circulating levels of GSH, Vitamins C and E were significantly lower in these patients before transplantation than in normal controls (1).

It has been described that lipid peroxidation observed after reperfusion can be effectively prevented with antioxidant vitamins, both in animal models (5) and in patients (6, 7). Moreover, α-Tocopherol alone has proven highly effective in reducing IRI in various models and clinical settings. Oda et al. described a decrease in the severity of reperfusion injury, an improvement of short-term allograft function, and survival in a rat model of pancreas transplantation (8). The same observations were demonstrated by Hower et al. in human kidney transplantation (7). Bartels et al. indicated that preoperative parenteral administration of Vitamin E in liver surgery was safe and that this treatment had beneficial effects by reducing the impact of IRI (9).

α-Tocopherol is usually administered per os, no clinical grade preparations of pure α-Tocopherol are known for IV administration in humans. However, α-Tocopherol is added to many multi-ingredient preparations, including those that are administered IV (e.g. Cernevit®).

The oral supplementation hours before acute episodes of oxidative stress may be less effective (10). This might be due to the fact that oral Vitamin E is delivered to the liver

CONFIDENTIAL

via chylomicrons and secreted in VLDL/LDL into the blood. Ferslew et al. (11) described that α-Tocopherol first appeared in the plasma in 2-4 hours after oral administration and peaked at 5-14 hours when administering 800 mg α-Tocopherol to healthy volunteers. The disappearance from the plasma was achieved with a half-life of 53 hours after administration. Moreover, short-term parenteral administration was shown to be superior to enriching endothelial cells with Vitamin E (10).

Safety and adverse reaction

This major antioxidant vitamin has excellent safety data in doses that far exceed the doses required to reach adequate antioxidant protection in vivo. Regarding controlled, double-blinded studies of Vitamin E toxicity in humans, several reports confirm that Vitamin E has very low toxicity and no consistent adverse events have been reported (12, 13). However, Vitamin E at high intakes can affect the coagulation if Vitamin K deficiency is also present. Clearly, Vitamin E must not be given in case of anticoagulant therapy. Alternatively, administration of Vitamin E can be accompanied by concomitant administration of Vitamin K.

Contra-indication linked to the use of Vitamin E suspension ®

Patient requiring an anticoagulation that functions as a Vitamin K antagonist perioperatively will be excluded from the treatment group.

Product characteristics

Vitamin E suspension ®is produced by Cambridge Laboratories. The capsules must be orally administrated.

Packaging and storage

Vitamin E suspension ®should be stored below 25°C (do not freeze), protected from light. The product is for single use in one patient only.

Dose and administration

Lassnig et al. (13) have studied multiple parenteral Vitamin E infusion in patients undergoing elective cardiac surgery. A total dose of 1200 IU Vitamin E was administered at four points in time: two infusions before the start of surgery and one infusion each on days 1 and 2 after surgery. Based on pharmacokinetic data the dose was considered sufficient to maintain Vitamin E concentrations during surgery. Since no protective effect of Vitamin E supplementation was observed, Lassnigg et al. suggested that the supply of Vitamin E was inadequate and/or that not enough Vitamin E was incorporated into the target cells. Based on these findings and investigations, Bartels et al. (9) decided to administer 1800 IU (1620 mg) the day before the liver surgery and compared to a placebo group the extent of IRI. After three infusions of Vitamin E, there was a significant fivefold increase in the plasma concentration; half of this increase remained about 12 h later, prior to surgery. After reperfusion, the Vitamin E concentration was significantly decreased in both groups. It is likely that, due to severe oxidative stress, the need for and consumption of, α-Tocopherol was great. Six days after surgery the treated group still had plasma Vitamin E concentrations comparable to the baseline value, whereas the placebo group still had significantly reduced plasma Vitamin E concentrations. They have shown that the preoperative parenteral administration of 1800 IU of α-Tocopherol was safe, not only leads to a marked increase of plasma concentration but also prevents Vitamin E depletion after IRI.

CONFIDENTIAL

Currently, no solutions containing Vitamin E alone are available for human use. Therefore, 5 mL of Vitamin E suspension ®will be administrated orally at the ward before transport to the operative theatre. This time course is adequate according the pharmacokinetic data of Vitamin E after oral administration.

References

1. Rationale for the use of antioxidant vitamins in clinical organ transplantation. Lehr et al., Transplantation 1996; 62: 1197-1199.

2. Inhibition of free radical generation and improved survival by protection of the hepatic microvascular endothelium by targeted erythrocytes in orthotopic liver transplantation. Rao PN et al. Transplantation 1990; 49: 1055.

3. Reperfusion injury, antioxidants and hemodynamics during orthotopic liver transplantation. Goode HF et al. Hepatology 1994; 19: 354.

4. Primary graft nonfunction and Kupffer cell activation after liver transplantation from non-heart-beating donors in pigs. Monbaliu et al., Liver Transplant 2007; 13: 239-247.

5. Effect of alpha tocopherol on the prevention of reperfusion injury caused by free oxygen radicals in the canine kidney autotransplantation model. Demirbas A et al. Transplant Proc 1993; 25: 2274.

6. A multivitamin infusion prevents lipid peroxidation and improves transplantation performance. Rabl H et al. Kidney Int 1993; 43: 912.

7. Assessment of oxygen radicals during human kidney transplantation: effect of radical scavenger. Hower R et al. Transpl Int Transpl Int 1996;9 Suppl 1:S479-82.

8. Role of oxygen radicals and synergistic effect of superoxide dismutase and catalase on ischemia-reperfusion injury of the rat pancreas. Oda T et al. Transplant Proc 1992; 24: 797.

9. Pilot study on the effect of parenteral vitamin E on ischemia and reperfusion induced liver injury: a double-blind, randomized, placebo-controlled trial. Bartels et al., Clin Nutrit 2004; 23: 1360-1370.

10. Short-term parenteral application of tocopherol leads to increased concentration in plasma and tissue of the rat. Engelhart et al., Free radic res 1998; 29: 421-426

11. Pharmacokinetics and bioavailability of the RRR and all racemic stereoisomers of alpha-tocopherol in humans after single oral administration. Ferslew et al., J Clin Pharmacol 1993; 33: 84-88.

12. Safety of antioxidant vitamins and B-carotene. Diplock et al., Am J Clin Nutr 1995; 62: 1510-1516.

13. Influence of intravenous vitamin E supplementation in cardiac surgery on oxidative stress: a double-blinded, randomized, controlled study. Br J Anaesth 2003; 90: 148-154.

CONFIDENTIAL

3.1.8. APOTRANSFERRIN

Introduction

Transferrin is the major iron carrier protein in human plasma and extracellular space in tissue of a healthy individual. Transferrin iron saturation is 20-35% and redox-active non-transferrin-bound iron cannot be detected in the serum.

Several studies have indicated circumstances such as malignacies associated with an increase in the serum total iron content, which often exceeds the iron binding capacity of transferrin and results in the appearance of non-transferrin-bound iron in the serum of patients (1, 2).

The Rationale to the attempt binding NTBI in the patients by administering Apotransferrin is the prevention of iron-induced toxicity, which is thought to depend on iron-catalyzed formation of hydroxyl radicals (3). NTBI is effectively taken up by parenchymal cells, particularly in the liver and there are several lines of evidence suggesting that NTBI is toxic to liver cells (4). On the other hand, NTBI in the sera of leukaemia patients has been shown to induce lipid peroxidation and it could thus damage cells even without uptake into the cells (2). Ferric iron causes cytotoxicity after a few hours in liver cell cultures (5) and it also rapidly impairs the phagocytic activity of polymorphonuclear leukocytes (6). Another possible benefit of the binding of NTBI by Apotransferrin could be the prevention of the growth of opportunistic bacteria and fungi. Practically all micro-organisms are dependent on iron for growth. In normal plasma, transferrin keeps the level of free iron far too low to sustain the growth of micro-organisms and only virulent bacterial species have developed mechanisms to acquire iron directly from transferrin (7). The increased level of free iron may thus predispose the neutropenic and immunosuppressed patients to septic infections by opportunistic bacteria and fungi that are dependent on NTBI for growth (1). Another possible benefit is that Apotransferrin binds NTBI into a physiological form that can be utilized by the recovering bone marrow.

A potential alternative for Apotransferrin as an iron-chelating agent, desferrioxamine has been extensively studied in chronic iron overload diseases. Unfortunately, it has been shown that some of the NTBI is not effectively chelated with desferrioxamine (8) and the presence of NTBI in patient sera without full transferrin saturation has been reported (9). Moreover, this low-molecular-weight iron chelator displays dose-related toxicity (10) and may increase the risk of bacterial and fungal infections (11).

Safety and adverse reaction

Apotransferrin is purified from human plasma. Sahlstedt et al. (12) have demonstrated that a single dose of Apotransferrin was effective and safe on the serum iron binding capacity. They have shown that this effect was temporarily for variable periods from hours to days. Parkkinen et al. have described in a dose-finding study the highest dose level for the prevention of appearance of NTBI in patients with myeloablation. A total dose of 1040 mg/kg (115 mg/Kg/day for 9 days) was given to the patients without any related serious adverse event (13).

Contra-indication linked to the use of Apotransferrin

There is no specific contraindication linked to the use Apotransferrin in the multifactorial modulation.

CONFIDENTIAL

Product characteristics

Human Apotransferrin is manufactured by Sanquin Blood Supply Foundation. The product has no marketing authorisation. However, Apotransferrin is currently used in few patients with a congenital Apotransferrin deficiency worldwide and no toxic side effects have been reported so far (personal communication Sanquin). Apotransferrin consists of purified and iron-depleted protein prepared from human plasma. The product is supplied as a colourless to slightly yellowish, sterile and pyrogen-free liquid solution.

Packaging and storage

Apotransferrin is supplied as solution in a filling size of 40 ml containing 2 g of apotransferrin (50 g/l). Apotransferrin is supplied in hydrolytic glass vials with a bromobutyl rubber stopper, an aluminium cap and a flip-off seal.
The primary container consists of colourless and transparent vials of type II glass (50 ml container).
Apotransferrin should be stored at a temperature of 2-8 ˚C and protected from light.

Dose and administration

170 mg/Kg of Apotransferrin® will be infused during 3 hours from the beginning of the anhepatic phase.

References:

1. Non-transferrin bound iron and neutropenia after cytotoxic chemotherapy. Harrison et al., J Clin Pathol 1994; 47: 350-352.
2. Presence of iron catalytic for free radical reactions in patients undergoing chemotherapy: implications for therapeutic management. Carmine et al., Cancer letters 1995; 94: 219-226.
3. Role of free radicals and catalytic metal ions in human disease: an overview. Halliwell et al. 1990, Method in enzymologie; 186: 1-85.
4. Non-transferrin bound iron and cellular toxicity. Anderson et al. 1999; J Gastroenterol Hepatol; 14: 105-108.
5. Oxidative stress and cytotoxicity induced by ferric-nitrilotriacetate in HepG2 cells that express cytochrome P450 2^{E}1. Sakurai et al., Molecular Pharmacology 1998; 54: 1024-1035.
6. Effect of iron in the presence of various ligands on the phagocytic and metabolic activity of human polymorphonuclear leukocytes. Van asbeck et al., J Immunol 1984; 132: 851-856.
7. Iron metabolism in pathogenic bacteria. Ratledge et al., Annual review of microbiology 2000; 54: 881-941.
8. Desferrioxamine-chelatable iron, a component of serum non-transferrin-bound-iron, used for assessing chelation therapy. Breuer et al., Blood 2001; 97: 792-798.
9. Non-transferrin bound iron in plasma from hemochromatosis patients: effect of phlebotomy therapy. Blood 1988; 72: 1416-1419.
10. Kinetics of removal and reappearances of non-transferrin-bound plasma iron with deferoxamine therapy. Porter et al., Blood; 88: 705-713.
11. Iron withholding: a defense against infection and neoplasia. Weiberg et al., Physiological reviews 1984; 64: 65-102.
12. Effective binding of free iron by a single intravenous dose of human apotransferrin in haematological stem cell transplant patient. Sahlstedt et al., Br J Haemato 2002; 119: 547-553.

CONFIDENTIAL

13. Effect of repeated apotransferrin administrations on serum iron parameters in patients undergoing myeloablative conditioning and allogenic stem cell transplantation. Parkkinen et al., Br J Haemato 2006; 135: 228-234.

CONFIDENTIAL

3.1.9. EPOPROSTENOL, FLOLAN®

Introduction

At early stages of inflammation the endothelium modifies its normal homeostasis and may lose the ability to synthesize NO and prostacyclin (PGI2). As a result, vasoconstriction takes place besides a prothrombotic phenotype favoring the expression of tissue factor and triggering the coagulation response. Prostacyclin is synthesized by all endothelial cells and in particular in the liver. PGI2, derived from arachidonic acid is a well-known vasodilatator and exerts anti-platelet aggregation properties (1). Additionally, PGI2 reduces leukocyte activation and adhesion. Furthermore, PGI2 regulates TNF-α synthesis and tissue factor expression in macrophages (1). Finally, PGI2 has cytoprotective effects and an antioxidant action. The underlying mechanism acts by a cyclic Adenosyl Mono-Phosphate (cAMP) dependant pathway (1).

The protective effects of the prostaglandin on liver necrosis were first reported by Stachura et al. (2). Since then, PGI2-mediated reduction of microcirculatory disorders and tissue injury has been proven as highly effective in various models of hepatic ischemia-reperfusion (3-4). In clinic, PGI2 was originally chosen for lung preservation because it allowed a more even distribution of cold perfusion. Its use has then been extended to other organs. Klein et al. has demonstrated a significant impact of the PGI2 in preconditioning the graft prior to the LTx (5). Indeed, an injection of 500 µg PGI2 in the donor has shown an improvement of the IRI due to the prevention of thrombosis and vasospasm after cold storage. Moreover, Muhlbacher et al. (6) has demonstrated that pretreatment with PGI2 (200 µg) to organ donors has also an impact on the kidney. He observed a reduction of the number of post-transplant dialyses and hospital care days in patients undergoing renal transplantation. Finally, Pirenne et al. demonstrated that donor treatment with PGI2 for better flushing and preserving peribiliary vascular plexus and biliary mucosa offers protection from biliary strictures (7).

Safety and adverse reaction

Flolan®, the analogue of Epoprostenol is contraindicated in patients with known hypersensitivity to the drug and in patients with congestive heart failure arising from severe left ventricular dysfunction. Due to its vasodilatator effect, hypotension may occur during IV Flolan infusion.

Epoprostenol is a potent inhibitor of platelet aggregation. Therefore, an increased risk for haemorrhagic complications should be considered. On the other hand, Epoprostenol is not considered as a conventional anticoagulant.

Facial flushing, decreased in platelet count and tachycardia have been commonly reported.

The cardiovascular effects during infusion disappear within 30 minutes of the end of administration. Effects on platelets have been found to disappear within 2 hours of discontinuing the infusion.

Its metabolites are inactive and excreted in the urine.

Patients on Digoxin have shown elevations of Digoxin concentrations after initiation of therapy with Epoprostenol. This may be clinically relevant in patients prone to Digoxin toxicity. Monitoring of Digoxin levels is therefore advisable until Digoxin levels are clinically stable in patients receiving treatment with Epoprostenol and Digoxin.

CONFIDENTIAL

Contra-indication linked to the use of Flolan®

Patient with a congestive heart failure arising from severe left ventricular dysfunction will be excluded from the treatment group.

Product characteristics

Flolan® is manufactured by GlaxoSmithKline. It is a sterile freeze-dried powder for solution for infusion. It requires special handling including a constant controlled temperature and protection from light. Pharmacokinetic studies have described a short half-life ranging from 3 to 5 minutes after the IV administration.
Glycine and Mannitol are provided as excipient.

Packaging and storage

The commercial packing of Flolan® consists of:

- A single 0.5 mg vial of freeze-dried powder in glass vials with synthetic butyl rubber plugs and aluminium collars.
- Single vial of diluents.

GlaxoSmithKline Glycine Buffer Diluent contains no preservative, consequently a vial should be used once only and then discarded.
The shelf-life is about 3 years. Flolan® should be stored below 25°C (do not freeze) and the unopened vial in an outer carton to protect from light and moisture. When reconstituted with GlaxoSmithKline Glycine Buffer Diluent and diluted with physiological saline as instructed, freshly prepared Flolan® solutions should be used within 12 hours at 25°C.

Dose and administration

Flolan® is not to be used for bolus administration and it must be reconstituted only with the specific sterile GlaxoSmithKline Glycine Buffer Diluent.
To avoid any side effects in the recipient, an ex-situ administration of Flolan® has been chosen. Flolan® will be administrated directly through the vena porta during the bench table. After reconstitution, 50 cc of Flolan® will be added to a liter of preservation solution. 500 cc will be flushed through both the vena porta. The IV dose of Flolan® has been determined according our clinical data. In case of DCD procedure with liver and lung procurement, we routinely use a preconditioning with 65 cc of Flolan®. 38 cc of reconstituted Flolan® are diluted in the lung cold perfusion whereas 7 cc are directly administrated through the pulmonary artery before the cooling of the lungs. Moreover 20 cc are diluted in the abdominal cold pre-flush solution.

<u>References:</u>

1. Ischemia and reperfusion: from mechanism to translation. Eltzschig and Eckle, Nature medicine 2011; 11: 1391.
2. Prostaglandin protection of carbon tetrachloride-induced liver cell necrosis in the rat. Stachura et al., Gastroenterology 1981; 81: 211-217.
3. Hepatoprotection by a PGI2 analogue in complete warm ischemia of the pig liver. Prostanoid release from the reperfused liver. Kim et al., Transplantation 1994; 58: 875-879.
4. Efficacy of prostaglandine I2 analog on liver grafts subjected to 30 minutes of warm ischemia. Kishida et al., Surg Today 1997; 27: 1056-1060.

CONFIDENTIAL

5. Preconditioning of donor livers with prostaglandin I2 before retrieval decreases hepatocellular ischemia-reperfusion injury. Klein et al., Transplantation 1999; 67: 1128-1132.
6. Improved renal graft function after prostacyclin pretreatment. Muhlbacher et al., Transplant Proc 1987; 19: 4162-4163.
7. Biliary strictures after liver transplantation: risk factors and prevention by donor treatment with epoprostenol. Pirenne et al., Transplant Proc 2009; 41: 3399-3402.

3.2 CONTRA-INDICATION TO USE THE COMBINED DRUG APPROACH

3.2.1. ANTI-THROMBIN III (Atenativ®)

Anti-thrombin III (Atenativ®) replacement during administration of heparin in therapeutic dosage increases the risk of bleeding. The effect of anti-thrombin III (Atenativ®) is enhanced by heparin. The half-life of anti-thrombin III (Atenativ®) may be considerably decreased with concomitant heparin treatment due to accelerated anti-thrombin III (Atenativ®) turnover (1.5 days).
Hypersensitivity or allergic reactions (which may include angioedema, burning and stinging at the infusion site, chills, flushing, generalized urticaria, headache, hives, hypotension, lethargy, nausea, restlessness, tachycardia, tightness of the chest, tingling, vomiting, wheezing) have been observed infrequently in conjunction with the use of anti-thrombin III (Atenativ®) concentrates and may in some cases progress to severe anaphylaxis (including shock). On rare occasions, fever has been observed.

- ⇨ Patient requiring **therapeutic dose of heparin pre-operatively** will be excluded from the treatment group.
- ⇨ History of **hypersensitivity** to anti-thrombin III (Atenativ®) and /or drugs with a similar chemical structure will be excluded from the treatment group.

3.2.2. C1-INHIBITOR (Cetor®/Cinryze®)

Hypersensitivity or allergic reactions (which may include angioedema, burning and stinging at the infusion site, chills, flushing, generalized urticaria, headache, hives, hypotension, lethargy, nausea, restlessness, tachycardia, tightness of the chest, tingling, vomiting, wheezing) could be observed with C1-inhibitor (Cetor®/Cinryze®) and may in some cases progress to severe anaphylaxis (including shock).

- ⇨ History of **hypersensitivity** to C1-inhibitor (Cetor®/Cinryze®) and /or drugs with a similar chemical structure will be excluded from the treatment group.

3.2.3. ERYTHROPOÏETIN BETA (Neorecormon®)

Safety data arising from clinical studies suggest that a high single IV dose of rhEPO (Neorecormon®) is safe regarding acute as well as chronic adverse effects including vascular thrombosis, seizure and raise in blood pressure, Hb levels, platelets and hematocrit. However, as patients with a history of seizure and a level of Hb higher than 13 gr/dl were excluded in a few studies.

- ⇨ Patient with a **pre-operative level of Hb higher than 13 g/dl** will be excluded from the treatment group.

CONFIDENTIAL

- ⇨ Patient with a history of **seizure**, **poorly controlled arterial hypertension**, **myocardial infarction** or **stroke** in the month preceding the liver transplantation, and **venous thromboembolic disease** will be excluded from the treatment group.
- ⇨ Patients with **unstable angina pectoris** will be excluded from the treatment group.
- ⇨ History of **hypersensitivity** to rhEPO (Neorecormon®) and /or drugs with a similar chemical structure will be excluded from the treatment group.

3.2.4. MELATONIN (Circadin®)

Hypersensitivity or allergic reactions could be observed with melatonin (Circadin®).

- ⇨ History of **hypersensitivity** to melatonin (Circadin®) and /or drugs with a similar chemical structure will be excluded from the treatment group.

3.2.5. GLUTATHIONE (Tationil®)

There is no specific contraindication linked to the use glutathione (Tationil®) in the multifactorial modulation.

Overall, Glutathione and precursor (acetylcysteine) appears to have a low toxicity in humans. Currently, there are no known side effects of GSH. None adverse reactions have been reported, neither in clinical studies nor in case reports.

3.2.6. INFLIXIMAB (Remicade®)

- ⇨ Patient **already exposed to Infliximab** (Remicade®) will be excluded from the treatment group.
- ⇨ History of **hypersensitivity** to infliximab (Remicade®) and /or drugs with a similar chemical structure and/or murine proteins will be excluded from the treatment group.
- ⇨ Patients with **severe infections** such as sepsis, abcesses and **opportunistic infections** will be excluded from the treatment group.
- ⇨ Patients with **severe heart failure** will be excluded from the treatment group.

3.2.7. TOCOPHEROL (vitamin e suspension 100 mg/mL®)

Vitamin E has been reported to increase the risk of thrombosis in patients taking oestrogens. This finding has not been confirmed but should be borne in mind when selecting patients for treatment, in particular women taking oral contraceptives containing oestrogens. Moreover, Vitamin E at high intakes can affect the coagulation if

CONFIDENTIAL

Vitamin K deficiency is also present. Clearly, Vitamin E must not be given in case of anticoagulant therapy. Alternatively, administration of Vitamin E can be accompanied by concomitant administration of Vitamin K.

- ⇨ Patient requiring an anticoagulation that functions as a **Vitamin K antagonist preoperatively** will be excluded from the treatment group.
- ⇨ History of **hypersensitivity** to tocopferol (vitamin e suspension 100 mg/mL®) and /or drugs with a similar chemical structure will be excluded from the treatment group.

3.2.8. APOTRANSFERRIN

There is no specific contraindication linked to the use Apotransferrin in the multifactorial modulation.

3.2.9. EPOPROSTENOL (Flolan®)

Due to its vasodilatator effect, hypotension may occur during IV epoprostenol infusion. Moreover, epoprostenol (Flolan®) is a potent inhibitor of platelet aggregation. Therefore, an increased risk for haemorrhagic complications should be considered.

- ⇨ Patient with a **congestive heart failure** arising from severe left ventricular dysfunction will be excluded from the treatment group.
- ⇨ Patient requiring **therapeutic dose of heparin pre-operatively** will be excluded from the treatment group.
- ⇨ History of **hypersensitivity** to epoprostenol (Flolan®) and /or drugs with a similar chemical structure will be excluded from the treatment group.

3.3 RATIONALE FOR THE CHOICE OF DOSES

For each component of the multifactorial modulation approach, the lowest but still efficient and non-toxic dose has been chosen. A detailed listing is proved for every component.

3.3.1. ANTI-THROMBIN III (Atenativ®)

Anti-thrombin III (Atenativ®) has already been used safely in clinic. Hoffmann et al. has characterized a significant improvement of renal allograft reperfusion by a single-shot of anti-thrombin III (Atenativ®) (4000 IU) application during human kidney transplantation in a randomized controlled trial (1). Further, the same group has described a reduction of reperfusion injury pancreatitis and a prevention of graft thrombosis with a single-shot of anti-thrombin III (Atenativ®) (3000 IU) in human pancreas-kidney transplantation (2). At this recommended dose, Fertmann et al. (2) did not observe perioperative anti-thrombin III (Atenativ®)-mediated changes on standard coagulation tests (partial thromboplastin time, thromboplastin time, platelet counts), on the perioperative bleeding rates and on the number of packed red blood cell concentrates given to the patient. Currently, a multi-centric protocol for clinical phase III trial testing pre-reperfusion anti-thrombin III (Atenativ®) (3000 UI) application in combination with acetylcysteine is ongoing for simultaneous kidney and pancreas transplantation.

According these clinical data that have proven profound effect without toxicological events, a single IV dose of 3000 IU of ATIII (Atenavit®) has been chosen.

<u>*References*</u>

1. High dose antithrombine therapy reduces IRI and improves graft function during human allogenic kidney transplantation: final results of a randomized controlled clinical trial. Hoffmann et al.,Transplantation 2002; 74, 394.

2. Single-shot antithrombin in human pancreas-kidney transplantation: reduction of reperfusion pancreatitis and prevention of graft thrombosis. Fertmann et al., Transplant International 2005; 18: 40.

3.3.2. C1-INHIBITOR (Cetor®/Cinryze®)

Besides the routine clinical practice use of C1-inhibitor (Cetor®/Cinryze®) in angioedema (1000 U), clinical trials have been performed to test the protective effect of C1-inhibitor (Cetor®) during ischemia reperfusion injury. Different doses were used. Struber et al. focused on the decrease of vascular leakage syndrome after lung transplantation by using 26.900 U of C1-inhibitor (Cetor®) (1). Bauernschmitt et al. (2000 U) (2), Thielmann et al. (4800 U)(3) and Fattouch et al. (1000 U) (4) have all described a significant positive impact of C1-inhibitor (Cetor®) on the cardiac function during coronary surgical reperfusion. Stieh et al. has observed the same findings with a post-operative administration of C1-inhibitor (Cetor®) at the dose of 56000 U in open heart surgery. Toxicity of C1-inhibitor (Cetor®/Cinryze®) is very low. No side effects were observed in those clinical trials (1, 2, 3, 4). No complications are expected in patients either with a liver dysfunction or during the liver transplantation.

According these clinical data that have proven profound effect without toxicological events, a single IV dose of 1000 U of C1-inhibitor (Cetor®/Cinryze®) has been chosen.

CONFIDENTIAL

<u>*References*</u>

1. C1-esterase inhibitor in graft failure after lung transplantation. Struber et al., Intensive care med 1999; 25: 1315-1318.

2. Rescue therapy with C1-esterase inhibitor concentrate after emergency coronary surgery for failed PTCA. Bauernschmitt et al., Intensive care medicine 1998; 24: 635-638.

3. Administration of C1-esterase inhibitor during emergency coronary artery bypass surgery in acute ST-elevation myocardial infarction. Thielmann et al., Eur J Cardio Thorac Surg 2006; 285-293.

4. Beneficial effects of C1-esterase inhibitor in ST-elevation myocardial infarction in patients who underwent surgical reperfusion: a randomized double-blind study. Fattouch et al., Eur J Cariothorac Surg 2007; 32: 326.

3.3.3. RECOMBINANT HUMAN ERYTHROPOÏETIN BETA (Neorecormon®)

A growing number of clinical studies are testing the tissue protection effect afforded by rhEPO beta (Neorecormon®) in various acute clinical settings, including neuroprotection and cardioprotection. Ehrenreich et al (1) has used EPO beta at a total dose of 100.000 IU for the first 3 days after an acute cerebral stroke, noting that this was safe and well tolerated. Lipsic et al (2) has demonstrated that a single bolus injection of EPO at a dose of 60.000 IU in patient with an acute myocardial infarction was not associated to any significant increase in hemoglobin (Hb). Mocini et al (3) has described that a single injection of EPO at a dose of 40.000 IU before cardiac surgery has no significant impact on the cardiac function and on the erythropoiesis. Belonje et al (4) has demonstrated in a safety pilot assessment that 60.000 IU of EPO in patients with an acute myocardial infarction was not associated with a raise in blood pressure, an increase in Hb and thrombocytes or any adverse events like vascular thrombosis or seizure. In elective liver surgery, Mosato Kato et al (5) has indicated that a two time injection of 30.000 IU EPO rather than one single bolus of 60.000 IU has a stronger inhibitory effect on IRI following the pringle maneuver during liver surgery. Moreover, these doses and their timing of administration did not affect blood cell count, hematocrit and platelets.

Considering the results of clinical studies in different organs, it is suggested to adapt the dose of EPO and its timing of administration in order to reach an optimal protective effect. This was confirmed by Ramakrishnan et al. (6). They have described the importance of doses, dosage regimens and routes of administration in physiologically mechanistic PK/PD model. Pharmacokinetic investigations in healthy volunteers show that the half-life of IV administrated EPO beta is between 3 and 12 hours.

Notably, the receptors mediating the non-erythropoietic effects of EPO differ from the one responsible for hematopoiesis. The tissue-protective receptor exhibits a lower affinity for EPO. It is a heteromer consisting of EPO receptor monomers in association with the common receptor. This heteromeric receptor is expressed immediately following injury, whereas EPO production is delayed providing a window of opportunity for therapeutic intervention. Thus early administration of EPO can dramatically reduce the deleterious components of the local inflammatory cascade. However, a larger dose is required to trigger the protective effect as the affinity for the heteromer is lower than the hematopoietic receptor. Thus, to effectively treat injured tissue using exogenous EPO, high parenteral doses are required (7).

CONFIDENTIAL

Considering the distribution profile after IV injection, the half-life of EPO-beta (3-12h), the potential elimination of the administrated medication by blood loss during the procedure, the need to keep a high concentration for the post-reperfusion phase and the absence of toxicological events, a first dose of 30.000 IU will be administrated 13-15 minutes before the reperfusion of the liver over 2 minutes followed by a second dose of 30.000 IU of EPO-beta 6 hours after the reperfusion.

<u>*References*</u>

1. Ehrenreich et al, Molecular medicine 2002; 8: 495-505. EPO therapy for acute stroke is both safe and beneficial.

2. Lipsic et al, J Am Coll Cardiol 2006; 48: 2161-2167. Protective effect of EPO in cardiac ischemia: from bench to bedside.

3. Mocini et al, Perfusion 2008; 23: 187-192. Endogenous EPO and a single bolus of 40.000 IU do not protect the heart from IRI during extracorporeal circulation for cardiac surgery.

4. Belonje et al, Am Heart J 2008; 155: 817-822. Effects of EPO after an acute myocardial infarction: Rationale and study design of a prospective, randomized, clinical trial.

5. Mosato Kato et al, World Journal of Gastroenterology 2010; 16: 4838-4845. EPO ameliorates early ischemia-reperfusion injury following the pringle maneuver.

6. Ramakrishnan et al, J Clin Pharmacol 2004; 44: 991-1002. PK and PD modeling of recombinant human EPO after a single and multiple doses in healthy volunteers.

7. Brines et al, Blood Purif 2010; 29: 86-92. The therapeutic potential of EPO for tissue protection: a tale of two receptors.

3.3.4. MELATONIN (Circadin®)

The antioxidative effects of melatonin require concentrations that are much higher than endogenously produced plasma concentrations. On the other hand, a very low concentration of exogenous Melatonin (> 0.3 mg) produces already supra-physiological levels in humans. Cost and toxicity of melatonin melatonin is remarkably low. In the United States, the Food and Drug Administration (FDA) has designated melatonin as an orphan product sold as a dietetic complement. No significant adverse effects were observed following administration of high doses (3-6.6 g/day) in humans for 15 to 35 days (1). High doses of oral melatonin have been used with success in babies with sepsis syndromes without toxicological effect (20 mg) (2). IV infusion in doses between 10 and 60 mg during abdominal aortic-aneurysm repair has already been shown to be without immediate toxic reaction (3). In this study, the oral dose of melatonin (Circadin®) has been determined according the clinical data that have proven profound effect without toxicological events. Thus 6 mg of melatonin (Circadin®) will be administrated orally at the ward just before transfer to the operative theatre. This time course is adequate according the pharmacokinetic data of melatonin after oral administration (4).

<u>*References*</u>

1. Melatonin and Parkinsonism. Papavasilou et al., JAMA 1972, 221: 88-89.

2. Effects of melatonin treatment in septic newborns. Gitto et al., Ped Res 2001, 50: 756-760.

CONFIDENTIAL

3. Utility of melatonin to treat surgical stress after major vascular surgery – a safety study. Kucukakin et al., J Pineal Res 2008, 44:426-431.

4. Melatonin pharmacokinetics following two different oral surge-sustained release doses in older adults. Gooneratne N et al., J Pineal Res 2012; 52: 437-445.

3.3.5. GLUTATHIONE (Tationil®)

In humans, recent investigations have proven the therapeutic potential of *glutathione*, in particular since it has a low toxicity in humans (1) and it is cost-effective. Chawla et al. has described a lower concentration of GSH in the plasma of cirrhotic patients (2). Thus, any intervention increasing hepatic or plasma *glutathione* levels should convey protection against reperfusion injury (3).

Overall, *glutathione* and precursor (acetylcysteine) appears to have a low toxicity in humans. Currently, there are no known side effects of *glutathione* (1). None adverse reactions have been reported, neither in clinical studies nor in case reports.

It must be noted that the best dosage of *glutathione* has not been clearly established for any use; most reported dose schedules have been empirically chosen based on animal and clinical data. Applied IV concentrations vary from 2.0 to 4.8 gram in total for an adult. For example, *glutathione* has been reported in randomized studies to reduce the incidence of neurotoxicity induced by chemotherapy at a dose of 1.5 g/m^2 (4, 5, 6). IV *glutathione* is also used to slow down the progression of Parkinson's disease. Most patients are given 1400 mg along with saline for ten minutes, three times a week. It has been shown to have a critical function as elimination of toxic compounds and by restoring the hepatic glutathione stores in case of acetaminophen intoxication. In that case, an approved 20 hour IV protocol for acetylcysteine –precursor of glutathione- treatment has been used in the UK since 1970s. This treatment protocol provides a total of 300 mg/Kg over 20 hours with an initial loading dose of 150 mg/kg IV over 15 to 60 minutes. This treatment period is often extended when patients have large ingestions of acetaminophen or elevated serum transaminase (7).

In this study, the IV dose of *glutathione* has been determined according the experimental and clinical data that have proven profound effect without toxicological events. According the short half-life, 3 gr will be administrated 2-4 minutes just before the reperfusion.

<u>*References*</u>

1. High-dose intravenous glutathione in man. Pharmacokinetics and effects on cysteine in plasma and urine. Aebi et al., Eur J Invest 1991; 21: 103-110.

2. Plasma cysteine, cystine, and glutathione in cirrhosis. Chawla et al., Gastroenterology 1984; 87: 770.

3. Plasma membrane and mitochondrial transport of hepatic reduced glutathione. Fernández-Checa JCet al. Semin Liver Dis. 1996; 16:147–158.

4. Glutathione protects the rat liver against reperfusion injury after hypothermic preservation. Bilzer M et al., Gastroenterology. 1999; 117:200–210.

5. Beneficial effects of extracellular glutathione against endotoxin-induced liver injury during ischemia and reperfusion. Liu P et al., Circ Shock. 1994;43:64–70.

CONFIDENTIAL

6. Neuroprotective effect of reduced glutathione on cisplatin-based chemotherapy in advanced gastric cancer: a randomized double-blind placebo-controlled trial. Cascinu et al., J Clin Oncol 1995; 13: 26-32.
7. Treatment of paracetamol (acetaminophen) poisoning with N-acetylcysteine. Prescott LF, Park J, Ballantyne A, Adriaenssens P, Proudfoot AT. Lancet. 1977;2(8035):432

3.3.6. INFLIXIMAB (Remicade®)

Infliximab (Remicade®) is well established in the treatment of a few inflammatory diseases: rheumatoid arthritis (I.V. 3 mg/kg at 0, 2, and 6 weeks, followed by 3 mg/kg every 8 weeks thereafter; doses have ranged from 3-10 mg/kg repeated at 4- to 8-week intervals), psoriatric arthritis (I.V.: 5 mg/kg at 0,2, and 6 weeks, followed by 5 mg/kg every 8 weeks thereafter), ankylosing spondylitis (I.V.: 5 mg/kg at 0, 2, and 6 weeks, followed by 5 mg/kg every 6 weeks thereafter), inflammatory bowel diseases (I.V.: 5 mg/kg at 0, 2, and 6 weeks, followed by 5 mg/kg every 8 weeks thereafter; dose may be increased to 10 mg/kg in patients who respond but then lose their response), and several studies have demonstrated a high efficacy with rapid overall patient improvement, maintenance treatment, and decrease in rates of hospitalizations and surgical interventions (1). Infliximab (Remicade®) has been also studied extensively in the treatment of patients with sepsis (2, 3). These studies have revealed that anti-cytokine administration usually as a single dose is safe but not particularly efficacious in the management of sepsis.
Furthermore, clinical experience with TNF-α inhibitors after solid organ transplantation has revealed good results in terms of safety, reduction in toxicity and allograft survival (4). In some case reports, infliximab (Remicade®) has been shown to be a therapeutic option for refractory acute rejection after intestinal transplantation (5). Currently in Leuven, infliximab (Remicade®) at the dose of 3 mg/Kg is infused in patient during intestine transplantation at the same timing that foreseen in this protocol.
In this study, the IV dose of Infliximab has been determined according to the experimental and clinical data that have proven profound effect without toxicological events (2, 3, 4). Thus, 3 mg/kg of Infliximab will be administrated during 3 hours from the beginning of the anhepatic phase after administration of Antithrombin III.

CONFIDENTIAL

<u>References</u>

1.Long-term outcome of treatment with infliximab in 614 patients with Crohn's disease: results from a single-center cohort. Schnitzler et al., Gut 2009; 58: 492-500.
2.Hepatotoxicity of immunomodulating agents and the transplant situation. Kaplowitz et al., Drug-induced liver disease 2007; 662-681.
3.Review article: the role of tumor necrosis factor in renal ischemia-reperfusion injury. Donnahoo et al., The Journal of Urology 1999, 162: 196-203.
4.New monoclonal antibodies in renal transplantation. Vincenti et al., Minerva Urol Nefrol 2003; 55: 57-66.
5.Intestinal transplantation: evolution in immunosuppression protocols. Pirenne et al., Curr Opin Organ Transplant 2009; 14: 250-255.

3.3.7. α-TOCOPHEROL (vitamin E suspension 100 mg/ml®)

This major antioxidant vitamin has excellent safety data in doses that far exceed the doses required to reach adequate antioxidant protection in vivo. Regarding controlled, double-blinded studies of vitamin E toxicity in humans, several reports confirm that vitamin E has very low toxicity and no consistent adverse events have been reported (1, 2). Ferslew et al. (3) described that α-tocopherol first appeared in the plasma in 2-4 hours after oral administration and peaked at 5-14 hours when administering 800 mg α-Tocopherol to healthy volunteers. This study was not associated with any adverse events.

Lassnig et al. (2) have studied multiple parenteral vitamin E infusion in patients undergoing elective cardiac surgery. A total dose of 1080 mg of vitamin E was administered at four points in time: two infusions before the start of surgery and one infusion each on days 1 and 2 after surgery. Based on pharmacokinetic data the dose was considered sufficient to maintain Vitamin E concentrations during surgery. Since no protective effect of Vitamin E supplementation was observed, Lassnigg et al. suggested that the supply of Vitamin E was inadequate and/or that not enough Vitamin E was incorporated into the target cells. Based on these findings and investigations, Bartels et al. (4) decided to administer 1620 mg the day before the liver surgery and compared to a placebo group the extent of IRI. After three infusions of vitamin E, there was a significant fivefold increase in the plasma concentration; half of this increase remained about 12 h later, prior to surgery. After reperfusion, the vitamin E concentration was significantly decreased in both groups. It is likely that, due to severe oxidative stress, the need for and consumption of, α-Tocopherol was great. Six days after surgery the treated group still had plasma vitamin E concentrations comparable to the baseline value, whereas the placebo group still had significantly reduced plasma vitamin E concentrations. They have shown that the preoperative parenteral administration of 1620 mg of α-Tocopherol was safe, not only leads to a marked increase of plasma concentration but also prevents vitamin E depletion after IRI.

Currently, no solutions containing Vitamin E alone are available for human use. Therefore, 5 mL of Vitamin E suspension® (500 mg) will be administrated orally at the ward before transport to the operative theatre. This time course is adequate according the pharmacokinetic data of Vitamin E after oral administration.

References

1. Safety of antioxidant vitamins and B-carotene. Diplock et al., Am J Clin Nutr 1995; 62: 1510-1516.

2. Influence of intravenous vitamin E supplementation in cardiac surgery on oxidative stress: a double-blinded, randomized, controlled study. Br J Anaesth 2003; 90: 148-154.

3. Pharmacokinetics and bioavailability of the RRR and all racemic stereoisomers of alpha-tocopherol in humans after single oral administration. Ferslew et al., J Clin Pharmacol 1993; 33: 84-88.

4. Pilot study on the effect of parenteral vitamin E on ischemia and reperfusion induced liver injury: a double-blind, randomized, placebo-controlled trial. Bartels et al., Clin Nutrit 2004; 23: 1360-1370.

3.3.8. APOTRANSFERRIN

CONFIDENTIAL

Apotransferrin is purified from human plasma. Sahlstedt et al. (1) have demonstrated that a single dose of Apotransferrin (100 mg/kg) was effective and safe on the serum iron binding capacity. Parkkinen et al. have described in a dose-finding study the highest dose level for the prevention of appearance of NTBI in patients with myeloablation. A total dose of 1040 mg/kg (115 mg/Kg/day for 9 days) was given to the patients without any related serious adverse event (2).
In this study, the IV dose of apotransferrin has been determined according to the experimental and clinical data that have proven profound effect without toxicological events (1, 2). Thus, 170 mg/Kg of Apotransferrin® will be infused during 3 hours from the beginning of the anhepatic phase.

<u>*References*</u>

1. Effective binding of free iron by a single intravenous dose of human apotransferrin in haematological stem cell transplant patient. Sahlstedt et al., Br J Haemato 2002; 119: 547-553.
2. Effect of repeated apotransferrin administrations on serum iron parameters in patients undergoing myeloablative conditioning and allogenic stem cell transplantation. Parkkinen et al., Br J Haemato 2006; 135: 228-234.

3.3.9. EPOPROSTENOL (Flolan®)

In clinic, recent investigations have proven the therapeutic potential of epoprostenol (Flolan®) in transplantation. Epoprostenol (Flolan®) was originally chosen for lung preservation because it allowed a more even distribution of cold perfusion during the cooling of organs. Its use has then been extended to other organs. Klein et al. has demonstrated a significant impact of the epoprostenol (Flolan®) in preconditioning the graft prior to the LTx (1). Indeed, an injection of 500 µg PGI2 in the donor has shown an improvement of the IRI due to the prevention of thrombosis and vasospasm after cold storage. Moreover, Muhlbacher et al. (2) has demonstrated that pretreatment with epoprostenol (Flolan®) (200 µg) to organ donors has also an impact on the kidney. He observed a reduction of the number of post-transplant dialyses and hospital care days in patients undergoing renal transplantation. Finally, Pirenne et al. demonstrated that donor treatment with epoprostenol (Flolan®) for better flushing and preserving peribiliary vascular plexus and biliary mucosa offers protection from biliary strictures (3).
To avoid any side effects in the recipient, an ex-situ administration of epoprostenol (Flolan®) has been chosen. Epoprostenol (Flolan®) will be administrated directly through the vena porta during the bench table (ex-situ). By doing so, no systemic absorption at the reperfusion is expected in the recipient. After reconstitution, 50 cc of epoprostenol (Flolan®) will be added to a liter of preservation solution. 500 cc will be flushed through the vena porta. The IV dose of epoprostenol (Flolan®) has been determined according our clinical data. In case of DCD procedure with liver and lung procurement, we routinely use a preconditioning with 65 cc of Flolan®. 38 cc of reconstituted Flolan® are diluted in the lung cold perfusion whereas 7 cc are directly administrated through the pulmonary artery before the cooling of the lungs. Moreover 20 cc are diluted in the abdominal cold pre-flush solution.

CONFIDENTIAL

References

1. Preconditioning of donor livers with prostaglandin I2 before retrieval decreases hepatocellular ischemia-reperfusion injury. Klein et al., Transplantation 1999; 67: 1128-1132.

2. Improved renal graft function after prostacyclin pretreatment. Muhlbacher et al., Transplant Proc 1987; 19: 4162-4163.

3. Biliary strictures after liver transplantation: risk factors and prevention by donor treatment with epoprostenol. Pirenne et al., Transplant Proc 2009; 41: 3399-3402.

CONFIDENTIAL

APPENDIX 4: SCHEDULE OF EVENTS

	Before TX	Baseline day of TX	immediately before hepatectomy	immediately before reperfusion	30-60-120 minutes after reperfusion	6-12-24-48-72 hours after reperfusion	Day 1 to 7	Day 14	Month 3	Month 12
Informed consent	**X**									
Recipient details		**X**								
Medical history		**X**								
Lab MELD score		**X**								
Donor details		**X**								
Surgical procurement details		**X**								
Plasma sample (AST)		**X**	**X**	**X**	**X**	**X**	**X**	**X**	**X**	**X**
Liver biopsy				**X (before implantation)**	**X (1 hr after reperfusion)**		**X (1 week after reperfusion)**			
EDTA/serum samples			**X**	**X**	**X**	**X**	**X**	**X**	**X**	**X**
Urine samples			**X**	**X**	**X**	**X**	**X**	**X**	**X**	**X**
Data collection			**X**	**X**	**X**	**X**	**X**	**X**	**X**	**X**
Tacrolimus trough levels							**X**	**X**	**X**	**X**
Adverse events monitoring (on going)		**X**	**X**	**X**	**X**	**X**	**X**	**X**	**X**	**X**
Surgical complications monitoring (on going)		**X**	**X**	**X**	**X**	**X**	**X**	**X**	**X**	**X**

CONFIDENTIAL

APPENDIX 5: ETHICAL PRINCIPLES FOR MEDICAL RESEARCH INVOLVING HUMAN SUBJECTS

Adopted by the 18th World Medical Assembly
Helsinki, Finland, June 1964
and amended by the
29th World Medical Assembly, Tokyo, Japan, October 1975
35th World Medical Assembly, Venice, Italy, October 1983
41st World Medical Assembly, Hong Kong, September 1989
48th General Assembly, Somerset West, Republic of South Africa, October 1996
and the
52nd WMA General Assembly, Edinburgh, Scotland, October 2000

1. Introduction

1. The World Medical Association has developed the Declaration of Helsinki as a statement of ethical principles to provide guidance to physicians and other participants in medical research involving human subjects. Medical research involving human subjects includes research on identifiable human material or identifiable data.

2. It is the duty of the physician to promote and safeguard the health of the people. The physician's knowledge and conscience are dedicated to the fulfillment of this duty.

3. The Declaration of Geneva of the World Medical Association binds the physician with the words, "The health of my patient will be my first consideration," and the International Code of Medical Ethics declares that, "A physician shall act only in the patient's interest when providing medical care which might have the effect of weakening the physical and mental condition of the patient."

4. Medical progress is based on research which ultimately must rest in part on experimentation involving human subjects.

5. In medical research on human subjects, considerations related to the well-being of the human subject should take precedence over the interests of science and society.

6. The primary purpose of medical research involving human subjects is to improve prophylactic, diagnostic, and therapeutic procedures and the understanding of the etiology and pathogenesis of disease. Even the best proven prophylactic, diagnostic and therapeutic methods must continuously be challenged through research for their effectiveness, efficiency, accessibility, and quality.

7. In current medical practice and in medical research, most prophylactic, diagnostic and therapeutic procedures involve risks and burdens.

8. Medical research is subject to ethical standards that promote respect for all human beings and protect their health and rights. Some research populations are vulnerable and need special protection. The particular needs of the economically and medically

CONFIDENTIAL

disadvantaged must be recognized. Special attention is also required for those who cannot give or refuse consent for themselves, for those who may be subject to giving consent under duress, for those who will not benefit personally from the research and for those for whom the research is combined with care.

9. Research Investigators should be aware of the ethical, legal, and regulatory requirements for research on human subjects in their own countries as well as applicable international requirements. No national ethical, legal or regulatory requirement should be allowed to reduce or eliminate any of the protections for human subjects set forth in this Declaration.

2. Basic principles for all medical research

10. It is the duty of the physician in medical research to protect the life, health, privacy and dignity of the human subject.

11. Medical research involving human subjects must conform to generally accepted scientific principles, be based on a thorough knowledge of the scientific literature, other relevant sources of information, and on adequate laboratory and where appropriate, animal experimentation.

12. Appropriate caution must be exercised in the conduct of research which may affect the environment and the welfare of animals used for research must be respected.

13. The design and performance of each experimental procedure involving human subjects should be clearly formulated in an experimental protocol. This protocol should be submitted for consideration, comment, guidance, and where appropriate, approval to a specially appointed ethical review committee, which must be independent of the investigator, the sponsor or any other kind of undue influence. This independent committee should be in conformity with the laws and regulations of the country in which the research experiment is performed. The committee has the right to monitor ongoing trials. The researcher has the obligation to provide monitoring information to the committee, especially any serious adverse events. The researcher should also submit to the committee, for review, information regarding funding, sponsors, institutional affiliations, other potential conflicts of interest and incentives for subjects.

14. The research protocol should always contain a statement of the ethical considerations involved and should indicate that there is compliance with the principles enunciated in this Declaration.

15. Medical research involving human subjects should be conducted only by scientifically qualified persons and under the supervision of a clinically competent medical person. The responsibility for the human subject must always rest with a medically qualified person and never rest on the subject of the research, even though the subject has given consent.

16. Every medical research project involving human subjects should be preceded by careful assessment of predictable risks and burdens in comparison with foreseeable

CONFIDENTIAL

benefits to the subject or to others. This does not preclude the participation of healthy volunteers in medical research. The design of all studies should be publicly available.

17. Physicians should abstain from engaging in research projects involving human subjects unless they are confident that the risks involved have been adequately assessed and can be satisfactorily managed. Physicians should cease any investigation if the risks are found to outweigh the potential benefits or if there is conclusive proof of positive and beneficial results.

18. Medical research involving human subjects should only be conducted if the importance of the objective outweighs the inherent risks and burdens to the subject. This is especially important when the human subjects are healthy volunteers.

19. Medical research is only justified if there is a reasonable likelihood that the populations in which the research is carried out stand to benefit from the results of the research.

20. The subjects must be volunteers and informed participants in the research project.

21. The right of research subjects to safeguard their integrity must always be respected. Every precaution should be taken to respect the privacy of the subject, the confidentiality of the patient's information and to minimize the impact of the study on the subject's physical and mental integrity and on the personality of the subject.

22. In any research on human beings, each potential subject must be adequately informed of the aims, methods, sources of funding, any possible conflicts of interest, institutional affiliations of the researcher, the anticipated benefits and potential risks of the study, and the discomfort it may entail. The subject should be informed of the right to abstain from participation in the study or to withdraw consent to participate at any time without reprisal.
After ensuring that the subject has understood the information, the physician should then obtain the subject's freely-given informed consent, preferably in writing. If the consent cannot be obtained in writing, the non-written consent must be formally documented and witnessed.

23. When obtaining informed consent for the research project the physician should be particularly cautious if the subject is in a dependent relationship with the physician or may consent under duress. In that case the informed consent should be obtained by a well-informed physician who is not engaged in the investigation and who is completely independent of this relationship.

24. For a research subject who is legally incompetent, physically or mentally incapable of giving consent or is a legally incompetent minor, the investigator must obtain informed consent from the legally authorized representative in accordance with applicable law. These groups should not be included in research unless the research is necessary to promote the health of the population represented and this research cannot instead be performed on legally competent persons.

CONFIDENTIAL

25. When a subject deemed legally incompetent, such as a minor child, is able to give assent to decisions about participation in research, the investigator must obtain that assent in addition to the consent of the legally authorized representative.

26. Research on individuals from whom it is not possible to obtain consent, including proxy or advance consent, should be done only if the physical/mental condition that prevents obtaining informed consent is a necessary characteristic of the research population. The specific reasons for involving research subjects with a condition that renders them unable to give informed consent should be stated in the experimental protocol for consideration and approval of the review committee. The protocol should state that consent to remain in the research should be obtained as soon as possible from the individual or a legally authorized surrogate.

27. Both authors and publishers have ethical obligations. In publication of the results of research, the investigators are obliged to preserve the accuracy of the results. Negative as well as positive results should be published or otherwise publicly available. Sources of funding, institutional affiliations and any possible conflicts of interest should be declared in the publication. Reports of experimentation not in accordance with the principles laid down in this Declaration should not be accepted for publication.

3. Additional principles for medical research combined with medical

28. The physician may combine medical research with medical care, only to the extent that the research is justified by its potential prophylactic, diagnostic or therapeutic value. When medical research is combined with medical care, additional standards apply to protect the patients who are research subjects.

29. The benefits, risks, burdens, and effectiveness of a new method should be tested against those of the best current prophylactic, diagnostic, and therapeutic methods. This does not exclude the use of placebo, or no treatment, in studies where no proven prophylactic, diagnostic or therapeutic method exists.

30. At the conclusion of the study, every patient entered into the study should be assured of access to the best proven prophylactic, diagnostic, and therapeutic methods identified by the study.

31. The physician should fully inform the patient which aspects of the care are related to the research. The refusal of a patient to participate in a study must never interfere with the patient-physician relationship.

32. In the treatment of a patient, where proven prophylactic, diagnostic, and therapeutic methods do not exist or have been ineffective, the physician, with informed consent from the patient, must be free to use unproven or new prophylactic, diagnostic and therapeutic measures, if in the physician’s judgment it offers hope of saving life, reestablishing health or alleviating suffering. Where possible, these measures should be made the object of research, designed to evaluate their safety and efficacy. In all cases, new information should be recorded and, where appropriate, published. The other relevant guidelines of this Declaration should be followed.

CONFIDENTIAL

APPENDIX 6: INFORMED CONSENT

Toestemmingsformulier

Toediening van geneesmiddelen om leverbeschadiging tijdens transplantatie te verminderen

Protocol Nr:

Sponsor: UZ Leuven, Herestraat 49, 3000 Leuven

Dit formulier is enkel bestemd voor de patiënt of zijn/haar wettelijke vertegenwoordiger.

Ik, ……………………………………………………………………………………………, bevestig dat ik geïnformeerd ben over de klinische studie en dat ik een kopie van de 'Patiëntenbrochure' en het 'Toestemmingsformulier' ontvangen heb. Ik heb deze informatie gelezen en begrepen. Mijn dokter heeft me voldoende informatie gegeven betreffende de voorwaarden, de duur van de studie, het effect en de bijwerkingen van de behandeling. Daarbij heb ik voldoende tijd gehad om deze informatie te overwegen en vragen te stellen waarop ik voldoende en begrijpbare antwoorden heb gekregen.

Ik aanvaard eveneens dat weefsel-, bloed- en urinestalen, afgenomen gedurende het volledige verloop van de studie (vóór, tijdens en na de transplantatie), gebruikt worden voor wetenschappelijke doeleinden.

Ik heb begrepen dat ik op ieder moment kan stoppen met mijn deelname aan deze studie, nadat ik mijn dokter hierover heb geïnformeerd en dat deze beslissing geen enkel nadeel zal hebben voor mij.

Ik geef toestemming aan de verantwoordelijke onderzoekers van de studie en de regulerende instanties om toegang te hebben tot mijn medische gegevens. Mijn medische gegevens zullen strikt persoonlijk behandeld worden. Ik ben mij bewust dat mijn medische gegevens zullen worden verzameld, verwerkt en gebruikt binnen de context van deze studie.

Ik ga akkoord bij het gebruik van mijn medische data door de onderzoeker voor andere onderzoeksdoelstellingen.

Ik geef vrijwillig mijn toestemming om deel te nemen aan deze studie en mee te werken in alle vereiste onderzoeken. Ik ben bereid om informatie te geven omtrent mijn medische voorgeschiedenis, gebruik van medicijnen en deelname aan andere studies indien van toepassing.

Ik ga akkoord dat alle zorgverleners, die betrokken zijn in deze studie, ingelicht zullen zijn over mijn deelname aan deze studie.

Ik geef toestemming voor deelname aan het onderzoek.

Achternaam en voornaam:

Geboortedatum:

Handtekening:

CONFIDENTIAL

Datum:

Ondergetekende verklaart dat de hierboven genoemde persoon zowel mondeling als schriftelijk over het bovenvermelde onderzoek is geïnformeerd.

Naam:

Functie:

Handtekening:

Datum:

CONFIDENTIAL

Consent form

Combined drug approach to Prevent Liver damage during Transplantation

Protocol Nr:

Sponsor: UZ Leuven, Herestraat 49, 3000 Leuven

Part which is destined only to the patient or his/her legal representative

I, …………………………………………………………………………………………………… confirm that I have been informed about the clinical study and that I have received a copy of the patient information sheet and the consent form. I have read and understood the information. My doctor has given me sufficient information concerning the conditions, the length of the study, the effect and the side effects of this treatment. In addition, I have received sufficient time to consider the information and to ask questions to which I have received satisfying answers.
I accept that blood, urine and tissue samples that will be taken during the whole study (before, during and after the transplantation) will be used for scientific purposes.
I have understood that I can stop my participation in this study at any time after having informed my doctor about this and that this decision will not cause any disadvantage.
I authorize the investigators of the study and the regulating authorities to have access to my medical records. My medical data will be treated strictly confidential. I am aware of the purpose for which these data are collected, processed and used in the context of the study. I agree with the collection, the processing and the use of these medical data, as described in the patient information sheet.
I agree with the use by the investigator of these coded medical data for other research purposes.
I consent voluntarily to participate in this study and to cooperate in all the examinations requested. I am willing to give information concerning my medical history, use of medication(s) and participation in other studies if any.
I agree that all the healthcare professionals involved in my treatment are informed about my participation in this study.

I give permission for participation in the study.

Last en first name :

Date of birth :

Signature :

Date :

Undersigned declares that the person above is informed oral and by letter about the study.

Name :

CONFIDENTIAL

Function :

Signature :

Date :

CONFIDENTIAL

Consentement éclairé

Réduction des lésions du foie en transplantation après administration séquentielle de médicaments (CAPITL)

Protocole Nr:

Promoteur: UZ Leuven, Herestraat 49, 3000 Leuven

Partie réservée au patient ou à son représentant légal

Je soussigné, ……………………………………………………………………………………………………

certifie avoir été informé au sujet de cette étude et avoir reçu une copie de la brochure informative et du présent consentement éclairé. J'ai lu et compris tous les tenant et aboutissant. Mon médecin m'a donné suffisamment d'informations requises pour une bonne compréhension des conditions de l'étude, de l'action et des potentiels effets secondaires des médicaments. En outre, j'ai bénéficié de suffisamment de temps nécessaire à la compréhension de ces informations. J'assure que les réponses concernant mes questions étaient adaptées et complètes pour ma compréhension.
J'accepte par le présent consentement que des échantillons de sang, d'urine et de tissue soient prélevés à des fins purement scientifiques avant, durant et après la transplantation de foie.
J'ai compris que je peux à tout moment refuser de continuer de participer à l'étude après en avoir averti le médecin et ce sans le moindre inconvénient ou disparité pour le suivi de la greffe.
J'autorise les investigateurs et les autorités compétentes à pouvoir accéder, de façon strictement confidentielle à mon dossier médicales. J'approuve l'analyse de ces informations personnelles dans le contexte de l'étude comme décrit dans la brochure informative.
Je déclare participer volontairement à cette étude et à coopérer à la totalité des examens requis. J'accepte de donner des informations concernant mes antécédents médicaux. Je déclarerais ma participation à d'autre potentielle étude.
Je permets au personnel médical et paramédical impliqué dans mon traitement d'être au courant de ma participation à l'étude.

CONFIDENTIAL

Je suis d'accord de participer à l'étude.

Nom et prénom :

Date de naissance :

Signature :

Date :

La personne sous-mentionnée declare que le patient concerné a été clairement informé oralement et par écrit au sujet de l'étude.

Nom :

Fonction :

Signature :

Date :

CONFIDENTIAL

Einverständniserklärung

Medikamentöse Strategien zur Reduktion des während einer Lebertransplantation auftretenden Leberschadens

Protokoll Nr.:

Sponsor: University Hospitals Leuven, Herestraat 49, 3000 Leuven

Dieses Formular ist allein für den Patienten oder seinen gesetzlichen Vertreter bestimmt.

Ich,..., bestätige, dass ich über die klinische Studie informiert wurde und dass ich eine Kopie der „Informationsbroschüre" und der "Einverständniserklärung" erhalten habe. Ich habe diese Informationen gelesen und verstanden. Mein Arzt hat mich umfassend informiert über die Bedingungen zur Teilnahme an der Studie, die Dauer der Studie, sowie die Wirkungen und Nebenwirkungen der Behandlung. Ich hatte ausreichend Zeit, um diese Informationen zu überdenken und Fragen zu stellen, auf die ich ausreichende und verständliche Antworten erhalten habe.

Ich akzeptiere ebenfalls, dass Gewebe-, Blut- und Urinproben, die während des gesamten Verlaufs der Studie (vor, während und nach der Transplantation) abgenommen werden, für wissenschaftliche Zwecke verwendet werden.

Ich habe verstanden, dass ich jederzeit meine Teilnahme an dieser Studie widerrufen kann, indem ich meinen Arzt darüber in Kenntnis setze, und dass diese Entscheidung keinen einzigen Nachteil für mich haben wird.

Ich ermächtige die zuständigen Prüfarzte und die Aufsichtsbehörden zum Zugriff auf meine Krankenakten. Die mich betreffenden medizinischen Informationen werden streng vertraulich behandelt werden. Ich bin mir bewusst, dass für die Untersuchung meine medizinischen Daten gesammelt, verarbeitet und ausgewertet werden.

Ich stimme zu, dass der Prüfarzt meine medizinischen Daten auch für andere Forschungsvorhaben nutzen kann.

Ich gebe freiwillig meine Zustimmung, an dieser Studie und an allen erforderlichen Untersuchungen teilzunehmen. Ich bin bereit, Informationen zu geben über meine medizinische Vorgeschichte, die Einnahme von Medikamenten und die eventuelle Teilnahme an anderen Studien.

Ich bin damit einverstanden, dass alle Gesundheitsdienstleister, die an dieser Studie beteiligt sind, über meine Teilnahme an dieser Studie informiert werden.

Ich gebe die Einwilligung zur Teilnahme an dieser klinischen Studie.

Name und Vorname:

Geburtsdatum:

Unterschrift:

Datum:

CONFIDENTIAL

Ich erkläre, dass die oben genannte Person mündlich und schriftlich über die oben genannten Studie informiert wurde.

Name:

Funktion:

Unterschrift:

Datum:

CONFIDENTIAL

APPENDIX 7: PATIENT INFORMATION BROCHURE: Part A. Safety study

Informatiebrochure

Toediening geneesmiddelen om leverbeschadiging tijdens transplantatie te verminderen

Patiënten nr:

U wordt gevraagd om deel te nemen aan een klinische studie waarbij verschillende medicijnen tijdens uw levertransplantatie toegediend zullen worden met als doel de beschadiging van de lever tijdens het transplantatie proces (zogenoemde ischemie-reperfusie schade) te verminderen. Het is belangrijk om dit document te lezen alvorens u beslist om aan deze studie deel te nemen. De doelstellingen, onderzoeken, voor- en nadelen, risico's en ongemakken die aan deze studie verbonden zijn, worden in de informatiebrochure en het toestemmingsformulier beschreven. Er kan niets worden beloofd noch worden gegarandeerd in verband met de resultaten van de studie. U hebt het recht om, om het even wanneer, vragen te stellen met betrekking tot de mogelijke en/of bestaande risico's in deze studie.

Doelstelling en beschrijving van de studie

Tijdens het chirurgisch verwijderen, bewaren en transplanteren van een lever kan dit orgaan beschadigd worden. Deze beschadiging is deels het gevolg van een onvermijdbaar zuurstoftekort tijdens de bewaring van de lever na het chirurgisch verwijderen tot de transplantatie(ook ischemie genoemd).
Deze ischemische schade kan echter na de levertransplantatie versterkt worden wanneer de bloeddoorstroming in de ontvanger hersteld wordt (dit noemt men ischemie-reperfusie schade). De opgelopen orgaanschade kan minimaal maar kan ook zeer ernstig tot levensbedreigend zijn. Een vermindering van de ischemie-reperfusie schade is dan ook van groot belang omdat het de resultaten van levertransplantatie kan verbeteren.
Het proces van ischemie-reperfusie schade bestaat uit een complexe cascade van elkaar versterkende processen.
Het doel van deze studie is dan ook ischemie-reperfusie schade na levertransplantatie te verminderen tot minimaliseren.
Om al deze schadelijke processen te blokkeren, voorzien we om verschillende geneesmiddelen toe te dienen zodat de kans op succes zo groot mogelijk is. Deze combinatie aan geneesmiddelen zal voor het eerst bij de mens worden gebruikt en wordt tijdens de levertransplantatie toegediend. Ondertussen zullen uw bloeddruk, hartritme en temperatuur voortdurend gemeten worden.
Het doel van deze studie is de veiligheid van deze geneesmiddelen aan te tonen (een zogenaamde safety studie). Wanneer u besluit om aan deze studie deel te nemen, zal u naast de standaardbehandeling aan de bijkomende toegediende geneesmiddelen zoals voorzien in deze studie worden onderworpen.

CONFIDENTIAL

De geneesmiddelen die toegediend zullen worden, zijn:

- *Cetor®/Cinryze®*: C1-esterase inhibitor. C1-esterase inhibitor speelt een belangrijke rol in de onstekingsreactie en bloedstolling.
- *Atenativ®*: anti-stolling, anti-ontsteking
- *Vitamin E oplossing®*: Vitamine E biedt bescherming tegen de schadelijke effecten van zuurstof
- *Tationil® 600 en Circadin®*: biedt bescherming tegen de schadelijke effecten van zuurstof
- *Apotransferrine*: houdt ijzer vast en biedt ook bescherming tegen de schadelijke effecten van zuurstof
- *Neorecormon®*: biedt bescherming tegen celdood en de schadelijke effecten van zuurstof, zorgt voor een vermindering van ontstekingssignalen
- *Remicade®*: vermindering van ontstekingssignalen
- *Flolan®*: anti-ontsteking, blokkeren van de activatie van bloedplaatjes en verwijding van de bloedvaten

Er zullen 10 patiënten aan deze studie deelnemen.

Tijdsduur studie

De safety studie zal 1 week duren.

Onderzoeken

- Zoals iedereen die een levertransplantatie ondergaat, zullen er dagelijks en routinematig bloed- en urinestalen worden afgenomen de eerste 14 dagen na levertransplantatie. Ook standaard klinische onderzoeken zullen plaatsvinden en dit 3, 6, 12 maanden na de transplantatie op de raadpleging.
- Bovendien worden ook 2 leverbiopsies genomen:
    - 1 uur voor de implantatie
    - 1 uur na de reperfusie

Vrijwillige deelname

Uw deelname aan deze studie is volledig vrijwillig. U hebt het recht om deelname te weigeren. Wat u ook beslist, u zal behandeld en opgevolgd worden zonder enig nadelig gevolg voor uw medische zorgen en voor uw relatie met uw behandelende arts (dokter-patiënt relatie).
Wanneer u beslist om aan deze studie deel te nemen, dan zal er aan u gevraagd worden het toestemmingsformulier te ondertekenen.
Nadat u het toestemmingsformulier hebt ondertekend is het uw recht om uw deelname aan de studie te stoppen om het even wanneer, U moet geen reden geven waarom u uw toestemming voor deelname aan de studie intrekt.

CONFIDENTIAL

De studiedokter kan op elk moment uw deelname aan de studie stopzetten en dit zonder uw toestemming te vragen.

Risico's en ongemakken

Er zal maar één dosis (twee van Neorecormon®) van ieder geneesmiddel in deze studie worden toegediend. De veiligheid van toediening van deze componenten wordt specifiek onderzocht in deze safety studie. Onverwachte interacties/potentiële risico's eigen aan de combinatie kunnen niet helemaal uitgesloten worden. Het kan ook zijn dat u bijwerkingen tijdens de behandeling met deze medicijnen ondervindt, namelijk:

- *Cetor®/Cinryze®:* Allergische of anafylactische reacties (bijvoorbeeld: versneld hartritme, hoge of lage bloeddruk, roodheid in de hals en in het gezicht, netelroos, kortademigheid)
- *Atenativ*®: Allergische reacties of overgevoeligheid, toename in lichaamstemperatuur en verwijdering van de bloedvaten
- *Vitamine E oplossing*®: geen gekende bijwerkingen
- *Tationil® 600*: geen gekende bijwerkingen
- *Circadin*®: geen gekende bijwerkingen
- *Apotransferrine*: geen gekende bijwerkingen
- *Neorecormon*®: vorming van een bloedklonter in een bloedvat, epilepsie en hoge bloeddruk
- *Remicade*: huiduitslag, afwijkingen in de samenstelling van bloed, overgevoeligheid en levensbedreigende allergische reacties
- *Flolan*®: hoofdpijn, misselijkheid, lage bloeddruk, traag hartritme, versneld hartritme en kortademigheid

Voordelen

We kunnen het therapeutisch effect van de geneesmiddelen niet verzekeren zelfs al weten we dat ieder geneesmiddel op zich de intensiteit van ischemie-reperfusie schade vermindert.

Verzekering

Conform de Belgische wet inzake experimenten op de menselijke persoon van 7 mei 2004 is de sponsor van het onderzoek, zelfs foutloos, aansprakelijk voor alle schade die de deelnemer en/of zijn rechthebbenden oplopen en die rechtstreeks dan wel onrechtstreeks verband houdt met de proef. De sponsor heeft een verzekering afgesloten die deze aansprakelijkheid dekt. Indien u schade zou oplopen ten gevolge van uw deelname aan deze studie, zal die schade bijgevolg worden vergoed conform de Belgische wet inzake experimenten op de menselijke persoon van 7 mei 2004.

Compensaties

CONFIDENTIAL

Deze studie brengt voor U geen extra kosten met zich mee. U hoeft de geneesmiddelen ook niet te betalen. Bovendien worden alle onderzoeken op stalen (bloed-, urine- en leverbiopsies) die niet beschouwd worden als courant en routinematig, betaald door de onderzoeker.

Bescherming van uw privéleven

Uw identiteit en deelname aan deze studie zullen strikt vertrouwelijk behandeld worden. U zal niet geïdentificeerd kunnen worden bij naam of op enige andere manier in documenten, resultaten of publicaties betreffende de studie.
Uw medische gegevens zullen onderzocht worden door vertegenwoordigers van de onderzoeker en door regulerende instanties die de studie ook controleren. Dit gebeurt volgende de GCP (= Good Clinical Practice) richtlijnen. Hierbij wordt Uw identiteit geheim gehouden door een uniek patiëntennummer te gebruiken om Uw persoonlijke informatie aan te duiden.
Uw persoonlijke informatie zal doorgegeven worden aan de regulerende instanties, de ethische commissie en andere dokters die samenwerken met de onderzoeker.
Uw persoonlijke informatie zal elektronisch of manueel verwerkt en geanalyseerd worden om de resultaten van deze studie te bepalen. U hebt het recht aan de dokter van de studie te vragen naar uw data die verzameld zijn en de doelstelling van deze verzameling. U hebt ook het recht om de studiedokter te verzoeken om u toegang te geven tot uw persoonlijke informatie en die te verbeteren indien nodig.
Alle reglementering betreffende de bescherming van de persoonlijke data is terug te vinden in de Wet van 8 december 1992 (België), betreffende de bescherming van het privéleven.

Nieuwe informatie

Soms is het mogelijk dat nieuwe informatie omtrent behandeling of medicinale producten in de context van het klinisch project verschijnt. Indien dit het geval is, zal u geïnformeerd worden over deze nieuwe informatie die uw bereidwilligheid voor verdere deelname in deze studie kan beïnvloeden. In dit geval zal u gevraagd worden de nieuwe informatiebrochure en het nieuwe toestemmingsformulier te ondertekenen.

Ethische commissie

De onafhankelijke ethische commissie van UZ Leuven heeft deze studie beoordeeld en goedgekeurd.

Contactpersoon in geval van vragen omtrent de studie

Wanneer u denkt dat u schade hebt opgelopen gerelateerd aan de studie, een reactie hebt op de studiemedicatie of vragen hebt omtrent de studie kan u nu, tijdens en/of na de studie contact opnemen met de hoofdonderzoeker.

CONFIDENTIAL

Hoofdonderzoeker: Prof. dr. Diethard Monbaliu
Adres: Abdominale transplantatiechirurgie, UZ Leuven
Herestraat 49 bus 7003, 3000 Leuven
Telefoon secretariaat: + 32 16 348727
Fax secretariaat: + 32 16 348743

Studiecoördinator: Ilse Senesael
Adres: Abdominale transplantatiechirurgie, UZ Leuven
Herestraat 49 bus 7003, 3000 Leuven
Telefoon: + 32 16 342848

CONFIDENTIAL

Patient Information

Patient Number: ___________

Combined drug Approach to Prevent liver damage during Transplantation

You are invited to take part in a research study that tries to reduce the damage to the liver (the so called ischemia-reperfusion injury) during the transplantation process by using a multi-drug conditioning. Before you decide to participate in this study, it is important that you read this form. In this information and consent form, the purpose, the examinations, the advantages, risks and inconveniences coupled with this study are described. The right to withdraw your consent to participate at any time is also described below. No promises or guarantees can be made concerning the results of the study. You have the right to ask questions at any time, for example concerning the possible and/or known risks contained in this study.

Purpose and description of the study

Procurement, storage and transplantation can by themselves injure the liver. This damage starts because of the unavoidable oxygen deficiency during the storage of the liver (= time between the procurement of the liver and the transplantation) and is so called the ischemia-reperfusion injury (IRI). The extent of IRI can cause minimal to severe injury or even total destruction of the graft. Decreasing IRI is therefore crucial.
The process of IRI is composed of a whole mechanism of processes. The reduction of the IRI by using a multi-drug conditioning is based on the attenuation of these processes so that the chance is maximal to reduce the process of IRI.
The combination of medications will be administered for the first time in men in a well-controlled environment during your liver transplantation, whereby continuous monitoring of the blood pressure, rhythm and temperature is.
The objective of this study is to demonstrate the safety of the combined drug approach.
This is a safety study. If you accept to participate in this study, beside the standard treatment of care, you will receive the multi-drug conditioning.

The composition of the multi-drug conditioning is defined as:

- *Cetor®/Cinryze®*: C1-esterase inhibitor. It plays a major role in the inflammatory reaction and blood coagulation.
- *Atenativ®*: anti-coagulation, anti-inflammation.
- Vitamine E suspension®: protects against the damaging effects of oxygen
- *Tationil® 600 and Circadin®*: protects against the damaging effects of oxygen
- *Apotransferrin*: protection against the damaging effects of oxygen and free iron

CONFIDENTIAL

- *Neorecormon*®: Protection against cell death and damaging effects of oxygen, diminution of inflammatory messengers
- *Remicade*®: Diminution of the inflammatory messengers.
- *Flolan*®: dilatation of the bloodvessels, anti-inflammatory and platelet activation inhibition.

10 patients will take part in this medical-research study.

Length of the study

The duration of the safety study will be 1 week.

Examinations

- Blood and urine samples will *routinely* be taken during 14 days after the liver transplantation on a daily basis. *Standard clinical* examinations at 3, 6, 12 months after transplantation are also foreseen at the outpatient control examinations during the consultation.
- Likewise, 2 liver biopsies are predicted:
  - 1 hour before the implantation
  - 1 hour after the reperfusion

Voluntary participation

Your participation to this study is entirely voluntary. You have the right to refuse. You will be treated and followed without any disadvantage and any regard to medical care or physician-patient relationship whatever your decision.
If you accept to participate in this study, you will be asked to sign the attached consent form.
It is your right to stop your participation at the study whenever you want even after you have signed the informed consent. You don't have to give a reason for withdrawing your consent to participate.
The study doctor can stop your participation in this study at any moment, even without having to request your consent.

Risks and inconveniences

You will receive only one dose of each component (except for 2 doses for Neorecormon®). One single administration of these substances will be investigated in this study. Unexpected interactions/ potential risks due to the combination of drugs cannot be completely excluded. During the treatment with pharmacological products, you can encounter side effects:

CONFIDENTIAL

- *Cetor®/Cinryze®*: Allergic or anaphylactic reactions (eg. Tachycardia, hyper- or hypotension, redness in the neck and face, hives (urticaria), dyspnea, headache, dizziness and nausea).
- *Atenativ®*: Allergic reactions or hypersensitivity, increase of body temperature, dilatation of the blood vessels.
- *Vitamine E suspension®:* no side effects.
- *Tationil® 600*: no known side effects.
- *Circadin®*: no side effects with the used dose.
- *Apotransferrin*: no known side effects.
- *Neorecormon®*: formation of a clot in a blood vessel, seizure and high blood pressure.
- *Remicade®*: skin reaction, abnormalities in blood, hypersensibility and threatening allergic reactions.
- *Flolan®*: headache, nausea, hypotension, low blood pressure, slow or rapid heart rate and dyspnea.

Advantages

Even if each component of the multi-drug conditioning is well known to reduce the severity of ischemia-reperfusion injury, we cannot certify a beneficial effect of the multi-drug conditioning.

Insurance

The sponsor of the study is responsible for all the damage that you and/or your rightful claimants incur that is directly or indirectly related with the study. This is in accordance with the Belgian Law concerning experiments on the human person of May 7, 2004. The sponsor has closed an insurance that will cover this liability. If you would incur damage because of your participation to the study, this damage will be compensated by the sponsor of this study in accordance with the Belgian Law concerning experiments on the human person of May 7, 2004.

Compensations

There will be NO extra costs for you. You don't have to pay components of the multidrug conditioning. Moreover, all the examinations on the samples (blood, urine and tissue) that are not considered as current and routine practices will be paid by the sponsor.

Protection of your private life

Your identity and your participation to this study will be treated as strictly confidential. You will not be identified by name or in any other identifying manner in files, results or publications concerning this study.

CONFIDENTIAL

According to the GCP (good clinical practice) guidelines, your medical records will be examined by representatives of the sponsor and by the regulating authorities in order to control the study. Your identity will remain secret since a unique patient number will only designate personal information.
Your personal information might be transferred to regulating authorities, to the ethics committee and to other doctors that cooperate with the sponsor.
Your personal information will be processed and analyzed electronically or manually in order to determine the results of this study. You have the right to ask to the study doctor which data are collected about you in the context of the study and what the purpose of that collection is. You also have the right to request the study doctor to give you access to your personal information and to correct it if necessary. The protection of personal data is legally established in the Law of December 8, 1992 (Belgium) concerning the protection of private life.

Notification of new information

Sometimes new information on the study treatment or medicinal product appears in the course of the research project. If this is the case, you will be informed about new information that might influence your willingness to further participate in this study. In that case you will be asked to sign new information and consent form.

Ethics committee

The independent ethics committee of UZ Leuven has reviewed and approved this study.

Contact persons in the case of questions concerning the study

If you think having incurred damage related to the study or having a reaction on the study medications, or if you have questions concerning the study or your rights as a participant, you can contact, now, during or after the study:

Principal investigator: Prof. dr. Diethard Monbaliu
Adress: Abdominal Transplant Surgery, UZ Leuven
Herestraat 49 bus 7003
3000 Leuven
Phone secretariat: +32 16 348727
Fax secretariat: +32 16 348743

Coordinator of clinical trials : Ilse Senesael
Adress: Abdominal Transplant Surgery, UZ Leuven
Herestraat 49 bus 7003, 3000 Leuven
Phone: +32 16 342848

CONFIDENTIAL

BROCHURE INFORMATIVE

Patient numéro: ___________

Réduction des lésions du foie en transplantation après administration séquentielle de médicaments (CAPITL)

Vous êtes convié à participer à une étude clinique dans laquelle une série de médicaments sera administrée afin de réduire les lésions du foie liées à la préservation (appelées lésions d'ischémie-reperfusion). Avant de prendre une décision quant à votre participation, il est capital de prendre connaissance du contenu de cette brochure. Dans celle-ci seront successivement exposés les buts, les analyses, les avantages, les risques et inconvénients inhérents à l'étude mais également votre droit de vous rétracter et d'annuler votre accord à n'importe quel moment du projet. Aucune garantie ne peut être promise quant aux résultats escomptés. Vous avez le droit de poser des questions quand vous le désirez. Nous sommes à votre entière disposition pour vous répondre.

Objectif et description de l'étude

Le prélèvement d'organe, sa préservation et la transplantation engendrent des lésions au foie. Cette détérioration est liée à l'absence de perfusion et d'oxygénation du foie durant la période entre le prélèvement d'organes du donneur et la greffe chez le receveur. Ces dommages sont nommés «lésions d'ischémie reperfusion». L'ampleur de ces lésions varie et peut être responsable d'une destruction totale de la greffe, d'où l'intérêt de les diminuer. L'apparition des lésions d'ischémie reperfusion est secondaire à une série d'évènements complexes.
L'avantage lié à l'utilisation concomitante de plusieurs médicaments résulte dans l'action simultanée de chaque produit sur une étape précise de cette cascade d'évènements, augmentant ainsi la possibilité de réduire ces lésions.
L'administration se fera de façon bien précise et contrôlée. Le suivi de vos paramètres cliniques tels que la pression artérielle, le rythme cardiaque et la température sera assuré de façon continue.
L'objectif de cette étude est donc de démontrer pour la première fois la diminution des lésions d'ischémie reperfusion grâce à l'administration séquentielle de ces médicaments. L'objectif ultime de cette étude est de démontrer la sécurité avec laquelle nous pouvons utiliser la combinaison de médicament. Si vous acceptez de participer à cette étude, outre le traitement standard dont bénéficient tous les patients greffés hépatiques actuellement, vous recevrez la combinaison de médicaments.

Les médicaments sont les suivants:

- Cetor®/Cinryze® : Inhibiteur du complément qui joue un rôle crucial dans la réaction inflammatoire et dans la coagulation.
- Atenativ® : Anti-thrombine-III qui a également un rôle prépondérant dans la réaction inflammatoire et dans la coagulation.

CONFIDENTIAL

- Vitamine E sirop®, Circadin®, Tationil® 600 : Protection contre les effets délétères liés à l'oxygène (antioxidant).
- Apotransferrin® : l'Apotransferrine permet la chélation du fer toxique outre son pouvoir antioxidant.
- Neorecormon® : Erythropoïetin qui permet de protéger la mort des cellules, qui diminue l'inflammation et possède un pouvoir antioxidant.
- Remicade® : Infliximab qui diminue la réaction inflammatoire.
- Flolan® : Epoprostenol qui est un vasodilatateur puissant. Il possède également des propriétes antioxidante et anti-aggrégante.

10 patients participeront à cette étude.

Durée de l'étude

La durée de l'étude est limitée à 1 semaine.

Analyses

- Exactement comme réalisé chez tous les patients greffés du foie, des échantillons de sang et d'urines seront prélevés une fois par jour durant les 14 premiers jours après la greffe de foie. Ces échantillons seront également réalisés comme d'habitude lors d'une consultation de contrôle à 3, 6, 12 mois après la transplantation.
- En outre, 2 biopsies de la greffe hépatique habituellement réalisées sont prévues:
    - Juste avant la greffe de foie,
    - 1 heure après la reperfusion de la greffe de foie,

Participation volontaire

Votre participation à l'étude dépend strictement de votre décision. Vous avez le droit de refuser d'y participer. Quelque soit votre décision, vous serez traités avec la plus grande attention sans aucune faveur ou privilège de la part des corps médical et paramédical.
Si vous acceptez de participer à l'étude, nous vous invitons à signer un consentement éclairé.
Durant toute la durée de l'étude et sans devoir vous justifier, il vous appartient d'annuler votre participation même si vous avez signé le consentement éclairé.
Par contre, le corps médical a également le droit de mettre fin à votre participation à n'importe quel moment dans devoir exiger votre accord.

Effets secondaires

CONFIDENTIAL

Vous allez recevoir une seule dose de chaque médicament (excepté le Néorecormon qui requière une seconde administration). Après une revue de la littérature importante, il s'est avéré que l'administration d'une dose unique de ces produits est dénuée d'effet secondaire sévère. Cependant, même si le risque de survenue est extrêmement faible, il n'est pas exclu d'en présenter.

- *Cetor*®/Cinryze®: réaction allergique ou anaphylactique (accélération du rythme cardiaque, chute de la tension artérielle, rougeur, urticaire, nausée, difficulté respiratoire)
- *Atenativ*®: réaction allergique, augmentation de la température corporelle, dilatation des vaisseaux sanguins.
- *Vitamine E sirop*®*:* Pas d'effet secondaire connu à la dose utilisée.
- *Tationil 600*®: Pas d'effet secondaire connu à la dose utilisée.
- *Melatonin*®: Pas d'effet secondaire connu à la dose utilisée.
- *Apotransferrin*®: Pas d'effet secondaire connu à la dose utilisée.
- *Neorecormon*®: formation de caillot, épilepsie, augmentation de la pression artérielle.
- *Remicade*®: Réaction cutanée, troubles sanguins, réactions allergique et anaphylactique.
- *Flolan*®: Chute de la pression artérielle, augmentation du rythme cardiaque, difficulté respiratoire.

Finalement, des effets secondaires inattendus liés à l'administration séquentielle de ces médicaments ne peuvent être complètement exclus.

Avantages

Chaque médicament inclus a prouvé son efficacité dans la réduction des lésions d'ischémie reperfusion. Leur administration simultanée durant la greffe de foie devrait avoir un effet bénéfique mais que nous ne pouvons prédire avec certitude un effet bénéfique.

Assurances

En accord avec la loi belge concernant les études cliniques du 7 mai 2004, il va de soi que le promoteur de l'étude est considéré comme étant responsable de tout dommage encouru qu'il soit directement ou indirectement lié à l'étude.

Frais

Il n'y aura aucun coût supplémentaire pour vous, aussi bien pour les médicaments inclus que pour tout échantillon (sang, urine et biopsie) considéré comme étant en dehors de la pratique clinique habituelle.

Anonymat-Protection de la vie privée

CONFIDENTIAL

En accord avec les directives GCP (Good Clinical Practice) et la loi de la protection de la vie privée du 8 décembre 1992, votre identité et votre participation à l'étude restera strictement confidentielle. Vous ne serez jamais identifié dans les données, les résultats ou publications liés à l'étude si ce n'est par un numéro unique qui désignera vos données personnelles. Toutes ces données seront traitées manuellement ou électroniquement.
Seuls les représentants du promoteur ainsi que les autorités compétentes pourront avoir accès à votre dossier dans l'unique but de contrôler l'étude. Vos données personnelles pourront donc être transmises aux autorités compétentes tel que le comité d'éthique ou à d'autres médecins participant dans le cadre de l'étude.
A tout moment, vous avez le droit de consulter vos données personnelles.

Information au sujet d'une modification

Vous serez informés de tout potentiel changement concernant l'étude ou les médicaments. Dans ce cas, vous serez invités à signer un nouveau consentement éclairé.

Comité d'éthique

Avant d'entreprendre cette étude, le comité d'éthique indépendant de l'UZ Leuven a analysé le protocole et a donné son accord pour le projet.

Personnes à contacter en cas de questions liées à l'étude

Si vous avez des questions concernant un potentiel dommage, vos données personnelles ou vos droits en tant que participant, n'hésitez pas à nous contacter que ce soit avant la greffe, durant ou après votre hospitalisation.

Investigateur principal: Prof. Dr. Diethard Monbaliu
Adresse: Abdominal Transplant Surgery, UZ Leuven
Herestraat 49 bus 7003
3000 Leuven
Téléphone secrétariat: +32 16 348727
Fax secrétariat: +32 16 348743

Coordonatrice des essais cliniques : Senesael Ilse

Adress : Abdominal Transplant Surgery, UZ Leuven
Herestraat 49 bus 7003
3000 Leuven
Téléphone : +32 16 342848

APPENDIX 8: PATIENT INFORMATION BROCHURE: Part B. Randomized Controlled Trial

Informatiebrochure

Toediening geneesmiddelen om leverbeschadiging tijdens transplantatie te verminderen

Patiënten nr:

Sponsor: UZ Leuven, Herestraat 49, 3000 Leuven

U wordt gevraagd om deel te nemen aan een klinische studie waarbij verschillende medicijnen tijdens uw levertransplantatie toegediend zullen worden met als doel de beschadiging van de lever tijdens het transplantatie proces (zogenoemde ischemie-reperfusie schade) te verminderen. Deze studie heeft UZ KU Leuven als opdrachtgever en zal plaatsvinden in verschillende nationale en internationale ziekenhuizen. Het is belangrijk om dit document te lezen alvorens u beslist om aan deze studie deel te nemen. De doelstellingen, onderzoeken, voor- en nadelen, risico’s en ongemakken die aan deze studie verbonden zijn, worden in de informatiebrochure en het toestemmingsformulier beschreven. Er kan niets worden beloofd noch worden gegarandeerd in verband met de resultaten van de studie. U hebt het recht om, om het even wanneer, vragen te stellen met betrekking tot de mogelijke en/of bestaande risico’s in deze studie.

Doelstelling en beschrijving van de studie

Tijdens het chirurgisch verwijderen, bewaren en transplanteren van een lever kan dit orgaan beschadigd worden. Deze beschadiging is deels het gevolg van een onvermijdbaar zuurstoftekort tijdens de bewaring van de lever na het chirurgisch verwijderen tot de transplantatie(ook ischemie genoemd).
Deze ischemische schade kan echter na de levertransplantatie versterkt worden wanneer de bloeddoorstroming in de ontvanger hersteld wordt (dit noemt men ischemie-reperfusie schade). De opgelopen orgaanschade kan minimaal maar kan ook zeer ernstig tot levensbedreigend zijn. Een vermindering van de ischemie-reperfusie schade is dan ook van groot belang omdat het de resultaten van levertransplantatie kan verbeteren.
Het proces van ischemie-reperfusie schade bestaat uit een complexe cascade van elkaar versterkende processen.
Het doel van deze studie is dan ook ischemie-reperfusie schade na levertransplantatie te verminderen tot minimaliseren.

CONFIDENTIAL

Om al deze schadelijke processen te blokkeren, voorzien we om verschillende geneesmiddelen toe te dienen zodat de kans op succes zo groot mogelijk is. Deze combinatie aan geneesmiddelen zal voor het eerst bij de mens worden gebruikt en wordt tijdens de levertransplantatie toegediend. Ondertussen zullen uw bloeddruk, hartritme en temperatuur voortdurend gemeten worden.
Het doel van deze studie is de doeltreffendheid van deze geneesmiddelen in het verminderen van ischemie-reperfusie schade aan te tonen. Het gaat om een gerandomiseerde en gecontroleerde studie. In een eerste zogenomende “safety, fase A” van deze studie, konden geen ernstige nevenwerkingen aangetoond worden van de gecombineerde toediening van al deze geneesmiddelen. Wanneer u besluit om aan deze studie deel te nemen, zal u naast de standaardbehandeling willekeurig aan al dan niet de bijkomende toegediende geneesmiddelen zoals voorzien in deze studie worden onderworpen.
De geneesmiddelen die toegediend zullen worden, zijn:

- *Cetor®/Cinryze®*: C1-esterase inhibitor. C1-esterase inhibitor speelt een belangrijke rol in de onstekingsreactie en bloedstolling.
- *Atenativ®*: anti-stolling, anti-ontsteking
- *Vitamin E oplossing®*: Vitamine E biedt bescherming tegen de schadelijke effecten van zuurstof
- *Tationil® 600 en Circadin®*: biedt bescherming tegen de schadelijke effecten van zuurstof
- *Apotransferrine*: houdt ijzer vast en biedt ook bescherming tegen de schadelijke effecten van zuurstof
- *Neorecormon®*: biedt bescherming tegen celdood en de schadelijke effecten van zuurstof, zorgt voor een vermindering van ontstekingssignalen
- *Remicade®*: vermindering van ontstekingssignalen
- *Flolan®*: anti-ontsteking, blokkeren van de activatie van bloedplaatjes en verwijding van de bloedvaten

Er zullen 72 patiënten aan deze studie deelnemen. De randomisatie gebeurt door een derde die niet bij de studie betrokken is. Patiënten zullen in ieder deelnemend centrum gerandomiseerd worden in twee groepen door gebruik te maken van gepermuteerde blokken van variabele grootte. -

Tijdsduur studie

De studie zal één jaar duren.

Onderzoeken

- Zoals iedereen die een levertransplantatie ondergaat, zullen er dagelijks en <u>routinematig</u> bloed- en urinestalen worden afgenomen de eerste 14 dagen na levertransplantatie. Ook <u>standaard klinische onderzoeken</u> zullen plaatsvinden en dit 3, 12 maanden na de transplantatie op de raadpleging.
- Bovendien worden ook 3 leverbiopsies genomen:

CONFIDENTIAL

- 1 uur voor de implantatie
- 1 uur na de reperfusie
- 7 dagen na de transplantatie (deze behoort niet tot de routine)

Naast de leverbiopsies, worden ook twee biopsies van de galwegen en 1 collectie van de gal afgenomen en dit tijdens de levertransplantatie.

- Een jaar na de transplantatie zal er routinematig een MRI (Magnetic Resonance Imaging) gepland worden om de kwaliteit van de getransplanteerde lever na te gaan.

Vrijwillige deelname

Uw deelname aan deze studie is volledig vrijwillig. U hebt het recht om deelname te weigeren. Wat u ook beslist, u zal behandeld en opgevolgd worden zonder enig nadelig gevolg voor uw medische zorgen en voor uw relatie met uw behandelende arts (dokter-patiënt relatie).
Wanneer u beslist om aan deze studie deel te nemen, dan zal er aan u gevraagd worden het toestemmingsformulier te ondertekenen.
Nadat u het toestemmingsformulier hebt ondertekend is het uw recht om uw deelname aan de studie te stoppen om het even wanneer, U moet geen reden geven waarom u uw toestemming voor deelname aan de studie intrekt.
De studiedokter kan op elk moment uw deelname aan de studie stopzetten en dit zonder uw toestemming te vragen.

Risico’s en ongemakken

Er zal maar één dosis (twee van EPO) van ieder geneesmiddel in deze studie worden toegediend. Na het uitvoerig raadplegen van de medische literatuur, blijkt een eenmalige toediening van deze componenten veilig te zijn. Dit blijkt ook uit de resultaten van de safety studie. Toch kunnen onverwachte interacties/potentiële risico’s eigen aan de combinatie niet helemaal uitgesloten worden. U kan ook bijwerkingen tijdens de behandeling met deze medicijnen ondervinden, namelijk:

- *Cetor®/Cinryze®*: Allergische of anafylactische reacties (bijvoorbeeld: versneld hartritme, hoge of lage bloeddruk, roodheid in de hals en in het gezicht, netelroos, kortademigheid)
- *Atenativ®*: Allergische reacties of overgevoeligheid, toename in lichaamstemperatuur en verwijdering van de bloedvaten
- *Vitamine E oplossing®*: geen gekende bijwerkingen
- *Tationil® 600*: geen gekende bijwerkingen
- *Circadin®*: geen gekende bijwerkingen
- *Apotransferrine*: geen gekende bijwerkingen
- *Neorecormon®*: vorming van een bloedklonter in een bloedvat, epilepsie en hoge bloeddruk

CONFIDENTIAL

- *Remicade*: huiduitslag, afwijkingen in de samenstelling van bloed overgevoeligheid en levensbedreigende allergische reacties
- *Flolan*®: hoofdpijn, misselijkheid, lage bloeddruk, traag hartritme, versneld hartritme en kortademigheid

Voordelen

We kunnen het therapeutisch effect van de geneesmiddelen niet verzekeren zelfs al weten we dat ieder geneesmiddel op zich de intensiteit van ischemie-reperfusie schade vermindert.

Verzekering

Conform de Belgische wet inzake experimenten op de menselijke persoon van 7 mei 2004 is de sponsor van het onderzoek, zelfs foutloos, aansprakelijk voor alle schade die de deelnemer en/of zijn rechthebbenden oplopen en die rechtstreeks dan wel onrechtstreeks verband houdt met de proef. De sponsor heeft een verzekering afgesloten die deze aansprakelijkheid dekt. Indien u schade zou oplopen ten gevolge van uw deelname aan deze studie, zal die schade bijgevolg worden vergoed conform de Belgische wet inzake experimenten op de menselijke persoon van 7 mei 2004.

Compensaties

Deze studie brengt voor U geen extra kosten met zich mee. U hoeft de geneesmiddelen ook niet te betalen. Bovendien worden alle onderzoeken op stalen (bloed-, urine- en weefselstalen) die niet beschouwd worden als courant en routinematig, betaald door de onderzoeker.

Bescherming van uw privéleven

Uw identiteit en deelname aan deze studie zullen strikt vertrouwelijk behandeld worden. U zal niet geïdentificeerd kunnen worden bij naam of op enige andere manier in documenten, resultaten of publicaties betreffende de studie.
Uw medische gegevens zullen onderzocht worden door vertegenwoordigers van de onderzoeker en door regulerende instanties die de studie ook controleren. Dit gebeurt volgende de GCP (= Good Clinical Practice) richtlijnen. Hierbij wordt Uw identiteit geheim gehouden door een uniek patiëntennummer te gebruiken om Uw persoonlijke informatie aan te duiden.
Uw persoonlijke informatie zal doorgegeven worden aan de regulerende instanties, de ethische commissie en andere dokters die samenwerken met de onderzoeker.
Uw persoonlijke informatie zal elektronisch of manueel verwerkt en geanalyseerd worden om de resultaten van deze studie te bepalen. U hebt het recht aan de dokter van de studie te vragen naar uw data die verzameld zijn en de doelstelling van deze verzameling. U hebt ook het recht om de studiedokter te verzoeken om u toegang te geven tot uw persoonlijke informatie en die te verbeteren indien nodig.

CONFIDENTIAL

Alle reglementering betreffende de bescherming van de persoonlijke data is terug te vinden in de Wet van 8 december 1992 (België), betreffende de bescherming van het privéleven.

Nieuwe informatie

Soms is het mogelijk dat nieuwe informatie omtrent behandeling of medicinale producten in de context van het klinisch project verschijnt. Indien dit het geval is, zal u geïnformeerd worden over deze nieuwe informatie die uw bereidwilligheid voor verdere deelname in deze studie kan beïnvloeden. In dit geval zal u gevraagd worden de nieuwe informatiebrochure en het nieuwe toestemmingsformulier te ondertekenen.

Ethische commissie

De onafhankelijke Commissie Medische Ethiek van UZ KU Leuven/Onderzoek heeft deze studie beoordeeld en goedgekeurd, evenals de lokale Commissies voor Medische Ethiek van de centra waar deze studie doorgaat.

Contactpersoon in geval van vragen omtrent de studie

Wanneer u denkt dat u schade hebt opgelopen gerelateerd aan de studie, een reactie hebt op de studiemedicatie of vragen hebt omtrent de studie kan u nu, tijdens en/of na de studie contact opnemen met de hoofdonderzoeker.

Hoofdonderzoeker: Prof. dr. Diethard Monbaliu
Adres: Abdominale transplantatiechirurgie, UZ Leuven
Herestraat 49 bus 7003, 3000 Leuven
Telefoon secretariaat: + 32 16 348727
Fax secretariaat: + 32 16 348743

Studiecoördinator: Sarah Mertens
Adres: Abdominale transplantatiechirurgie, UZ Leuven
Herestraat 49 bus 7003, 3000 Leuven
Telefoon: + 32 16 342961

CONFIDENTIAL

Patient Information

Patient Number: ___________

Sponsor: University Hospitals Leuven, Herestraat 49, 3000 Leuven

Combined drug Approach to Prevent liver damage during Transplantation

You are invited to take part in a research study that tries to reduce the damage to the liver (the so called ischemia-reperfusion injury) during the transplantation process by using a multi-drug conditioning. This study has UZ KU Leuven as sponsor and will take place in different national and international hospitals. Before you decide to participate in this study, it is important that you read this form. In this information and consent form, the purpose, the examinations, the advantages, risks and inconveniences coupled with this study are described. The right to withdraw your consent to participate at any time is also described below. No promises or guarantees can be made concerning the results of the study. You have the right to ask questions at any time, for example concerning the possible and/or known risks contained in this study.

Purpose and description of the study

Procurement, storage and transplantation can by themselves injure the liver. This damage starts because of the unavoidable oxygen deficiency during the storage of the liver (= time between the procurement of the liver and the transplantation) and is so called the ischemia-reperfusion injury (IRI). The extent of IRI can cause minimal to severe injury or even total destruction of the graft. Decreasing IRI is therefore crucial.
The process of IRI is composed of a whole mechanism of processes. The reduction of the IRI by using a multi-drug conditioning is based on the attenuation of these processes so that the chance is maximal to reduce the process of IRI.
The combination of medications will be administered for the first time in men in a well-controlled environment during your liver transplantation, whereby continuous monitoring of the blood pressure, rhythm and temperature is.
The objective of this study is to demonstrate the efficacy of the combined drug approach in reducing the IRI.
This is a randomized, controlled and single center study. In the safety phase, phase A of this study, no serious adverse events were observed. . If you accept to participate in this study, beside the standard treatment of care, you will be randomly assigned to receive or not the multi-drug conditioning.

CONFIDENTIAL

The composition of the multi-drug conditioning is defined as:

- *Cetor®/Cinryze®*: C1-esterase inhibitor. It plays a major role in the inflammatory reaction and blood coagulation.
- *Atenativ®*: anti-coagulation, anti-inflammation.
- Vitamine E suspension®: protects against the damaging effects of oxygen
- *Tationil® 600 and Circadin®:* protects against the damaging effects of oxygen
- *Apotransferrin*: protection against the damaging effects of oxygen and free iron
- *Neorecormon®*: Protection against cell death and damaging effects of oxygen, diminution of inflammatory messengers
- *Remicade®*: Diminution of the inflammatory messengers.
- *Flolan®*: dilatation of the bloodvessels, anti-inflammatory and platelet activation inhibition.

72 patients will take part in this medical-research study. The randomization will be done by a third party, who is not involved in the study. In each participating center, patients will be randomized into two groups using permuted blocks of variable size.

Length of the study

The duration of the study will be 1 years.

Examinations

- Blood and urine samples will *routinely* be taken during 14 days after the liver transplantation on a daily basis. *Standard clinical* examinations at 3, 12 months after transplantation are also foreseen at the outpatient control examinations during the consultation.
- Likewise, 3 liver biopsies are predicted:
  - 1 hour before the implantation
  - 1 hour after the reperfusion
  - 7 days after the transplantation (doesn't belong to the routine tests)

Besides the liver biopsies, 2 biopsies of the bile duct and 1 bile collection will be taken during the liver transplantation.

As done routinely, an MRI (magnetic resonance imaging) scan will be planned one year after the transplantation to evaluate the quality of the liver, its blood supply and the bile ducts.

Voluntary participation

CONFIDENTIAL

Your participation to this study is entirely voluntary. You have the right to refuse. You will be treated and followed without any disadvantage and any regard to medical care or physician-patient relationship whatever your decision.
If you accept to participate in this study, you will be asked to sign the attached consent form.
It is your right to stop your participation at the study whenever you want even after you have signed the informed consent. You don't have to give a reason for withdrawing your consent to participate.
The study doctor can stop your participation in this study at any moment, even without having to request your consent.

Risks and inconveniences

You will receive only one dose of each component (except for 2 doses for EPO). After a review of the literature and research to the safety in the safety study, one single administration of these substances is safe. However, unexpected interactions/ potential risks due to the combination of drugs cannot be completely excluded. During the treatment with pharmacological products, you can also encounter side effects:

- *Cetor®/Cinryze®*: Allergic or anaphylactic reactions (eg. Tachycardia, hyper- or hypotension, redness in the neck and face, hives (urticaria), dyspnea, headache, dizziness and nausea).
- *Atenativ®*: Allergic reactions or hypersensitivity, increase of body temperature, dilatation of the blood vessels.
- *Vitamine E suspension®*: no side effects.
- *Tationil® 600*: no known side effects.
- *Circadin®*: no side effects with the used dose.
- *Apotransferrin*: no known side effects.
- *Neorecormon®*: formation of a clot in a blood vessel, seizure and high blood pressure.
- *Remicade®*: skin reaction, abnormalities in blood, hypersensibility and threatening allergic reactions.
- *Flolan®*: headache, nausea, hypotension, low blood pressure, slow or rapid heart rate and dyspnea.

Advantages

Even if each component of the multi-drug conditioning is well known to reduce the severity of ischemia-reperfusion injury, we cannot certify a beneficial effect of the multi-drug conditioning.

Insurance

The sponsor of the study is responsible for all the damage that you and/or your rightful claimants incur that is directly or indirectly related with the study. This is in accordance with the Belgian Law concerning experiments on the human person of May 7, 2004. The

CONFIDENTIAL

sponsor has closed an insurance that will cover this liability. If you would incur damage because of your participation to the study, this damage will be compensated by the sponsor of this study in accordance with the Belgian Law concerning experiments on the human person of May 7, 2004.

Compensations

There will be NO extra costs for you. You don't have to pay components of the multidrug conditioning. Moreover, all the examinations on the samples (blood, urine and tissue) that are not considered as current and routine practices will be paid by the sponsor.

Protection of your private life

Your identity and your participation to this study will be treated as strictly confidential. You will not be identified by name or in any other identifying manner in files, results or publications concerning this study.
According to the GCP (good clinical practice) guidelines, your medical records will be examined by representatives of the sponsor and by the regulating authorities in order to control the study. Your identity will remain secret since a unique patient number will only designate personal information.
Your personal information might be transferred to regulating authorities, to the ethics committee and to other doctors that cooperate with the sponsor.
Your personal information will be processed and analyzed electronically or manually in order to determine the results of this study. You have the right to ask to the study doctor which data are collected about you in the context of the study and what the purpose of that collection is. You also have the right to request the study doctor to give you access to your personal information and to correct it if necessary. The protection of personal data is legally established in the Law of December 8, 1992 (Belgium) concerning the protection of private life.

Notification of new information

Sometimes new information on the study treatment or medicinal product appears in the course of the research project. If this is the case, you will be informed about new information that might influence your willingness to further participate in this study. In that case you will be asked to sign new information and consent form.

Ethics committee

The independent Medical Ethics Committee of the University Hospitals KU Leuven / Research has assessed and approved this study, as well as the local Medical Ethics Committees of the other participating centers where this study is taking place.

Contact persons in the case of questions concerning the study

CONFIDENTIAL

If you think having incurred damage related to the study or having a reaction on the study medications, or if you have questions concerning the study or your rights as a participant, you can contact, now, during or after the study:

Principal investigator: Prof. dr. Diethard Monbaliu
Adress: Abdominal Transplant Surgery, UZ Leuven
Herestraat 49 bus 7003
3000 Leuven
Phone secretariat: +32 16 348727
Fax secretariat: +32 16 348743

Coordinator of clinical trials : Sarah Mertens
Adress: Abdominal Transplant Surgery, UZ Leuven
Herestraat 49 bus 7003, 3000 Leuven
Phone: +32 16 342961

CONFIDENTIAL

BROCHURE INFORMATIVE

Patient numéro: ___________

Promoteur: UZ Leuven, Herestraat 49, 3000 Leuven

Réduction des lésions du foie en transplantation après administration séquentielle de médicaments (CAPITL)

Vous êtes convié à participer à une étude clinique dans laquelle une série de médicaments sera administrée afin de réduire les lésions du foie liées à la préservation (appelées lésions d'ischémie-reperfusion). Cette étude a comme principal UZ KU Leuven et se poursuivra dans des centres hospitaliers nationaux et internationaux différents. Avant de prendre une décision quant à votre participation, il est capital de prendre connaissance du contenu de cette brochure. Dans celle-ci seront successivement exposés les buts, les analyses, les avantages, les risques et inconvénients inhérents à l'étude mais également votre droit de vous rétracter et d'annuler votre accord à n'importe quel moment du projet. Aucune garantie ne peut être promise quant aux résultats escomptés. Vous avez le droit de poser des questions quand vous le désirez. Nous sommes à votre entière disposition pour vous répondre.

Objectif et description de l'étude

Le prélèvement d'organe, sa préservation et la transplantation engendrent des lésions au foie. Cette détérioration est liée à l'absence de perfusion et d'oxygénation du foie durant la période entre le prélèvement d'organes du donneur et la greffe chez le receveur. Ces dommages sont nommés «lésions d'ischémie reperfusion». L'ampleur de ces lésions varie et peut être responsable d'une destruction totale de la greffe, d'où l'intérêt de les diminuer. L'apparition des lésions d'ischémie reperfusion est secondaire à une série d'évènements complexes.
L'avantage lié à l'utilisation concomitante de plusieurs médicaments résulte dans l'action simultanée de chaque produit sur une étape précise de cette cascade d'évènements, augmentant ainsi la possibilité de réduire ces lésions.
L'administration se fera de façon bien précise et contrôlée. Le suivi de vos paramètres cliniques tels que la pression artérielle, le rythme cardiaque et la température sera assuré de façon continue.
L'objectif de cette étude est donc de démontrer pour la première fois la diminution des lésions d'ischémie reperfusion grâce à l'administration séquentielle de ces médicaments.

CONFIDENTIAL

Il s’agit d’une étude randomisée et contrôlée. Pendant la phase de cette étude de sécurité, phase A, aucun effet secondaire grave n’a pu être démontré. C'est-à-dire que si vous acceptez de participer à cette étude, outre le traitement standard dont bénéficient tous les patients greffés hépatiques actuellement, vous serez réparti dans un des deux groupes qui recevra les médicaments ou non.

Les médicaments sont les suivants:

- Cetor®/Cinryze® : Inhibiteur du complément qui joue un rôle crucial dans la réaction inflammatoire et dans la coagulation.
- Atenativ® : Anti-thrombine-III qui a également un rôle prépondérant dans la réaction inflammatoire et dans la coagulation.
- Vitamine E sirop®, Circadin®, Tationil® 600 : Protection contre les effets délétères liés à l’oxygène (antioxidant).
- Apotransferrin® : l’Apotransferrine permet la chélation du fer toxique outre son pouvoir antioxidant.
- Neorecormon® : Erythropoïetin qui permet de protéger la mort des cellules, qui diminue l’inflammation et possède un pouvoir antioxidant.
- Remicade® : Infliximab qui diminue la réaction inflammatoire.
- Flolan® : Epoprostenol qui est un vasodilatateur puissant. Il possède également des propriétes antioxidante et anti-aggrégante.

72 patients participeront à cette étude. La randomisation se fait par un tiers qui n'est pas impliqué dans l'étude. Les patients seront mis en place dans chaque centre participant, randomisés en deux groupes par l'utilisation de blocs permutés taille variable.

Durée de l’étude

La durée de l’étude est limitée à un année.

Analyses

- Exactement comme réalisé chez tous les patients greffés du foie, des échantillons de sang et d’urines seront prélevés une fois par jour durant les 14 premiers jours après la greffe de foie. Ces échantillons seront également réalisés comme d’habitude lors d’une consultation de contrôle à 3, 12 mois après la transplantation.
- En outre, 3 biopsies de la greffe hépatique habituellement réalisées sont prévues:
    - Juste avant la greffe de foie,
    - 1 heure après la reperfusion de la greffe de foie,
    - 7 jours après la transplantation (pas de façon routinière),

CONFIDENTIAL

- En plus des biopsies du foie, 2 biopsies de la voie biliaire et une collection de bile seront prises au cours de la transplantation du foie.

- Classiquement, une résonance magnétique nucléaire (RMN) est réalisée afin d'étudier l'intégrité de l'arbre biliaire 1 an après la transplantation.

Participation volontaire

Votre participation à l'étude dépend strictement de votre décision. Vous avez le droit de refuser d'y participer. Quelque soit votre décision, vous serez traités avec la plus grande attention sans aucune faveur ou privilège de la part des corps médical et paramédical.
Si vous acceptez de participer à l'étude, nous vous invitons à signer un consentement éclairé.
Durant toute la durée de l'étude et sans devoir vous justifier, il vous appartient d'annuler votre participation même si vous avez signé le consentement éclairé.
Par contre, le corps médical a également le droit de mettre fin à votre participation à n'importe quel moment dans devoir exiger votre accord.

Effets secondaires

Vous allez recevoir une seule dose de chaque médicament (excepté le Néorecormon qui requière une seconde administration). Après une revue de la littérature importante et l'analyse des résultats d'une étude évaluant la sécurité de l'administration des médicaments, il s'est avéré qu'une seule dose de ces produits est dénuée d'effet secondaire sévère. Cependant, même si le risque de survenue est extrêmement faible, il n'est pas exclu d'en présenter.

- *Cetor*®/Cinryze®: réaction allergique ou anaphylactique (accélération du rythme cardiaque, chute de la tension artérielle, rougeur, urticaire, nausée, difficulté respiratoire)
- *Atenativ*®: réaction allergique, augmentation de la température corporelle, dilatation des vaisseaux sanguins.
- *Vitamine E sirop*®*:* Pas d'effet secondaire connu à la dose utilisée.
- *Tationil 600*®: Pas d'effet secondaire connu à la dose utilisée.
- *Melatonin*®: Pas d'effet secondaire connu à la dose utilisée.
- *Apotransferrin*®: Pas d'effet secondaire connu à la dose utilisée.
- *Neorecormon*®: formation de caillot, épilepsie, augmentation de la pression artérielle.
- *Remicade*®: Réaction cutanée, trouble sanguin, réactions allergique et anaphylactique.
- *Flolan*®: Chute de la pression artérielle, augmentation du rythme cardiaque, difficulté respiratoire.

Finalement, des effets secondaires inattendus liés à l'administration séquentielle de ces médicaments ne peuvent être complètement exclus.

CONFIDENTIAL

Avantages

Chaque médicament inclus a prouvé son efficacité dans la réduction des lésions d'ischémie reperfusion. Leur administration simultanée durant la greffe de foie devrait avoir un effet bénéfique mais que nous ne pouvons prédire avec certitude un effet bénéfique.

Assurances

En accord avec la loi belge concernant les études cliniques du 7 mai 2004, il va de soi que le promoteur de l'étude est considéré comme étant responsable de tout dommage encouru qu'il soit directement ou indirectement lié à l'étude.

Frais

Il n'y aura aucun coût supplémentaire pour vous, aussi bien pour les médicaments inclus que pour tout échantillon (sang, urine et biopsie) considéré comme étant en dehors de la pratique clinique habituelle.

Anonymat-Protection de la vie privée

En accord avec les directives GCP (Good Clinical Practice) et la loi de la protection de la vie privée du 8 décembre 1992, votre identité et votre participation à l'étude restera strictement confidentielle. Vous ne serez jamais identifié dans les données, les résultats ou publications liés à l'étude si ce n'est par un numéro unique qui désignera vos données personnelles. Toutes ces données seront traitées manuellement ou électroniquement.
Seuls les représentants du promoteur ainsi que les autorités compétentes pourront avoir accès à votre dossier dans l'unique but de contrôler l'étude. Vos données personnelles pourront donc être transmises aux autorités compétentes tel que le comité d'éthique ou à d'autres médecins participant dans le cadre de l'étude.
A tout moment, vous avez le droit de consulter vos données personnelles et de demander que des corrections éventuelles y soient apportées.

Information au sujet d'une modification

Vous serez informés de tout potentiel changement concernant l'étude ou les médicaments. Dans ce cas, vous serez invités à signer un nouveau consentement éclairé.

Comité d'éthique

Le Comité d'éthique médicale indépendant de la KU/UZ Leuven, ainsi que les comités locaux d'éthique médicale des centres où cette étude se poursuit, ont évalué et approuvé l'étude. En aucun cas vous ne devez considérer cet avis favorable comme une incitation à participer à cette étude.

Personnes à contacter en cas de questions liées à l'étude

CONFIDENTIAL

Si vous avez des questions concernant un potentiel dommage, vos données personnelles ou vos droits en tant que participant, n'hésitez pas à nous contacter que ce soit avant la greffe, durant ou après votre hospitalisation.

Investigateur principal: Prof. Dr. Diethard Monbaliu
Adresse: Abdominal Transplant Surgery, UZ Leuven
Herestraat 49 bus 7003
3000 Leuven
Téléphone secrétariat: +32 16 348727
Fax secrétariat: +32 16 348743

Coordonatrice des essais cliniques : Sarah Mertens

Adress : Abdominal Transplant Surgery, UZ Leuven
Herestraat 49 bus 7003
3000 Leuven
Téléphone : +32 16 342961

CONFIDENTIAL

Informationsbroschüre

Medikamentöse Strategien zur Reduktion des während einer Lebertransplantation auftretenden Leberschadens

Patienten-Nr:

Sponsor: University Hospitals Leuven, Herestraat 49, 3000 Leuven

Sie werden gebeten, sich an einer klinischen Studie zu beteiligen, bei der mehrere Medikamente mit dem Ziel verabreicht werden, die während einer Transplantation auftretenden Leberschäden (den so genannten Ischämie-Reperfusions-Schaden) zu reduzieren. Auftraggeber dieser Studie ist UZ KU Leuven. Die Studie wird in mehreren nationalen und internationalen Krankenhäusern durchgeführt.
Lesen Sie dieses Dokument bitte sorgfältig durch, bevor Sie beschließen, ob Sie an der Studie teilnehmen möchten. Die Ziele, notwendigen Untersuchungen, Vor- und Nachteile, Risiken und Unannehmlichkeiten, die mit dieser Studie verbunden sind, werden in der Informationsbroschüre und in der Einwilligungserklärung ausführlich beschrieben. Die Studie ist ergebnisoffen. Sie haben jederzeit das Recht, Fragen zu stellen über die möglichen und bestehenden Risiken, die mit einer Teilnahme an dieser Studie verbunden sind.

Zielsetzung und Beschreibung der Studie

Während der chirurgischen Entfernung (Explantation), der Konservierung und der Transplantation der Leber kann dieses Organ beschädigt werden. Diese Beschädigung ist zum Teil das Resultat des Sauerstoffmangels (auch als Ischämie bezeichnet), der im Zeitraum der Konservierung der Leber (zwischen der chirurgischen Entfernung des Spenderorgans bis hin zur Transplantation) unvermeidbar ist.
Der Ischämie-Schaden kann nach Wiederherstellung der Blutversorgung der Leber im Empfänger sogar noch zunehmen (dieses wird als Ischämie-Reperfusionsschaden bezeichnet). Der auftretende Organschaden kann minimal sein, ist aber in seltenen Fällen auch ernst oder sogar lebensbedrohlich. Eine Verringerung des Ischämie-Reperfusionsschadens ist daher von großer Bedeutung, weil es die Erfolgsaussichten der Lebertransplantation verbessern kann.

Der Ischämie-Reperfusionsschaden wird durch eine komplexe Kaskade von sich gegenseitig verstärkenden Prozessen verursacht.
Der Zweck dieser Studie ist es daher, den Ischämie-Reperfusionsschaden nach einer

CONFIDENTIAL

Lebertransplantation zu vermindern oder sogar minimieren.

Um all diese schädlichen Prozesse zu blockieren, planen wir, verschiedene Medikamente zu verabreichen, so dass die Wahrscheinlichkeit für einen Erfolg so groß wie möglich ist. Diese Medikamenten-Kombination wird zum ersten Mal am Menschen angewendet und während der Lebertransplantation verabreicht werden. Während der Medikamentengabe werden Ihr Blutdruck, Ihre Herzfrequenz und Ihre Temperatur kontinuierlich gemessen.

Das Ziel dieser Studie ist es, die Wirksamkeit dieser Medikamente zur Verringerung des Ischämie-Reperfusionsschadens zu demonstrieren. Es handelt sich um eine randomisierte, kontrollierte Studie. In einer ersten, sogenannten "Sicherheit, Phase A" dieser Studie konnten keine ernsthaften Nebenwirkungen der kombinierten Verabreichung dieser Arzneimittel beobachtet werden.
Wenn Sie sich entscheiden sollten, an dieser Studie teilzunehmen, wird per Los entschieden („Randomisation“), ob oder ob nicht bei Ihnen zusätzlich zur Standardbehandlung die Kombination der folgenden Medikamente verabreicht wird:
- *Cetor®/Cinryze®*: C1-Esterase-Hemmstoff. Der C1-Esterase-Hemmstoff spielt eine wichtige Rolle in der Entzündungsreaktion und Blutgerinnung.
- *Atenativ®*: Hemmstoff der Blutgerinnung, entzündungshemmend.
- *Vitamin E Lösung®*: Vitamin E schützt vor den schädlichen Auswirkungen von Sauerstoff.
- *Tationil® 600 und Circadin®*: Schützen vor den schädlichen Auswirkungen von Sauerstoff.
- *Apotransferrin*: Bindet Eisen und bietet auch Schutz vor den schädlichen Auswirkungen von Sauerstoff.
- *Neorecormon®*: Bietet Schutz gegen Zelltod und die schädlichen Auswirkungen von Sauerstoff, führt zu einer Verringerung der Entzündung-Signale.
- *Remicade®*: Verringerung der Entzündung-Signale.
- *Flolan®*: Entzündungshemmend, blockiert die Aktivierung von Blutplättchen und erweitert Blutgefäße.

72 Patienten werden an dieser Studie teilnehmen. Die Randomisierung wird durch eine unabhängige Person vollzogen, die nicht in die Studie involviert ist. Die Patienten werden in jedem Teilnehmerzentrum durch die Verwendung von permutierten Blöcken variabler Größe in zwei Gruppen randomisiert.

Zeitdauer der Studie

Die Studie wird ein Jahr dauern.

Untersuchungen

- Wie bei jeder Lebertransplantation werden in den ersten 14 Tagen nach der Transplantation täglich und routinemäßig Blut- und Urinproben abgenommen werden. 3 und 12 Monate nach der Lebertransplantation werden standardmäßig klinische Untersuchungen zur Nachbeobachtung durchgeführt.
- Darüber hinaus werden auch drei Leber-Biopsien durchgeführt, die zum großen Teil Standard bei einer Lebertransplantation sind:
  - 1 Stunde vor der Implantation
  - von 1 Stunde nach der Reperfusion

CONFIDENTIAL

- 7 Tage nach der Transplantation (dies ist nicht Teil der Routine)

- Zusätzlich zu den Leber-Biopsien, werden auch zwei Biopsien der Gallengänge und Probe des Gallensaftes abgenommen (während der Lebertransplantation).

-Ein Jahr nach der Transplantation wird routinemäßig ein MRT (Kernspin-Tomogramm) durchgeführt, um die Qualität der transplantierten Leber zu überprüfen.

Freiwillige Teilnahme

Ihre Teilnahme an dieser Studie ist vollkommen freiwillig. Sie haben das Recht, die Teilnahme zu verweigern. Auch im Falle einer Ablehnung der Teilnahme erhalten Sie eine vollwertige medizinische Behandlung, und müssen keine nachteiligen Auswirkungen auf Ihre medizinische Versorgung und die Beziehung zu Ihrem behandelnden Arzt (Arzt-Patient-Beziehung) fürchten.

Wenn Sie sich entscheiden, an dieser Studie teilzunehmen, möchten wir sie höflich fragen, die Einverständniserklärung zu unterschreiben.

Nachdem Sie die Einverständniserklärung unterschrieben haben, haben Sie das Recht, Ihre Teilnahme an der Studie jederzeit zu beenden. Sie müssen keinen Grund angeben, warum Sie Ihre Einwilligung widerrufen, an der Studie teilzunehmen.

Der Prüfarzt kann zu jeder Zeit Ihre Teilnahme an der Studie beenden, und dies ohne Ihre Zustimmung.

Risiken und Unannehmlichkeiten

Von jedem Medikament wird nur eine Dosis (von EPO zwei Dosen) verabreicht werden. Nach ausführlicher Recherche der medizinischen Literatur scheint eine einmalige Verabreichung dieser Medikamente sicher zu sein. Dies wurde auch durch die Ergebnisse der „Safety-Studie“ bestätigt. Dennoch können unerwartete Wechselwirkungen / potenzielle Risiken dieser Medikamenten- Kombination nicht vollständig ausgeschlossen werden. Folgenede Nebenwirkungen können während der Behandlung mit diesen Medikamenten auftreten:

- *Cetor®/Cinryze®*: Allergische oder anaphylaktische Reaktionen (z.B. Herzrasen, hoher oder niedriger Blutdruck, Rötung am Hals und im Gesicht, Nesselsucht, Atembeschwerden)
- *Atenativ®*: Allergische Reaktionen oder Überempfindlichkeit, erhöhte Körpertemperatur und Erweiterung von Blutgefäßen
- *Vitamin E Lösung®*: keine bekannten Nebenwirkungen
- *Tationil® 600*: keine bekannten Nebenwirkungen
- *Circadin®*: keine bekannten Nebenwirkungen
- *Apotransferrin*: keine bekannten Nebenwirkungen
- *Neorecormon®*: Bildung eines Blutgerinnsels in einem Blutgefäß, Epilepsie und Bluthochdruck
- *Remicade®*: Hautausschlag, Anomalien in der Zusammensetzung des Blutes und lebensbedrohliche allergische Reaktionen
- *Flolan®*: Kopfschmerzen, Übelkeit, niedriger Blutdruck, langsamer Herzschlag, Herzrasen und Kurzatmigkeit

Vorteile

Wir können die therapeutische Wirkung der Medikamente nicht garantieren, auch wenn wir

CONFIDENTIAL

wissen, daß jedes Arzneimittel für sich den Ausmass des Ischämie-Reperfusionsschadens zu reduzieren vermag.

Versicherung

In Übereinstimmung mit der belgischen Gesetzgebung zu Untersuchungen an der menschlichen Person vom 7. Mai 2004 der Auftraggeber/Sponsor einer klinischen Untersuchung haftbar für alle Schäden (auch im Falle einer Schuldlosigkeit), die dem Teilnehmer und / oder seinem Rechtsnachfolger entstanden sind und die direkt oder indirekt mit der Studie verbunden sind. Der Sponsor hat eine Versicherung abgeschlossen, die diese Haftung abdecken wird. Falls bei Ihnen als Folge Ihrer Teilnahme an dieser Studie ein Schaden auftreten sollte, wird Ihnen dieser Schaden daher in Übereinstimmung mit der belgischen Gesetzgebung zu Untersuchungen an der menschlichen Person vom 7. Mai 2004 erstattet werden.

Entschädigungen

Durch diese Studie werden Ihnen keine zusätzlichen Kosten entstehen. Sie müssen die Studienmedikamente nicht bezahlen. Darüber hinaus werden alle Untersuchungen von Blut-, Urin- und Gewebeproben, die nicht routinemäßig durchgeführt werden, durch die Untersucher bezahlt.

Der Schutz Ihrer Privatsphäre

Ihre Identität und die Teilnahme an dieser Studie werden streng vertraulich behandelt werden. Sie werden nicht mit Ihrem Namen oder auf andere Weise in den Studien-Dokumenten, Ergebnissen oder Veröffentlichungen identifiziert werden können.
Ihre medizinischen Unterlagen werden von Vertretern der Prüfärzte und durch die Aufsichtsbehörden, die die Studie überwachen, untersucht werden. Dies erfolgt im Einklang mit den GCP = Good Clinical Practice - Richtlinien. Hierbei wird Ihre Identität durch eine einzigartige Patientennummer geheim gehalten, die lediglich Rückschluss auf wenige persönliche Daten erlaubt.
Ihre personenbezogenen Daten können an die Aufsichtsbehörden, die Ethik-Kommission und andere Ärzte, die mit den Prüfärzten kooperieren, offen gelegt werden.
Ihre persönlichen Daten werden elektronisch oder manuell verarbeitet und analysiert, um die Ergebnisse dieser Studie auszuwerten. Sie haben das Recht, vom Prüfarzt Auskunft über die gesammelten Daten und den Zweck dieser Daten zu erhalten. Sie haben auch das Recht auf Einsicht in Ihre persönlichen Daten und so nötig, zur Korrektur derselben.
Alle Vorschriften zum Schutz personenbezogener Daten sind im Gesetz zum Schutz der Privatsphäre vom 8. Dezember 1992 (Belgien) beschrieben.

Neue Informationen

Manchmal ist es möglich, dass neue Informationen über die im Rahmen der klinischen Studie angewandten Therapien oder Arzneimittel bekannt werden. Wenn dies der Fall ist, werden Sie über diesen neuen Kenntnisstand informiert werden, wenn dies Ihre Bereitschaft zur weiteren Teilnahme an dieser Studie beeinflussen könnte. In diesem Fall werden Sie aufgefordert, eine neue Informationsbroschüre und eine neue Einverständniserklärung zu

CONFIDENTIAL

unterschreiben.

Ethik-Kommission

Die unabhängige medizinische Ethikkommission des „UZ KU Leuven / Forschung“ hat diese Studie geprüft und genehmigt, ebenso die lokalen Ethikkommissionen der Zentren, in denen diese Studie durchgeführt wird.

Kontaktperson für Fragen über diese Studie

Wenn Sie glauben, Schäden durch diese Studie erlitten zu haben, eine unerwünschte Reaktion auf ein Studienmedikament durchgemacht zu haben oder Sie Fragen zu der Studie haben, können Sie sich jetzt, während und / oder nach der Studie, jederzeit an den leitenden Prüfarzt wenden:

Principal investigator: Prof. dr. Diethard Monbaliu
Adresse: Abdominale transplantatiechirurgie, UZ Leuven
Herestraat 49 bus 7003, 3000 Leuven
Telefon Sekretariat: + 32 16 348727
Fax Sekretariat: + 32 16 348743

Studienkoordinator: Sarah Mertens
Adresse: Abdominale transplantatiechirurgie, UZ Leuven
Herestraat 49 Bus 7003, 3000 Leuven
Telefon: + 32 16 342961

CONFIDENTIAL

APPENDIX 9: CHRONOLOGICAL IDENTIFICATION LIST

NAME	UNIQUE NUMBER	INITIALS	DATE OF BIRTH	AGE	SEX	DATE OF ENROLMENT	WITHDRAWAL

CONFIDENTIAL

APPENDIX 10: CHRONOLOGICAL SCREENING LIST

NAME	INITIALS	D.O.B	AGE	SEX	DATE OF TRANSPLANTATION	EXCLUSION CRITERIA

CONFIDENTIAL

APPENDIX 11: PARTICIPATING CENTERS

**Center**	**Country**	**City**	**Principal investigator**
1.	Belgium	Leuven	D. MONBALIU

CONFIDENTIAL

APPENDIX 12: SIGNATURE PAGE

Signature page

"We, the signatories, confirm that this clinical trial protocol contains all the information and regulations necessary for the conduct of this particular trial. We sign the protocol as an agreement of the details of the clinical trial and the means of data recording. We commit ourselves to comply with all instructions, regulations and agreements as laid down in this clinical trial protocol and in the EC-GCP guidelines and applicable national laws and regulations. We certify that our local ethics committee has seen and accepted the protocol."

Name, date and signature of investigator

Name, date and signature of co-investigator

CONFIDENTIAL

CONFIDENTIAL
