# Supplementary material for: Effect of a Combined Drug Approach on the Severity of Ischemia-Reperfusion Injury During Liver Transplant: A Randomized Clinical Trial
Source: JAMA Netw Open. 2023 Feb 28;6(2):e230819. doi: 10.1001/jamanetworkopen.2023.0819 (PMC9975910; doi:10.1001/jamanetworkopen.2023.0819)
Supplement: Supplement 2. — eTable 1. Surgical Complications eTable 2. Adverse Events and Serious Adverse Events [file jamanetwopen-e230819-s002.pdf]

Supplementary Online Content

Meurisse N, Mertens M, Fieuws S, et al. Effect of a combined drug approach on the severity of ischemia-reperfusion injury during liver transplant: a randomized clinical trial. *JAMA Netw Open*. 2023;6(2):e230819. doi:10.1001/jamanetworkopen.2023.0819

eTable 1. Surgical Complications

eTable 2. Adverse Events and Serious Adverse Events

This supplementary material has been provided by the authors to give readers additional information about their work.

© 2023 Meurisse N et al. *JAMA Network Open*.

eTable 1. Surgical Complications (Clavien-Dindo Classification)

		CDA group (n=36)		Control group (n=36)	
		Surgical complications (N)	Severe surgical complications (≥ 3b) (n)	Surgical complications (N)	Severe surgical complications (≥ 3b) (n)
**Medical complications**	Kidney dysfunction	13	1	14	2
	Ascites	2	0	0	0
	Pleural effusion	3	0	5	0
	Delirium	0	0	0	0
	Urinary infection	4	0	4	0
	De novo diabetes	0	0	0	0
	Respiratory insufficiency	2	1	3	1
	Other	0	0	1	0
**Viral infections**	CMV	0	0	1	0
	EBV	0	0	0	0
	HBV	0	0	0	0
	HCV	0	0	0	0
	HSV type 1	0	0	0	0
	HSV type 2	0	0	0	0
	Varicella zoster	0	0	0	0
	Influenza	0	0	0	0
	Polyoma virus	0	0	0	0
	Other	0	0	0	0
**Bacterial infections**	Pneumonia	4	0	4	0
	Gastro-intestinal	0	0	0	0
	Clostridium difficile	1	0	3	0
	Gastro-intestinal, other	0	0	0	0
	Sepsis	2	2	1	1
	Other	16	0	16	0
	Aspergillus fumigatus	1	0	0	0

© 2023 Meurisse N et al. *JAMA Network Open*.

**Fungi infections**	Candida albicans	2	0	0	0
	Cryptococcus neoformans	0	0	0	0
	Other	0	0	0	0
**Parasite infections**	Toxoplasmose gondii	0	0	0	0
	Trypanosoma cruzii	0	0	0	0
	Other	0	0	0	0
**Thrombo-embolic complications**	Pulmonary embolism	0	0	0	0
	Deep venous thrombosis	0	0	0	0
	Other	0	0	0	0
**Cardio-vascular events**	Infarct	0	0	0	0
	De novo heart failure	0	0	1	0
	Arrythmia	6	1	5	0
**Cerebro-vascular events**	Ischemic	0	0	0	0
	Hemorragic	0	0	0	0
**Liver graft complications**	Donor preservation solution infection	10	0	6	0
	Donor aorta patch infection	8	0	9	0
	Perfusate solution infection	0	0	0	0
**Surgical arterial problems**	Hepatic artery thrombosis	0	0	0	0
	Hepatic artery stenosis	0	0	0	0
	Mycotic aneurysm	0	0	0	0
**Caval and hepatic veins complications**	Caval vein thrombosis	0	0	0	0
	Caval vein stenosis	0	0	0	0
	Hepatic vein thrombosis	0	0	0	0
**Portal vein complications**	Portal vein thrombosis	0	0	1	1
	Portal vein stenosis	0	0	0	0

© 2023 Meurisse N et al. *JAMA Network Open*.

**General surgical complications**					
	Bleeding	2	2	3	3
	Superficial wound dehiscience	7	3	6	0
	Wound infection	3	0	3	1
	Evisceration	0	0	1	1
	Seroma	5	0	1	0
	lymphocele	1	1	1	1
	Other	6	2	1	1
**Bile ducts complications**	Bile leak	0	0	0	0
	Anastomotic strictures	0	0	0	0
	Ischemic cholangiopathy	0	0	0	0
	Choledocolithiasis	0	0	0	0
	Cholangitis	0	0	0	0
**Immunological complications**	Acute antibody-mediated rejection	0	0	0	0
	Acute cellular rejection	7	0	7	0
Ratio of severe surgical complications, n/N (%) (p>0.99)		13/105 (12,4%)		12/97 (12,4%)	

Abbreviations: CDA, combined drugs approach; CMV, cytomegalovirus; EBV, Epstein-Barr virus; HBV, hepatitis B virus; HCV, hepatitis C virus; HSV, herpes simplex virus

© 2023 Meurisse N et al. *JAMA Network Open*.

eTable 2. Adverse Events and Serious adverse Events

		CDA group n=36		Control group n=36	
		Adverse event	Serious adverse event	Adverse event	Serious adverse event
**Medical complications**	Kidney dysfunction	9	1	10	0
	Ascites	0	0	0	0
	Pleural effusion	3	0	4	0
	Delirium	0	0	0	0
	Urinary infection	3	0	2	0
	De novo diabetes	0	0	0	0
	Respiratory insufficiency	1	1	2	1
	Other	3	0	4	0
**Viral infections**	CMV	0	0	0	0
	EBV	0	0	0	0
	HBV	0	0	0	0
	HCV	0	0	0	0
	HSV type 1	0	0	0	0
	HSV type 2	0	0	0	0
	Varicella zoster	0	0	0	0
	Influenza	0	0	0	0
	Polyoma virus	0	0	0	0
	Other	0	0	0	0
**Bacterial infections**	Pneumonia	2	0	2	0
	Gastro-intestinal	0	0	0	0
	Clostridium difficile	0	0	0	0
	Gastro-intestinal, other	0	0	0	0
	Sepsis	1	1	0	0
	Other	2	0	0	0

© 2023 Meurisse N et al. *JAMA Network Open*.

**Fungi infections**	Aspergillus fumigatus	0	0	0	0
	Candida albicans	1	0	0	0
	Cryptococcus neoformans	0	0	0	0
	Other	0	0	0	0
**Parasite infections**	Toxoplasmose gondii	0	0	0	0
	Trypanosoma cruzii	0	0	0	0
	Other	0	0	0	0
**Thrombo-embolic complications**	Pulmonary embolism	0	0	0	0
	Deep venous thrombosis	0	0	0	0
	Other	0	0	0	0
**Cardio-vascular events**	Infarct	0	0	0	0
	De novo heart failure	0	0	1	0
	Arrythmia	5	1	4	0
**Cerebro-vascular events**	Ischemic	0	0	0	0
	Hemorragic	0	0	0	0
**Liver graft complications**	Donor preservation solution infection	10	0	6	0
	Donor aorta patch infection	8	0	9	0
	Perfusate solution infection	0	0	0	0
**Surgical arterial problems**	Hepatic artery thrombosis	0	0	0	0
	Hepatic artery stenosis	0	0	0	0
	Mycotic aneurysm	0	0	0	0
**Caval and hepatic veins complications**	Caval vein thrombosis	0	0	0	0
	Caval vein stenosis	0	0	0	0
	Hepatic vein thrombosis	0	0	0	0
**Portal vein complications**	Portal vein thrombosis	0	0	1	1
**General surgical complications**	Portal vein stenosis	0	0	0	0
	Bleeding	1	1	2	2

© 2023 Meurisse N et al. *JAMA Network Open*.

	Superficial wound dehiscience	0	0	0	0
	Wound infection	0	0	1	0
	Evisceration	0	0	0	0
	Seroma	0	0	0	0
	lymphocele	0	0	0	0
	Other	7	1	3	0
**Bile ducts complications**	Bile leak	0	0	0	0
	Anastomotic strictures	0	0	0	0
	Ischemic cholangiopathy	0	0	0	0
	Choledocolithiasis	0	0	0	0
	Cholangitis	0	0	0	0
**Immunological complications**	Acute antibody-mediated rejection	0	0	0	0
	Acute cellular rejection	1	0	0	0
Total number of adverse events (severe adverse events)		57 (6)		51 (4)	

Abbreviations: CDA, combined drugs approach; CMV, cytomegalovirus; EBV, Epstein-Barr virus; HBV, hepatitis B virus; HCV, hepatitis C virus; HSV, herpes simplex virus

© 2023 Meurisse N et al. *JAMA Network Open*.
