# Supplementary material for: Effect of a Combined Drug Approach on the Severity of Ischemia-Reperfusion Injury During Liver Transplant: A Randomized Clinical Trial
Source: JAMA Netw Open. 2023 Feb 28;6(2):e230819. doi: 10.1001/jamanetworkopen.2023.0819 (PMC9975910; doi:10.1001/jamanetworkopen.2023.0819)
Supplement: Supplement 3. — Data Sharing Statement [file jamanetwopen-e230819-s003.pdf]

Data Sharing Statement

Meurisse. Effect of a Combined Drug Approach on the Severity of Ischemia-Reperfusion Injury During Liver Transplant. *JAMA Netw Open*. Published February 28, 2023.
doi:10.1001/jamanetworkopen.2023.0819

Data
Data available: Yes
Data types: Deidentified participant data
How to access data: diethard.monbaliu@uzleuven.be
When available: With publication

Supporting Documents
Document types: None

Additional Information
Who can access the data: researchers whose proposed use of the data has been approved
Types of analyses: for a specified purpose
Mechanisms of data availability: signed data access agreement
Any additional restrictions: no additional restrictions
